# Porous matrix materials in optical sensing of gaseous oxygen

**DOI:** 10.1007/s00216-022-04014-6

**Published:** 2022-03-29

**Authors:** I. Dalfen, S. M. Borisov

**Affiliations:** Institute of Analytical Chemistry and Food Chemistry, Graz University of Technology, Stremayrgasse 9, 8010 Graz, Austria

**Keywords:** Oxygen, Fluorescence, Phosphorescence, Sensor, Porosity, Sensitivity

## Abstract

The review provides comparison of porous materials that act as a matrix for luminescent oxygen indicators. These include silica-gels, sol–gel materials based on silica and organically modified silica (Ormosils), aerogels, electrospun polymeric nanofibers, metal–organic frameworks, anodized alumina, and various other microstructured sensor matrices. The influence of material structure and composition on the efficiency of oxygen quenching and dynamic response times is compared and the advantages and disadvantages of the materials are summarized to give a guide for design and practical application of sensors with desired sensitivity and response time.

**Graphical abstract:**

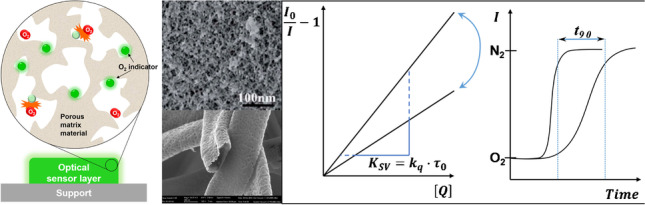

**Supplementary Information:**

The online version contains supplementary material available at 10.1007/s00216-022-04014-6.

## Introduction

In the past decades, the field of luminescence-based sensing of oxygen has evolved from an emerging discipline to a well-established technology [1, 2]. While in the 1990s and early 2000s new luminescent indicators and matrices for optical oxygen sensing were discovered at a high rate, the more recent research has been focused on fine-tuning and modification of sensor components to achieve ever increasing sensor performance for a wide variety of applications in (marine) biology, oceanography, biotechnology, medicine, and many other fields [3–6]. Understanding the role of individual components in the sensor performance is a critical prerequisite for efficiently designing optical oxygen sensors with the desired properties for a particular application. Oxygen indicator and matrix, in which it is immobilized, are the most important components that determine the spectral properties of the sensor, its brightness, sensitivity to oxygen, dynamic response, (photo)stability etc. Indicators for oxygen sensors have been discussed in detail elsewhere [7], the same refers to the quite common non-porous matrices (e.g., polymers like polystyrene or silicone rubber) [2].

Porous materials have served as important matrices for oxygen sensing. Their common feature is comparably fast oxygen diffusion which enhances the sensitivity of the sensors and improves response times. Among the first porous matrices used in oxygen sensing were silica-gels, amorphous SiO_2_ particles that are commercially available in a wide range of porosities and particle sizes. Modification of silica-gel to include organic groups via Si–O-R bonds results in organically modified silica, in short Ormosil. Ormosils have been popular as matrix materials from the early 1990s until today because of versatility in terms of tuneability of their porosity and hydrophobicity. Similar to silica-gels, aluminum oxides have been used as porous support for oxygen indicator dyes. Some groups have exploited the uniform (nano-)porosity that can be achieved in anodized alumina. Electrospun nanofibers and metal–organic frameworks emerged as new materials for optical oxygen sensing during the last 10 to 15 years and though being quite different from a chemical point of view, they share the desirable features of high versatility and tuneability of structure and composition. Parallel to and sometimes in combination with these porous materials, non-porous polymers have been used as matrices for optical oxygen sensors. The most noticeable being polystyrene, poly(methyl methacrylate), silicone rubber, and perfluorinated polymers, which will be discussed to compare the performance of porous and non-porous matrix materials.

This review is dedicated to highlighting the effect of matrix porosity on sensor performance, more specifically on the sensitivity and dynamic response of oxygen sensors. Examples of oxygen sensors for each group of materials will be provided along with comparison between different groups of porous and non-porous matrices in terms of sensitivity and response time but also handling and practical applicability. An electronic supporting information is provided with information on the commercial availability of the different matrix materials (Table S1) and data on sensitivity, bimolecular quenching constant, and response time of all materials discussed within this review (Tables S2–S9).

## General considerations

### Quenching of luminescence by molecular oxygen

**Fig. 1:**
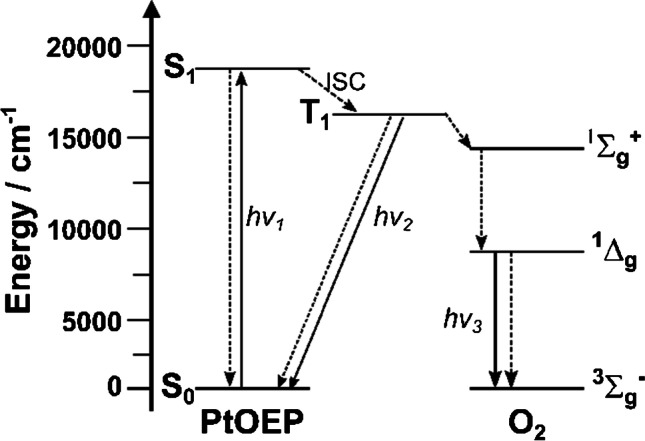
Simplified Jablonski diagram illustrating luminescence quenching of a common oxygen indicator PtOEP by molecular oxygen. After excitation into a singlet excited state (directly to S_1_ or into S_2_, following radiativeless deactivation into S_1_) and inter-system crossing to T_1_ state, energy transfer results in formation of singlet oxygen and its subsequent deactivation into the triplet ground level

The quenching of luminescence by oxygen may happen via different pathways, the exact mechanism depending on the specific conditions [8, 9]. The most important deactivation mechanism is energy transfer via an electron-exchange Dexter mechanism. Energy transfer from both singlet excited state (S_1_) and triplet excited state (T_1_) is spin-allowed so that the dioxygen molecule can efficiently quench fluorescence and phosphorescence, respectively. For most of (metal)organic dyes, the energy level of the excited state (S_1_ or T_1_) is above the ${}^{1}{\sum }_{g}^{+}$ level of oxygen and is always above the ${}^{1}{\Delta }_{g}$ level (Fig. 1) indicating the possibility of energy transfer to any of these states. However, even if the ${}^{1}{\sum }_{g}^{+}$ level of oxygen is originally populated, fast deactivation leads to formation of ${}^{1}{\Delta }_{g}$ singlet oxygen which lifetime typically varies from about 3 µs in water to tens of microseconds in polymers and even much longer in gas phase.

As the energy transfer is a very fast process, the rate of oxygen quenching is limited by the rate of oxygen diffusion leading to an encounter between oxygen and dye molecule (*k*_diff_). The decay times of fluorescent emitters typically are in order of several nanoseconds and therefore no significant quenching by oxygen is observed in most conditions. If oxygen diffusion, however, is very fast in a given matrix, even fluorescence may be efficiently quenched by oxygen.

### Sensitivity

In an ideal case of dynamic quenching of the luminophore’s excited state (triplet or singlet) through oxygen, the relationship between intensity (or luminescence decay time) and oxygen concentration is described by the Stern–Volmer equation [10, 11]:

1 $$\frac{{I}_{0}}{I}=\frac{{\tau }_{0}}{\tau }=1+{K}_{SV}\bullet \left[{O}_{2}\right]$$

with

2 $$\left[{O}_{2}\right]={S}_{{O}_{2}}\bullet p{O}_{2}$$

where *I*_0_/*τ*_0_ and *I/τ* are the luminescence intensity/lifetime in the absence of oxygen and at a given concentration of oxygen [*O*_2_] in *mol L*^*−1*^, respectively, which is the product of oxygen partial pressure *pO*_2_ and oxygen solubility in the sensor matrix ${S}_{{O}_{2}}$, and *K*_*SV*_ is the Stern–Volmer constant which is specific for a sensor under given conditions (temperature, humidity etc.) and includes different parameters that determine the oxygen sensitivity of a given sensor [11, 12].

3 $${K}_{SV}={k}_{q}\bullet {\tau }_{0}$$

4 $${k}_{q}=\frac{4\pi {N}_{A}}{1000}\bullet {{R}_{C}\bullet p\bullet D}_{{O}_{2}}$$

where *k*_*q*_ is the rate constant of the bimolecular quenching process in *L mol*^*−1*^* s*^*−1*^, *N*_*A*_ is Avogadro’s number, *R*_*C*_ is the collision radius of the oxygen indicator complex in *cm*, *p* is the efficiency of quenching (*p* = 1 for diffusion controlled systems), and ${D}_{{O}_{2}}$ is the diffusion coefficient of oxygen in the matrix in *cm*^*2*^*s*^*−1*^ (given that the diffusion of the immobilized indicator is virtually negligible compared to the diffusion of oxygen).

It is commonly considered that *k*_*q*_ approaches the diffusion controlled limit (*k*_diff_) only in case of fluorescent indicators (quenching of the excited singlet state) whereas for phosphorescent dyes (quenching of the excited triplet state), *k*_*q*_ is reduced to 1/9 *k*_diff_ due to the spin-statistical factor [8]. The encounter of a phosphorescent dye in the excited triplet state (T_1_) and an oxygen molecule in its ground state (${}^{3}{\sum }_{g}^{-}$) leads to the reversible formation of an excited complex (${T}_{1}{}{}^{3}\sum$) for which nine spin configurations are possible. However, only one of these configurations allows for energy transfer from the dye to the oxygen molecule, leading to ninefold reduction of *k*_*q*_ compared to *k*_diff_. It is therefore essential to keep in mind the nature of the involved emissive state when comparing the *k*_*q*_ constants calculated from the experimental data (*K*_*sv*_/*τ*_0_).

However, even within the same class of indicator dyes, the situation may be more complex than expected. For instance, Han et al. compared several common oxygen indicator dyes adsorbed on SBA-15 mesoporous silica and after correction for different excited state lifetimes, the dye specific part of *k*_*q*_ (*R*_*c*_*·p)* was found to vary by as much as 3.3-fold between the dye PdOEP (the lowest *k*_*q*_) and Ru(phen)_3_^2+^ (the highest *k*_*q*_) [13]. The reason for this may be difference in distribution of the dyes on the surface of the material resulting in variation of surface concentration of the quencher. It should also be mentioned that some dyes (such as cyclometalated Ir(III) complexes) in addition to quenching via energy transfer from dye to molecular oxygen also may show quenching via the electron transfer reaction resulting in formation of dye cation radical and superoxide ion. This results in the higher *k*_*q*_ values compared to the one expected from the *τ*_0_ value and purely energy transfer mechanism [14]. Nevertheless, with bimolecular quenching constants ranging many orders of magnitude (10 to 10^5^ Pa^−1^ s^−1^) for the majority of materials investigated in this review, the dye-related factors can be considered minor compared to oxygen permeability of the matrix. Therefore, in this review, the bimolecular quenching constant will be used as a measure of quenching efficiency to compare different matrices without correcting for the nature of the indicator used in the respective studies.

If the quenching is strictly dynamic and the indicator located in a homogenous matrix with only one microenvironment guaranteeing the same accessibility to oxygen for each indicator molecule, the plot of $\frac{{I}_{0}}{I}$ or $\frac{{\tau }_{0}}{\tau }$ vs. [*O*_2_] gives a straight line with the slope *K*_*SV*_ (Eq. 1). In contrast to the solutions of indicators in organic solvents where quenching obeys Eq. 1, most solid matrices are characterized by different dye microenvironments corresponding to different accessibility of the dye to oxygen. This leads to a deviation from a linear Stern–Volmer plot resulting in a plot with downward curvature. Different models describing such behavior have been proposed such as Lehrer model [15] and Demas two site model [16]. The latter postulates location of the indicator in two microenvironments characterized by the Stern–Volmer constants *K*_*SV*1_ and *K*_*SV*2_ (Eq. 5)

5 $$\frac{{I}_{0}}{I}={\left(\frac{f}{1+{K}_{SV1}\left[{O}_{2}\right]}+\frac{1-f}{1+{K}_{SV2}\left[{O}_{2}\right]}\right)}^{-1}$$

where *f* is the relative contribution of the first microenvironment.

### Response time

The response time of a sensor is generally defined as the time required for the signal to reach 90% or 95% of the new equilibrium value after changing the analyte concentration (denoted as *t*_90_ and *t*_95_, respectively). As the quenching reaction itself is very fast compared to gas diffusion through the matrix, the response time of a sensor will be determined by the oxygen diffusion coefficient as well as the thickness of the sensing layer.

The reported response times have to be handled with care. The reason for this are the experimental limitations since the atmosphere in the measurement chamber typically is not changed abruptly. Particularly in case of fast-responding sensors, the reported decay times may reflect the time required for the gas exchange in the chamber and thus be strongly overestimated.

In most cases, the recovery time (changing from oxygenated to oxygen-free conditions) is reported to be significantly longer than the response time (changing from oxygen-free to oxygenated conditions). In some cases, e.g., on silica surfaces, this might partially be explained by O_2_ adsorption on the surface. Mostly, however, this is an artifact as quenching is not linear over the whole concentration range of oxygen and quenching is significant already at low oxygen concentration. Evidently, in case of trace sensors, which upper limit is reached at oxygen concentrations well below air saturated conditions, it makes little sense to determine the response and recovery times via cycling between 100% oxygen and oxygen-free atmosphere.

## Silica-gel-based materials

### Inorganic silica backbone

**Fig. 2:**
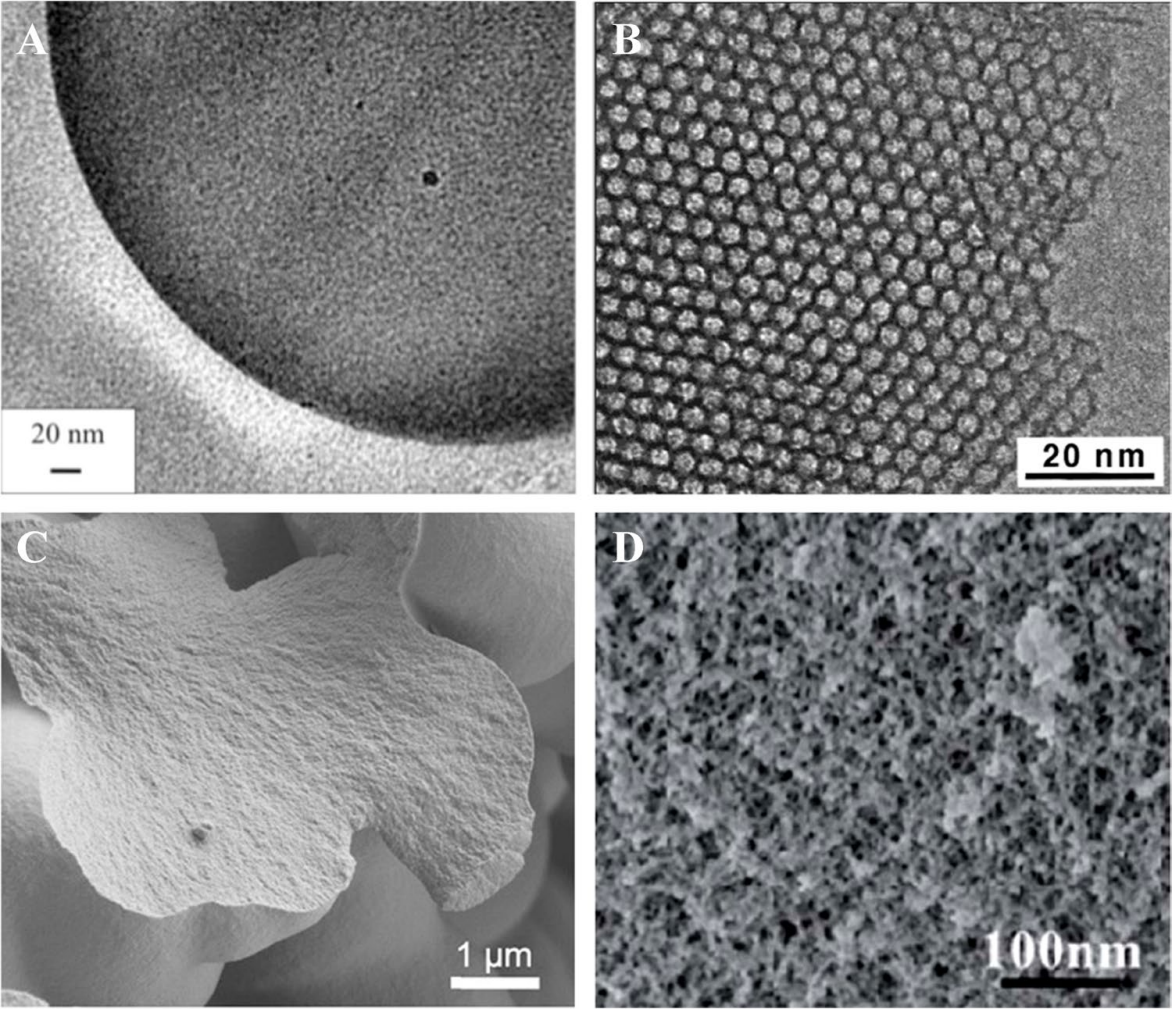
Electron microscopy images of common silica-based porous materials. **A** TEM image of a silica-gel particle. Reprinted with permission from Ref. [17], Copyright 2012, American Chemical Society; **B** TEM image of mesoporous MCM-41 material. Reprinted with permission from Ref. [18]. Copyright 2000, American Chemical Society; **C** SEM image of an organically modified silica (Ormosil) particle. Reprinted from Ref. [19], Copyright 2012, with permission from Elsevier; **D** SEM image of a silica aerogel. Reprinted with permission of the Royal Society of Chemistry from Ref. [20] permission conveyed through Copyright Clearance Center, Inc

Porous materials with inorganic silica backbone can be divided into several subclasses:

- Mesoporous silica-gel particles. They are characterized by a random pores network with an average pore size varying from 35 to 250 Å, with the most common representative having the pore size of 60 Å (Fig. 2A).
- Materials with mesoporous hierarchical porous structure. Most common representatives are MCM-41 (Fig. 2B) and SBA-15. Since these materials are prepared by hydrolysis of precursors in presence of surfactant, the pore size depends on the surfactant used. The pores of MCM-41 are typically smaller (20–65 Å) compared to SBA-15 (50–150 Å).
- Macroporous controlled pore glass CPG (pore diameter of hundreds of Å).

Inorganic silica materials were among the first matrices to be used in optical oxygen sensing [21–23]. Cationic dyes, mainly Ru(II) polypyridyl complexes, can simply be adsorbed on the surface of the porous silica via electrostatic interactions. In more complex systems, covalent immobilization on the modified silica surface or encapsulation within the pores during sol–gel synthesis is possible.

In silica-based materials, quenching happens through collisions between the adsorbed/immobilized indicator with both gaseous and surface-adsorbed oxygen [13, 22]. Surface adsorption of oxygen, following a Langmuir-Hinshelwood model, therefore increases the quenching probability for surface-immobilized indicator molecules and can become the dominating quenching pathway in case of inorganic silica surfaces [16, 24]. This leads to a deviation from Henry’s law since the surface oxygen concentration is related to the oxygen partial pressure via an adsorption isotherm [25]. Since other surface-adsorbed molecules such as water or alcohols represent obstacles for the diffusion of adsorbed oxygen, they interfere with quenching, which results in *cross-sensitivity to humidity* in most silica-based sensor materials. This cross-talk is in some cases so pronounced that a humidity sensor can be designed based on this principle. In fact, Posch and Wolfbeis reported a humidity sensor (albeit with an oxygen cross-talk) based on a perylene-bisimide dye adsorbed on silica-gel TLC plates [26]. While the oxygen cross-talk with a Stern–Volmer constant *k*_*q*_ of only 0.08 kPa^−1^ seems small, the bimolecular quenching constant for oxygen in this sensor is as high as 16,000 Pa^−1^ s^−1^ indicating the suitability of silica-gel materials for oxygen sensing.

The most straightforward way of indicator immobilization on porous silica-gel particles is to utilize *electrostatic attraction of cationic dyes* (most commonly Ru(II) polypyridyl complexes) to the silica surface which efficiently prevents dye leaching and aggregation. These materials are usually characterized by nonlinear SV plots and show medium to high sensitivity to molecular oxygen. The bimolecular quenching constants (*k*_*q*_) are typically in range 40–200 Pa^−1^ s^−1^. Indicator-doped porous silica-gel particles are seldom applied on their own. Rather, they are dispersed in a host polymer featuring high gas permeability and hydrophobic character, such as silicone rubber. As shown by He et al., short-chain PDMS prepolymers and crosslinkers may penetrate into the pores and reduce sensitivity. In the described sensors, *K*_*SV*_ decreased from 0.03 to 0.02 kPa^−1^ and *k*_*q*_ from 30 to 20 Pa^−1^ s^−1^ when going from a long-chain length to a short-chain length crosslinker [27]. Nevertheless, immobilization of the dye in porous particles as a matrix is shown to result in more sensitive sensors compared to those obtained by dye immobilization directly into silicone rubber. For instance, Klimant et al. reported *k*_*q*_ constants of ~ 120 Pa^−1^ s^−1^ for Ru(II) polypyridyl complexes adsorbed on silica-gel and then dispersed in silicone matrix, whereas the dyes directly immobilized into the same silicone gave lower *k*_*q*_ of ~ 30 Pa^−1^ s^−1^ [28].

Morphology and surface chemistry of the materials appear to be important parameters that affect the sensitivity of silica-based materials. For instance, in case of electrostatically immobilized Ru(II) indicator, the sensitivity increased strongly on going from reversed phase silica-gel (*K*_*SV*_ 0.02 kPa^−1^, *k*_*q*_ 40 Pa^−1^ s^−1^) to regular silica-gel (*K*_*SV*_ 0.10 kPa^−1^, *k*_*q*_ 210 Pa^−1^ s^−1^) and controlled pore glass (*K*_*SV*_ 0.14 kPa^−1^, *k*_*q*_ 290 Pa^−1^ s^−1^) [29]. The sensitivity was the lowest for indicator immobilized directly into silicone rubber (*K*_*SV*_ 0.01 kPa^−1^, *k*_*q*_ 10 Pa^−1^ s^−1^) [29], which is in good agreement with results obtained by Klimant and co-workers [28].

An obvious strategy to further enhance the sensitivity of silica-gel-based oxygen sensors is to exchange the Ru(II) polypyridyl complexes which possess relatively short-lived luminescence (*τ*_0_ of about 6 µs for Ru(II) (4,7-diphenyl-1,10-phenanthroline) (= Ru(dpp)_3_) with indicators featuring longer phosphorescence decay time. Thus phosphorescent Pt(II) and Ir(III) porphyrins (*τ*_0_ in the range of tens of microseconds) and particularly Pd(II) porphyrins (*τ*_0_ of hundreds of microseconds) are useful for preparation of much more sensitive sensors. For instance, Koren et al. immobilized an Ir(III)OEP complex with bulky axial ligands on silica-gel [30] and obtained *k*_*q*_ value of ~ 70 Pa^−1^ s^−1^ which is comparable to that of the Ru(II) based materials but corresponds to significantly higher *K*_*SV*_ of 1.8 kPa^−1^ due to the longer luminescence decay time of the metalloporphyrin (~ 30 µs).

*Covalent immobilization* of indicators to the surface of the porous material completely eliminates indicator leaching and is particularly useful for immobilization of uncharged or negatively charged indicators. For this purpose, the silica surface can be easily modified with amino groups to couple indicators like Erythrosine B that possess carboxylic group [31]. This phosphorescent dye with *τ*_0_ of ~ 300 µs showed high *K*_*SV*_ of 66 kPa^−1^ (*k*_*q*_ ~ 230 Pa^−1^ s^−1^) and a LOD as low as 0.06 Pa O_2_. Very popular highly photostable oxygen indicators based on Pt(II) and Pd(II) complexes with pentafluorophenylporphyrin can also be covalently immobilized on amino-modified silica-gel via nucleophilic substitution of para-positioned fluorine atom [32]. Compared to Erythrosine B [31], the *k*_*q*_ values were about 3–4 times smaller (55 and 65 Pa^−1^ s^−1^ for the Pt(II) and Pd(II) complex, respectively) [32]. Nevertheless, the sensitivity is rather high (*K*_*SV*_ 4 and 63 kPa^−1^, respectively) due to long luminescence decay times of 71 and 980 µs, respectively, that enables LODs as low as 0.25 and 0.015 Pa O_2_, respectively. In contrast to majority of oxygen sensors based on silica-gels, the Stern–Volmer plots are *linear*. The authors also showed that further modification of the silica-gel surface with (3,3,3-trifluoropropyl)methyldimethoxysilane leads to a drastic decrease of temperature and humidity cross-talk while retaining the favorable sensitivity, response time, and linear SV plot. The response times (*t*_100_) for the particles immobilized into silicone rubber were very fast (0.15–0.25 s) despite rather thick sensing layers (~ 25 µm). The group of Melnikov and co-workers also reported linear Stern–Volmer plots for silica-gel-immobilized metalloporphyrins, but *k*_*q*_ constants were significantly lower (~ 7 Pa^−1^ s^−1^) [33]. It should be mentioned here that silica-based nanomaterials have also been reported [34]. These are typically obtained using the Stöber method via hydrolysis of tetraalkoxysilane precursors in presence of oxygen indicators and are applied for intracellular imaging of oxygen distribution.

Along with amorphous silica-gels, highly ordered *mesoporous silicas* such as MCM-41 and SBA-15 proved to be highly promising porous matrices for oxygen sensors. These hierarchical mesoporous silicas are prepared in presence of surfactants and typically show an increase in oxygen sensitivity compared to amorphous silica-gels, as oxygen diffusion in the hexagonal pores is facilitated. Because of the ordered character, high heterogeneity of the microenvironment is not expected. Therefore, nonlinearity of the SV plots may originate from existence of dye aggregates or changes in adsorption of oxygen on the surface upon electrostatic dye immobilization. Indeed, linearity was shown to significantly improve when covalent attachment of the dye is performed [35].

The group of Li published several reports on covalent immobilization of Ru(II) polypyridyl complexes in mesoporous silica, namely MCM-41 (Fig. 2B) and SBA-15, by equipping one ligand with an alkyl-triethoxysilane chain that reacts with TEOS during sol–gel process [35–37]. They found that when going from an amorphous porous material to a hierarchical mesostructured material such as MCM-41 or SBA-15, the sensitivity increases by approximately 1.5-fold and that covalent immobilization gives more linear Stern–Volmer plots. Notably, covalent immobilization during sol–gel process was found to be superior to post-synthetic modification of the mesostructured silica-gel. For the mesoporous MCM-41 and SBA-15 materials and concentration range from 0 to 5% O_2_, the bimolecular quenching constants were around 100–200 Pa^−1^ s^−1^. This value was further significantly increased (*k*_*q*_ ~ 920 Pa^−1^ s^−1^ for the linear range from 0 to 1.4% O_2_) when the Ru(II) complex was bound to MCM-41 through a more rigid anchor [37]. In both cases, the Stern–Volmer plots are not linear.

Nonlinear SV plots with *k*_*q*_ values in the range from 40 to 400 Pa^−1^ s^−1^ for the more sensitive component have also been observed in other works on mesostructured materials MCM-41 [38–43] and SBA-15 [13, 39–41, 43] that incorporated indicators of different classes including Ru(II) [13, 39], Cu(I) [40, 42], and dirhenium [41] complexes as well as metalloporphyrins [13, 38, 43]. This literature indicates that SBA-15 appears to give significantly more sensitive materials with *K*_*SV*_ and *k*_*q*_ being 2–6 times higher than compared to MCM-41. This might be due to larger pore diameter of SBA-15 compared to MCM-41 (10 nm vs. 3 nm). PtTCBPyP immobilized in SBA-15 showed the highest sensitivity for this type of materials with *K*_*SV*_ of 350 kPa^−1^ and *k*_*q*_ of ~ 3500 Pa^−1^ s^−1^ for the more sensitive component of the SV plot [43].

The dynamic response times reported by Zhang et al. for the MCM-41 sensing material were below 1 s [38]. In contrast, most hierarchical, mesostructured silica-based sensors are reported to have response times of a few seconds and recovery times of a few dozens of seconds, but these values appear to be strongly overestimated due to slow gas exchange in the setup.

Since silica-gels as well as ordered mesoporous silica such as MCM-41 and SBA-15 are commonly available as fine powders, further immobilization is necessary to obtain a mechanically robust material for application in oxygen sensing. This immobilization is most commonly achieved by dispersion of the particles in a highly oxygen-permeable polymeric matrix such as silicone rubber [23, 28, 29]. The resulting materials may be employed in different formats including knife- or spin-coated planar films or dip-coated optical fibers. It has to be noted that the dispersion of the sensing particles in a polymeric matrix adds an additional diffusion barrier, thereby slowing down the dynamic response of the sensor. As discussed above, the sensitivity may be affected by the immobilization, if prepolymer or cross-linker molecules penetrate into the silica-gel pores [27]. Although other forms of immobilization, such as using TLC plates where a layer of silica-gel is attached to a solid support via an adhesive [26], or monolayers of SBA-15 adsorbed on a polymer film [13], may provide faster gas diffusion to the sensing particles, this comes at the cost of mechanical stability and flexibility.

### Ormosils

**Fig. 3:**
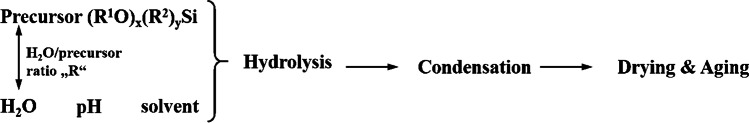
Schematic representation of sol–gel process

Ormosils offer a number of advantages for sensing applications since they can be manufactured in a wide variety of formats, including monoliths, knife-/spin-coated thin films, dip-coated optical fibers, and even microparticles. Figure 3 depicts the sol–gel process and the components involved in the process and influencing the morphology of the final product. Composition of silicon alkoxide precursors largely determines the sensitivity, linearity of calibration plot, and mechanical stability of the resulting materials. However, the conditions (pH) and the ratio of reactants also are important. Particularly, large H_2_O/precursor ratio and high pH are known to give more porous structures with higher specific surface area [12, 44], properties that are associated with higher sensor sensitivity and fast response times.

Tetra(m)ethoxysilane precursors TMOS or TEOS give purely inorganic silica-gel materials after hydrolysis and condensation in the sol–gel process. However, during aging, the sol-gels shrink and often crack, producing mechanically inferior monoliths/sensor layers with lower porosity and oxygen sensitivity than silica-gels [45–49]. Organically modified precursors, where one alkoxide group in TMOS or TEOS is replaced by an organic group with a non-hydrolysable C-Si bond, used in sol–gel process instead of tetra(m)ethoxysilanes favor network termination and generation of defects in the sol–gel network. This in turn creates cavities and increases the porosity of the material (Fig. 2C). Additionally, the surface chemistry of Ormosils is also guided by the nature of organoalkoxysilanes. As shown by McDonagh and co-workers, the sensitivity of the sol–gel sensors is determined by many parameters: sol–gel precursor to water ratio, organic solvent content, aging time, and content of organotriethoxysilane [12, 47, 50]. The higher porosity (associated with faster oxygen diffusion and therefore higher sensitivity) is favored by low water:precursor ratio and slow aging. Modification with methyltriethoxysilane MTEOS results in more flexible networks and faster hydrolysis, which negatively influenced the porosity [12]. The less polar surface also has a negative influence on O_2_ solubility, however, it also reduces the adsorption of water on the sol–gel surface which in turn leads to better O_2_ diffusion. Compared to the hydrophobicity of the surface, the volume fraction porosity has an even stronger influence on the diffusion coefficient. Comparison of the most sensitive MTEOS- and TEOS-based sensors reported in this work reveals about twofold higher sensitivity of the latter with *K*_*SV*_ values of 0.03 and 0.06 kPa^−1^ and *k*_*q*_ values of 7 and 13 Pa^−1^ s^−1^, respectively. Notably, all investigated sensors showed fast response times of < 0.6 s, and even smaller than 0.01 s for the MTEOS-based sensors.

Importantly, preparation method may affect the sensitivity of the resulting materials much more than the chemical composition which was demonstrated by comparison of bulk monoliths and spin-coated thin films prepared from TEOS and MTEOS [51]. The sensitivity of the bulk materials increased with MTMOS content since the chain terminating methyl group promoted formation of cavities in the monolith during slow aging (*K*_*SV*_ 0.02 and 0.3 kPa^−1^ and *k*_*q*_ 5 and 38 Pa^−1^ s^−1^ in pure TEOS and pure MTEOS, respectively). The opposite effect was observed in the spin-coated film, characterized by higher film density at increasing MTMOS content (19.12% volume fraction porosity in TEOS film, 3.14% in MTMOS film) which is explained by faster hydrolysis and higher flexibility of the MTMOS sol-gels. Additionally, the lifetime of the [Ru(dpp)_3_]Cl_2_ indicator varied with the sol–gel composition, ranging from 4.4 µs in pure TEOS to 7.2 µs in pure MTEOS, which also affected the oxygen sensitivity.

**Fig. 4:**
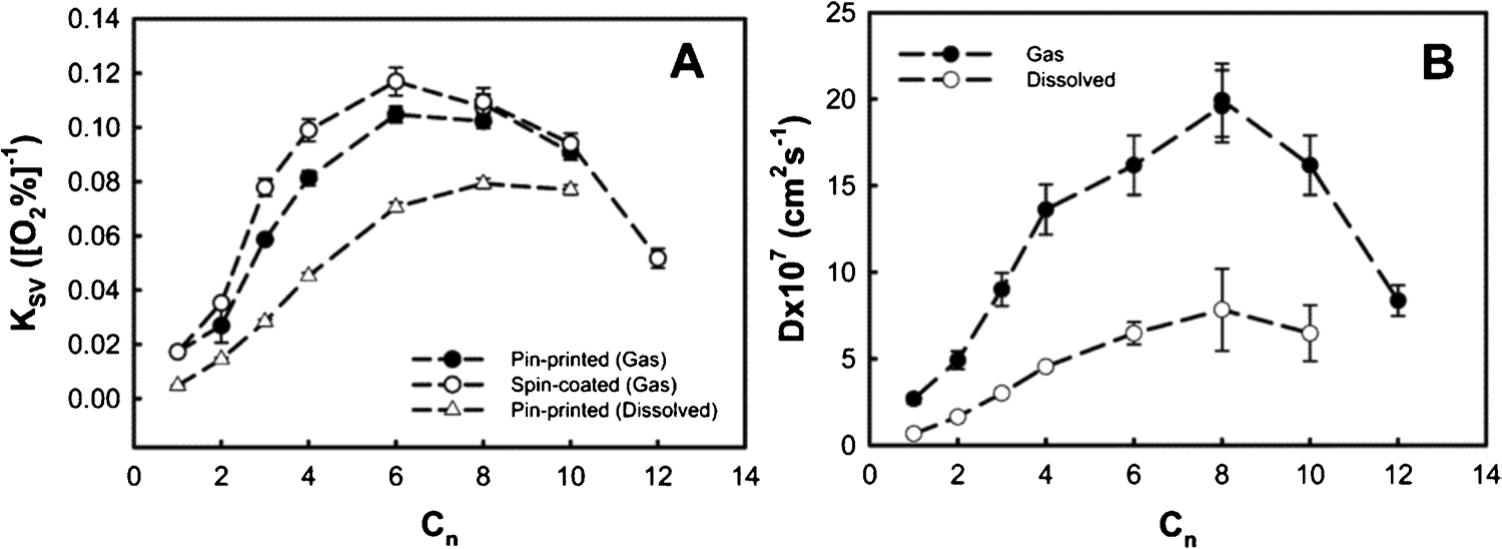
Influence of alkyl chain length in sol-gels of the type (C_*n*_H_2*n*+1_)-Si-(OR)_3_ (R = Et or Me, *n* = 1–12) on the sensitivity (left) and on oxygen diffusion coefficients of the xerogels (right); reprinted with permission from Ref. [52]. Copyright 2006, American Chemical Society

Substitution of methyl-modified precursors by ones with longer alkyl chains favors formation of larger cavities, positively influencing the sensitivity. For the sol-gels of the type (C_*n*_H_2*n*+1_)-Si-(OR)_3_ (R = Et or Me, *n* = 1–12), the sensitivity and oxygen diffusion coefficient increase up to a chain length of *n* = 8 (MTMOS: *K*_*SV*_ ~ 0.02 kPa^−1^, *k*_*q*_ ~ 4 Pa^−1^ s^−1^, *D*_O2_ ~ 2.5·10^7^ cm^2^s^−1^, Octyl-triMOS: *K*_*SV*_ ~ 0.1 kPa^−1^, *k*_*q*_ ~ 22 Pa^−1^ s^−1^, *D*_O2_ ~ 20·10^7^ cm^2^s^−1^) after which point they decrease again (Fig. 4) [52]. The increase in *D*_O2_ due to porosity is higher than the decrease *S*_O2_ in the hydrophobic matrix; however at an *n* > 8, the long alkyl chains obstruct oxygen diffusion. The optimal chemical composition favoring sensors with the highest sensitivity (*K*_*SV*_ 0.2 kPa^−1^, *k*_*q*_ 30 Pa^−1^ s^−1^) and linearity of the Stern–Volmer plot from 0 to 100% O_2_ was reported to be 60 mol % of Octyl-triEOS and 40 mol% of TEOS [53].

Similarly, Yeh et al. found linear SV plots in Octyl-triEOS/TEOS sol-gels doped with platinum(II) porphyrin which were characterized by medium sensitivities (*k*_*q*_ ~ 5 Pa^−1^ s^−1^) but rather fast response times (0.7 s response, 14 s recovery) [54].

Making use of fluorinated alkyl substituents in Ormosils represents another interesting possibility for tuning oxygen-sensing capabilities since (partially) fluorinated polymers are generally known to have significantly higher oxygen permeability compared to their non-fluorinated analogues [55–58]. As was shown, sol–gel materials prepared from the mixture of trifluoropropyltrimethoxysilane TFP-triMOS and non-fluorinated precursors methyl-triEOS, ethyl-triEOS, propyl-triEOS, and phenyl-triEOS (1:1 molar ratio) demonstrate significantly higher sensitivities (3–fivefold) compared to the Ormosils that do not contain TFP-triMOS [59, 60]. For example, the *k*_*q*_ values were 10 and 35 Pa^−1^ s^−1^ for methyl-triEOS and TFP-triMOS/methyl-triEOS, respectively; 10 and 50 Pa^−1^ s^−1^ for ethyl-triEOS and TFP-triMOS/methyl-triEOS, respectively; 20 and 50 Pa^−1^ s^−1^ for propyl-triEOS and TFP-triMOS/propyl-triEOS, respectively; and 5 and 20 Pa^−1^ s^−1^ for phenyl-triEOS and TFP-triMOS/phenyl-triEOS, respectively. Evidently, the Ormosils containing phenyl groups show both the lowest sensitivity and the slowest response times (0.5 s for methyl-triEOS to propyl-triEOS, 2.7 s for phenyl-triEOS) [59]. The SV plots can be adequately fitted with SV equation although they show slight deviation from linear behavior. From the available data, however, it is not certain if the observed increase in sensitivity in case of fluorinated alkyl substituents is only due to higher O_2_ solubility in fluorinated xerogels or if other factors such as porosity contribute as well.

The TFP-triMOS precursor was also employed by other groups to produce sensor materials with high sensitivity and linear SV plot [61–64]. However, in some of these reports, the oxygen concentration was increased in a single step from 0 to 20% O_2_ [61, 63, 65] and without intermediate data points, it is therefore possible that the SV plots deviated from linear behavior in the low O_2_ concentrations and a more sensitive part of the curve were overlooked. The propyl-triMOS/TFP-triMOS (1:1 and 1:2) doped with Ru(dpp)_3_ was reported to reach the *k*_*q*_ values of 50–60 Pa^−1^ s^−1^ and response times < 5 s [61, 62], while doping with PtTFPP and PtOEP, interestingly, only yielded *k*_*q*_ of 8 Pa^−1^ s^−1^ in the same matrix [63] and 10 Pa^−1^ s^−1^ in TFP-triMOS/TEOS/Octyl-triEOS [64]. This observation correlates well with the one noted by Han et al. for mesoporous SBA-15 material [13] but the difference in *R*_*c*_*·p* in the Ormosil matrix (~ fivefold) appears to be slightly higher than in the mesoporous material.

### Aerogels

A subgroup of sol–gel materials are aerogels, sol–gel monoliths that are dried in supercritical CO_2_ to preserve the initial high porosity of the gel (Fig. 2D). This high porosity leads to fast oxygen diffusion within the sensing material, which enhances oxygen sensitivity. For example, TMOS-based aerogels with high surface areas of 870 m^2^g^−1^ doped with fluorescent *N*-(3-trimethoxysilylpropyl)-2,7-diazapyrenium bromide (DAP) were characterized by the bimolecular quenching constant *k*_*q*_ of 330 Pa^−1^ s^−1^ despite a rather small *K*_*SV*_ value of 0.005 kPa^−1^ explained by short decay time of the indicator [66]. Much higher sensitivity (*K*_*SV*_ 2.5 kPa^−1^, *k*_*q*_ 1020 Pa^−1^ s^−1^) was reported for the more sensitive region of the nonlinear SV plot of the more porous aerogel doped with Ru(II)-tris(1,10-phenanthroline)-containing electron-acceptor dyads [66, 67]. However, as the calibration of the DAP-containing aerogel [66] was done with only two calibration points, at 0 and 100% O_2_, respectively, a more sensitive part might easily have been overlooked. Even higher sensitivity (*K*_*SV*_ 14 kPa^−1^, *k*_*q*_ 2800 Pa^−1^ s^−1^ for 0–5% O_2_ range) was reported by Plata et al. for aerogel prepared from TMOS and doped with Ru(dpp)_3_ [68]. These values are about 2 orders of magnitude higher than the quenching constant obtained for Ormosils based on similar composition and indicators. The response times are difficult to determine reliably; the measured values appear to be strongly overestimated. The measured response times for the aerogel monolith (0.5 × 0.5 × 1 cm^3^) were < 8 s [66, 67]. Plata et al. had similar limitations as the gas flow was adjusted manually [68] so that the reported response times of < 10 s are also likely to be strongly overestimated.

While aerogels provide fast gas diffusion and unmatched O_2_ sensitivities, a massive drawback is the lack of versatility and mechanical stability. Aerogels are usually prepared as monoliths in much larger dimensions than commonly used oxygen sensors and therefore are difficult/impossible to apply in the most common sensor formats including thin films, fiber-optic sensors, or nanoparticles. Additionally, aerogels are not very mechanically stable and tend to collapse upon contact with water, which permanently destroys the high porosity. Another drawback is the cumbersome preparation, as drying in supercritical CO_2_ requires adequate equipment and increases cost and effort of preparation while comparably long drying and aging times of several days present another inconvenience.

## Electrospun fibers

Electrospinning polymer solutions in organic solvents produce mats of thin polymer fibers with high surface-to-volume ratio and high free volume. Due to fast gas diffusion, these materials have significantly reduced response times while retaining the favorable mechanical properties of polymers [69–71]. The nature of the host polymer is not different in nanofibers than in films; therefore, a significant improvement in sensitivity is not to be expected compared to conventional sensor formats. Indeed, electrospun nanofibers prepared from polystyrene PS [72–78] showed bimolecular quenching constants similar to those of the bulk PS optodes [79–84] with *k*_*q*_ values in the range of 1–15 Pa^−1^ s^−1^. Ormosil nanofibers prepared from octyl-triEOS/TEOS [85] display a *k*_*q*_ of 5 Pa^−1^ s^−1^, which is exactly in the range expected for these Ormosils [52, 53].

**Fig. 5:**
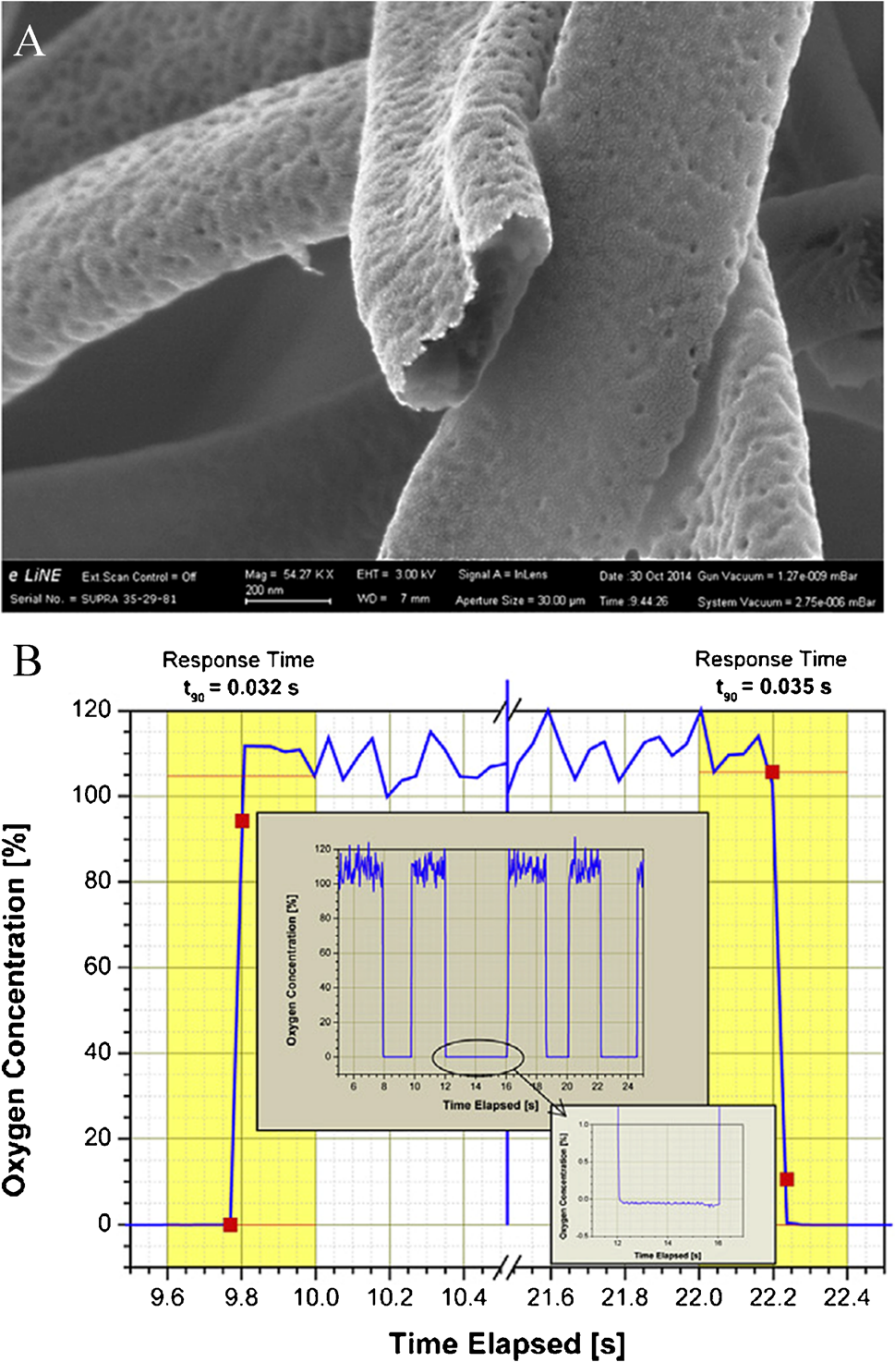
**A** SEM image of the electrospun polystyrene nanofibers showing the hollow structure of the fibers with average diameter of 620 nm and wall thickness of about 100 nm. The scale bar is 200 nm. **B** Dynamic response of the same nanofibers to fast alteration of gas atmosphere from 100% nitrogen to 100% oxygen and back. Reprinted from Ref. [77], Copyright 2015, with permission from Elsevier

One of the main expected advantages of nanofibers compared to bulk sensing films are very fast response times due to the high porosity of the fiber mats and the short diffusion paths within the material. The very fast response times achieved by electrospun nanofiber sensors have been demonstrated by Wolf et al., who compared the properties of platinum(II) porphyrin PtTFPP immobilized into polystyrene nanofibers (Fig. 5A) and in bulk polymer films [76, 77]. As expected, the oxygen sensitivity was almost identical in both materials (*k*_*q*_ ~ 1 Pa^−1^ s^−1^). In contrast, the response time of the nanofibers (average ∅ 620 nm, average thickness ~ 100 nm) decreased to 32 ms (Fig. 5B) compared to the 30 µm thick bulk films characterized by the response and recovery times of 2.2 and 4 s, respectively. Fast response of 0.1 s was also reported for a single Ormosil fiber (Octyl-triEOS/TEOS) of 900 nm diameter containing Ru(dpp)_3_ while the sensitivity was similar to bulk Ormosil materials (5 Pa^−1^ s^−1^) [85]. Lannutti and co-workers reported nanofibers prepared of biocompatible polymers such as polycaprolactone (PCL), polyethersulfone (PES), polysulfone (PSU), and polydimethylsiloxane (PDMS) and their combination in core–shell nanofibers [86–88]. Whereas the PCL nanofibers demonstrated response and recovery times of 0.9 and 2.0 s, respectively, the response times determined for the bulk films were in the order of minutes [86]. However, while the diameters of the fibers are given (0.5–7 µm), the thickness of the film is not mentioned, complicating quantitative comparison. Other core–shell nanofibers with the indicator immobilized in a PDMS core covered by a PCL shell displayed response times below 1 s [87].

In many cases, experimental limitations (i.e., necessity of very fast gas exchange in the calibration chamber) are likely to lead to very strong overestimation of the determined response times. For instance, response and recovery times of 5–30 s were reported for PS fibers in different works [72–75, 78], which is comparable to the response times measured for several micrometer thick bulk optodes. Similarly, the response for polyacrylate copolymer fibers was reported to be in the range of tens of seconds that is only slightly shorter than for the planar films made of the same material [89].

On the other hand, significant differences between the measured response times may also be due to the structural difference. In fact, for porous hollow fibers with thin walls (Fig. 5A), a much faster response can be expected compared to the dense non-porous fibers. Unfortunately, the publications on such materials often lack SEM images of adequate magnification.

Another important characteristic of oxygen sensors is the linearity of the SV plot. Whereas the linearity of SV plots for the nanofibers and bulk optodes is often very similar and the plots show characteristic downward curvature [70], in some cases, nanofibers provide linear SV plots. For instance, Xue et al. demonstrated improvement in the linearity for the nanofibers with the indicator immobilized in a PDMS core [87]. It was hypothesized that fast solvent evaporation in nanofibers prevents molecular migration/rearrangement within the fibers and therefore no microheterogeneities can be formed. The same effect was observed for the fibers made of fluorinated copolymers [89].

It should be noted that such useful properties as fast response time are balanced by significantly higher effort in the preparation of nanofibers compared to that of the bulk films that are conveniently manufactured by knife coating, spin coating, screen printing etc. Another inconvenience is poor mechanical stability of the nanofiber agglomerate and potential problems with attachment of the fibers to the support material. A possible solution may be immobilization of nanofibers in another polymeric matrix but such approach is only feasible if highly gas permeable matrices are used (such as silicone rubber or amorphous Teflon AF polymers) since oxygen diffusion in this balk matrix is a limiting factor in the overall response.

## MOFs

**Fig. 6:**
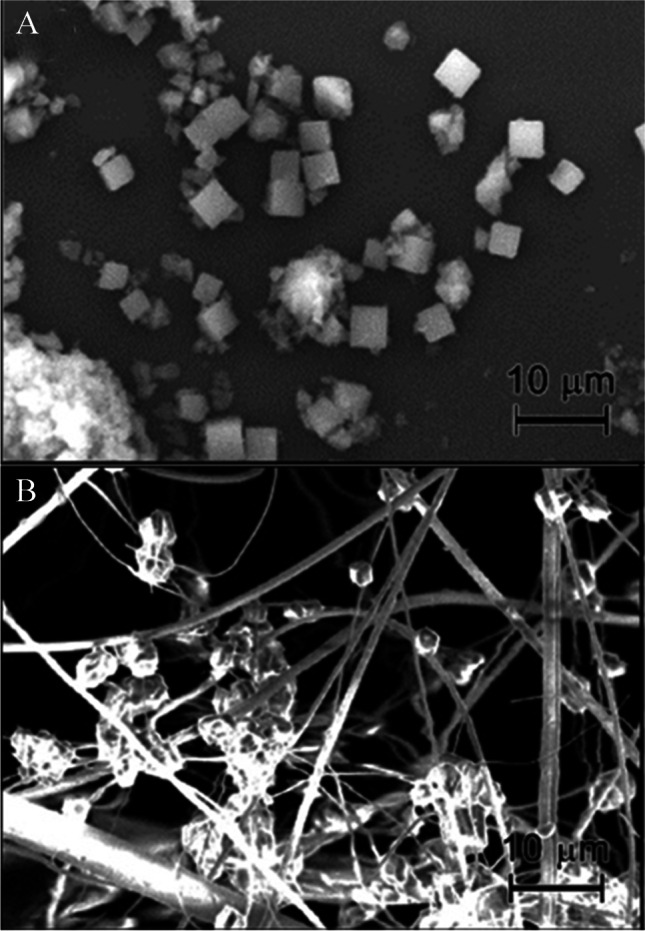
SEM images of PCN-224 MOF in form of free-standing crystals (**A**) and crystals grown on a glass fiber filter (**B**). Republished with permission of the Royal Society of Chemistry from Ref. [97], permission conveyed through creative commons license CC BY 3.0

**Fig. 7:**
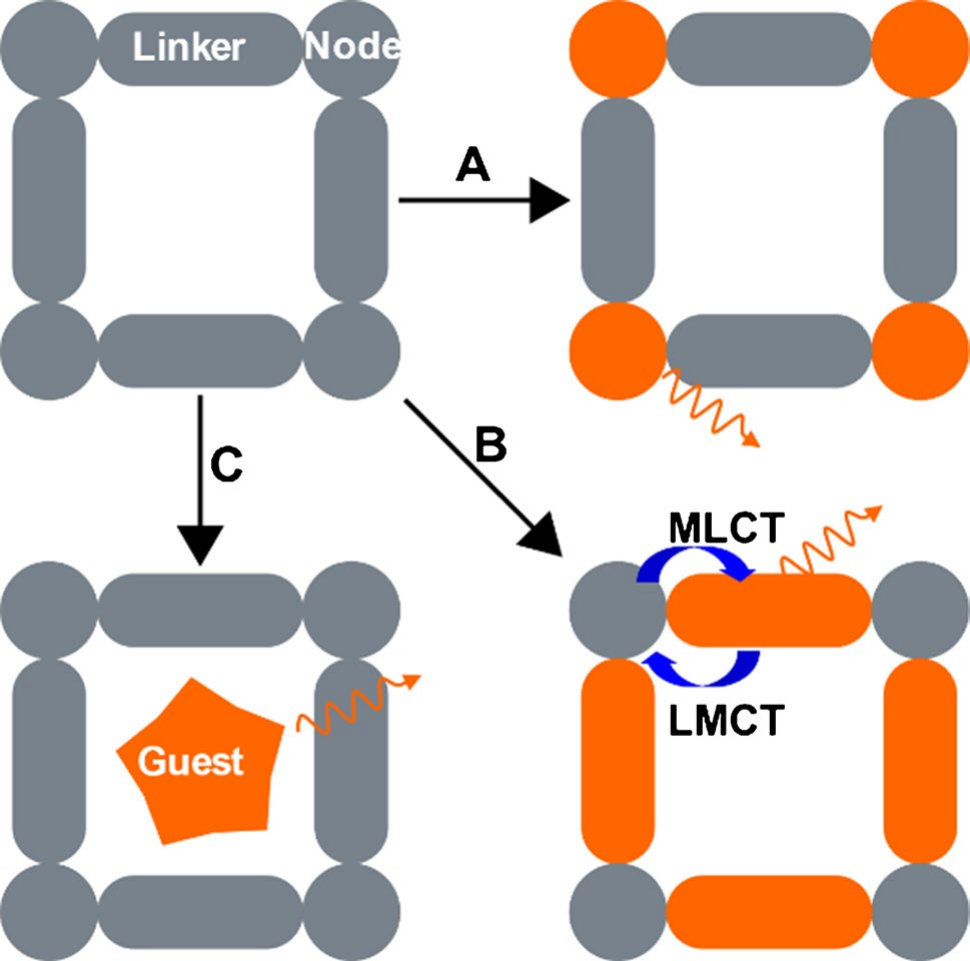
Schematic representation of creating luminescent MOFs by **A** introducing luminescent lanthanide ions as nodes; **B** utilizing luminescent organic molecules as linkers; **C** doping of the MOF with luminescent guest molecules

Metal–organic frameworks are extended crystalline structures (Fig. 6) that contain metal cation-based or metal cation cluster-based nodes that are connected through organic linkers, multitopic aromatic molecules coordinating the nodes via carboxylic acid or amine groups. Due to this modular structure, the chemical and mechanical properties such as polarity or porosity can be tuned over a wide range [90, 91]. In order to utilize MOFs in optical oxygen sensing, the MOF has to be rendered luminescent either during synthesis or via post-synthetic modification or doping (Fig. 7) [92]. The luminescence can arise:

A) From the linkers. A fraction or all linkers in the framework can be luminescent, either on their own (aromatic, π-conjugated structures) or through metal-to-ligand charge transfer (MLCT) or ligand-to-metal charge transfer (LMCT) in MOFs containing d^10^ transition metals such as Zn(II), Cd(II), Cu(I), and Ag(I) [93, 94].
B) From the metal nodes. In this case, lanthanide ions like Eu^3+^ are often responsible for the luminescence, sometimes sensitized by organic chromophores or linkers via an antenna effect [95, 96].
C) From a luminescent guest molecule that has been encapsulated into the MOF structure during synthesis or doped into the structure post-synthesis.

As will be discussed below, MOFs represent materials where even fluorescent dyes such as fluorescent linkers or encapsulated fluorescent probes display significant oxygen sensitivity due to extremely fast oxygen diffusion and even distribution of dye molecules. The MOFs where oxygen sensitivity is primary due to linkers [97–104] or encapsulated guest molecules [105–109] may show *k*_*q*_ values up to 37,000 Pa^−1^ s^−1^. In contrast, MOFs utilizing luminescent nodes [96, 103, 110–113] in general appear to have significantly smaller bimolecular quenching constants (*k*_*q*_ of 0.01–720 Pa^−1^ s^−1^). This may be due to lower oxygen accessibility of the much smaller metal nodes in comparison to the often bigger and less sterically shielded linkers and guest molecules. Additionally, in case of lanthanide nodes (such as Eu(III)), the luminescence quenching by oxygen is expected to be fairly inefficient due to rather weak interaction of the partly filled shielded f-orbitals with the quencher.

Common oxygen indicators such as Ru(II) and Ir(III) polypyridyls can be incorporated in MOFs particularly if equipped with carboxylic groups for coordination to the nodes. The complexes can be embedded as guests (Fig. 7C) or perform the function of a linker (Fig. 7B). For example, carboxylated bipyridine-, phenylpyridine-, or phenanthroline-derived MOF linkers served as one of the three ligands in Ir(III) and Ru(II) polypyridine complexes that were built inside the framework [98–102]. Using an oxygen indicator as linker is advantageous for several reasons. First, the concentration of indicator can be quite high, leading to high signal intensity, while aggregation is prevented by the discrete spatial distribution of the indicator molecules inside the framework. Second, due to high cross-section of the indicator molecules compared to the relatively small nodes and favored oxygen diffusion through the channels of the MOF, high sensitivity of linker-based oxygen sensitive MOFs can be expected.

Advantages and limitations of oxygen sensitive MOFs based on linker luminescence can be illustrated by PCN-224 type family. Meso-tetra-(4-carboxyphenyl)porphyrin (TCP) is a well-known building block for several MOFs such as PCN-224 which is known to have a permanent porosity of 2600 m^2^g^−1^ and large average pore diameters of 19 Å [114]. Burger et al. demonstrated the extreme quenching capabilities of molecular oxygen in the PCN-224 family of MOFs that were built by the metal-free porphyrin as well as Pt(II) and Pd(II) complexes (Pt-PCN-224 and Pd-PCN-224, respectively) [97]. Particularly, even in case of PCN-224, the fluorescence was quenched rather efficiently (*Ksv* 0.25 kPa^−1^, *k*_*q*_ = 37,000 Pa^−1^ s^−1^). The phosphorescent Pt-PCN-224 and Pd-PCN-224 displayed the *k*_*q*_ value around 3900 and 6700 Pa^−1^ s^−1^, respectively, which correlates well with the value obtained for PCN-224 considering the contribution of the spin-statistical factor. Importantly, the luminescence decay times of Pt-PCN-224 and Pd-PCN-224 are in microsecond time domain that is responsible to extremely high sensitivities (*K*_*SV*_ of 73 and 2610 kPa^−1^, respectively) and corresponding very low detection limits (1 Pa and 0.015 Pa, respectively).

Similar to inorganic silica materials, MOFs are usually obtained in the form of microscopic crystals that require immobilization to become useful sensing materials. Again, similarly to silica materials, polymer components such as crosslinkers might intrude into the MOF’s pores and reduce sensitivity. Depending on the MOF structure, this loss in sensitivity can be moderate (ca. threefold reduction in sensitivity in MAF-2, MAF-4, and a Eu-NDC MOF) [105, 111, 113] or almost complete as observed in some PCN-224 type MOFs [97]. The magnitude of this effect appears to depend on the size of the apertures connecting the pores of the framework. In fact, the apertures are rather small in MAF-2 and MAF-4 (1.5–3.6 Å and ~ 3.3 Å, respectively), whereas the pore diameter of PCN-224 reaches 19 Å. It is therefore likely that small apertures prevent intrusion of polymer/prepolymers/cross-linkers into the pores so that the sensitivity is less affected by immobilization. One of potential solutions in case of the MOFs with large pores is to grow them on different supports in order to obtain mechanically stable materials. For instance, cross-linked electrospun polyacrylonitrile fibers, silica TLC plates, glass filters (Fig. 6B), and other supports were shown to be suitable [97].

Another serious limitation is cross-talk of the MOF-based sensing materials to humidity. Despite being highly hydrolytically stable on its own, the MOF may show an extreme decrease in the oxygen sensitivity at high humidity and in water, which can only be reversed by regeneration of the material at elevated temperature in vacuum. As shown for Pt-PCN-224, the MOFs in water still show some luminescence quenching by oxygen; however, the *K*_*SV*_ values reduce drastically (73 kPa^−1^ in dry gas and 0.14 kPa^−1^ in water) [97, 115]. Water molecules appear to be efficiently trapped inside the relatively polar MOF and thus hinder diffusion of oxygen through the pores Although such materials may be useful for (intracellular) sensing dissolved oxygen when used in form of nanoparticles, the oxygen sensing capabilities are generally inferior to conventional nanosensors based on indicators embedded into polymeric or sol–gel nanoparticles [115, 116]. Whereas only a few building blocks can be used to obtain oxygen-sensitive MOFs, the conventional materials are virtually unlimited in terms of combination of the indicators with desired spectral properties and the matrix. A possible way to overcome or reduce humidity interference is to increase hydrophobicity of the MOF for instance using a fluorinated porphyrin linker or additional fluorinated coordinating ligands for the nodes.

## Other nanostructured materials for optical oxygen sensing

### Polymers

**Fig. 8:**
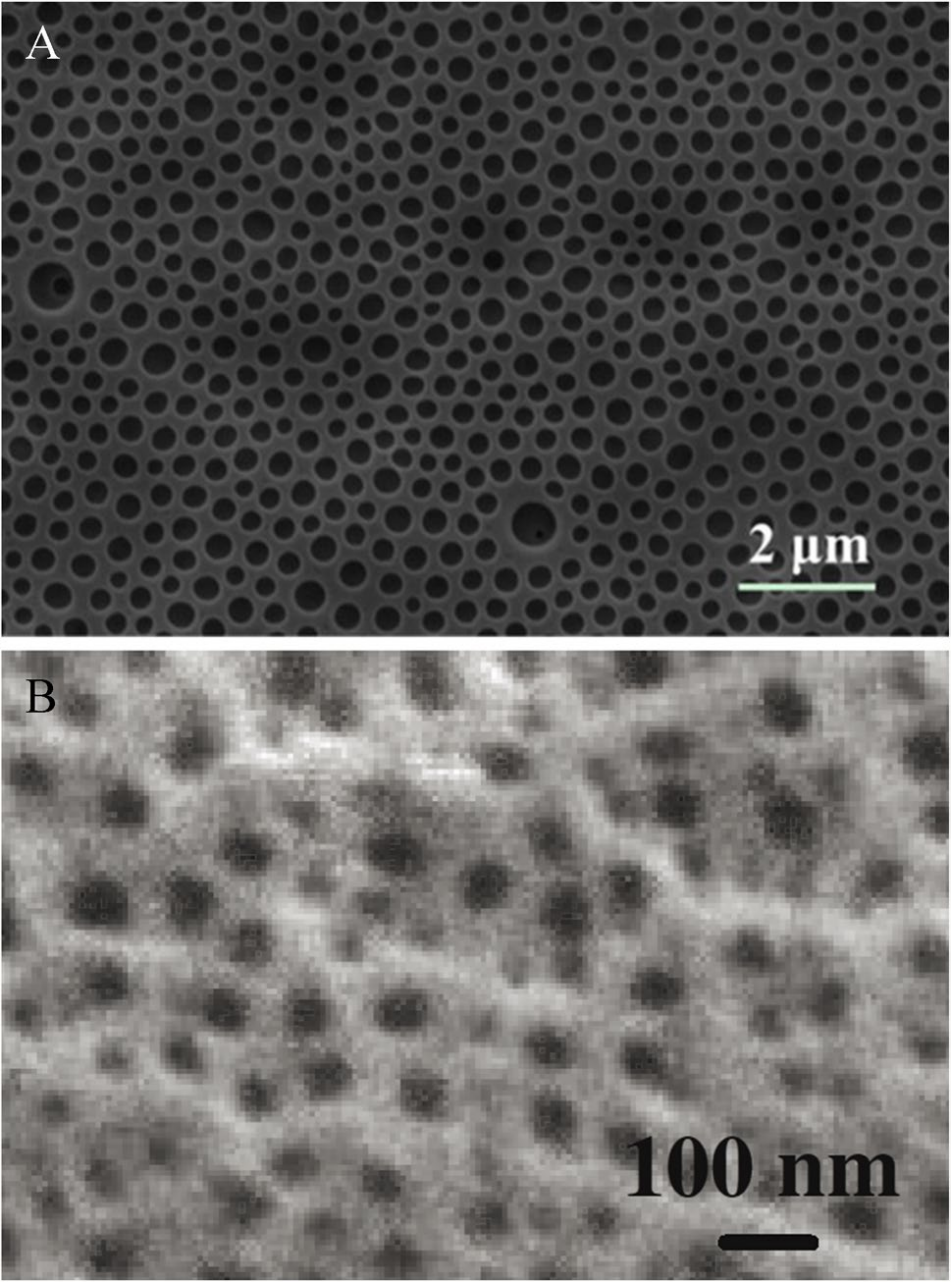
**A** SEM image of the porous film prepared by “breath figure” method from a copolymer of styrene and 4-vinylpyridine. Reprinted from Ref. [118], Copyright 2018, with permission from Elsevier; **B** SEM image of anodized aluminum surface. Reprinted from Ref. [123] © IOP Publishing. Reproduced with permission. All rights reserved

Several strategies of increasing the porosity/surface area of polymers have been utilized in order to enhance oxygen sensitivity. XAD-4, a commercially available cross-linked polystyrene that is characterized by pore size of ~ 100 Å and a surface area of ca. 750 m^2^g^−1^, was stained with oxygen indicators to give sensors with *k*_*q*_ values ranging from 170 [117] to 10 Pa^−1^ s^−1^ [29] which is significantly higher than for bulk polystyrene sensors. In a different approach, nanopores of 200 nm diameter were generated in the surface of the copolymer of polystyrene and 4-vinylpyridine via the “breath figure method” (Fig. 8A) [118]. In this method, the partly amphiphilic polymer is dissolved in water miscible solvent like THF and coated onto a substrate. This film is then subjected to a humid environment prior to solvent evaporation, leading to formation of micelles, stabilizing water droplets, that leave behind pores after solvent evaporation. The authors reported 1.5-fold increase in the oxygen sensitivity with the increase in the pore density accompanied by the ~ 1.6-fold decrease of the response time (see Table S7), the improvements that appear not very significant compared to the potential drawbacks associated with higher hydrophilicity of the used polymer. The same group also created PDMS micropillar arrays with micropillar diameter of ~ 50 µm and covalently immobilized the indicator on their surface [119]. The increase in sensitivity was almost one order of magnitude compared to the solid sensing film, and the response times were dependent on the flow rate and therefore were very likely highly overestimated. Papkovsky and co-workers reported oxygen sensors based on commercially available polypropylene PP and polyethylene PE microfibers [120]. With thicknesses of 40 µm, they are significantly thicker than electrospun fibers and in contrast to the latter, the dye is doped into the fibers via tensile drawing and solvent crazing instead of co-dissolving the dye and polymer in one “cocktail.” The *k*_*q*_ values for the hollow PP and solid PE fibers were 10 and 13 Pa^−1^ s^−1^, respectively. The response times of 60 and 30 s, respectively, are comparable to those of non-porous bulk sensing materials that leads to the conclusion that on this scale of thicknesses, the introduced porosity does not result in improvement of the response times.

Polymers of intrinsic microporosity (PIMs) represent another group of materials that are potentially suitable for design of highly sensitive optical oxygen sensors [121]. Because of the inefficient packing of macromolecular chains, these materials feature a network of voids with dimensions below 2 nm and thus are characterized by very high permeability for gases including oxygen. Another advantage is processability directly from solution in organic solvents. On the other hand, PIMs show strong aging over time associated with the significant decrease of the gas permeability that makes them less suitable for preparation of high-performance stable oxygen sensing materials [122].

### Anodized alumina and aluminum oxide materials

Anodized alumina is another highly porous material that was used to prepare oxygen sensors (Fig. 8B). It is produced via anodic oxidation of aluminum awarding homogenous pore size and distribution that can be tuned via the parameters of the electrochemical process. This high surface area is exploited to prepare highly sensitive oxygen sensors from anodized alumina. Immobilization of different oxygen indicators on anodized alumina (pore diameters of 20–100 nm) via adsorption delivered very interesting results: the dyes featuring longer excited state lifetimes showed significantly lower *k*_*q*_ value (220 and 160 Pa^−1^ s^−1^ for Ru(dpp)_3_ and PtTCPP, respectively) than the fluorescent dyes (12,400 and 4300 Pa^−1^ s^−1^ for H_2_TCPP and PBA, respectively) [123] that only partly can be explained by the contribution of the spin-statistical factor. Except for PtTCPP, the response times were in the submillisecond range with H_2_TCPP displaying the fastest response of only 10 µs. The response time was observed to be dependent on the thickness of the aluminum layer, and an effective diffusion coefficient of 5·10^−6^ m^2^s^−1^ was estimated for the anodized alumina used in these experiments. By anchoring porphyrins on the surface of anodized aluminum with average pore diameter of 12–20 nm, Araki et al. achieved similarly high sensitivity [124]. Again, the metal free porphyrins featured about two orders of magnitude higher *k*_*q*_ than the corresponding platinum(II) complexes (14,000–23,000 vs. 120–210 Pa^−1^ s^−1^). The SV plots were nonlinear with the platinum porphyrins showing stronger deviation from linearity than the metal free porphyrins. The response times were in the range of several seconds which is likely due to use of a setup based on gas flow. This experimental limitation can be addressed by performing pressure jump experiments [125] in which pressure is increased abruptly from near vacuum to ambient pressure in a specially designed pressure chamber. Whereas the silica-gel manually applied to an aluminum plate and commercially available TLC support materials yielded very fast response times of around 10 ms and 25 µs, respectively, the response of the anodized alumina sensors was much slower (0.4 s). This is likely due to the silicone layer on the porous surface that was used for dye immobilization.

Despite potential usefulness of anodized alumina for some applications, this material is limited by lack of versatility due to planar format of the sensor and absence of light transparency that makes the read-out from the dye-modified side the only possibility. Additionally, immobilization of indicators either has to be conducted via coating with a polymer-dye “cocktail” that fills the pores of the anodized material and therefore increases the response time, or adsorption of the dye on the surface. This second method likely retains fast response times but leaves the sensor material vulnerable to leaching of the indicator.

Other aluminum-based substrates used include TLC plates coated with aluminum oxide. Similar to silica-gel TLC plates, the indicator dyes are adsorbed on the porous substrate, guaranteeing fast gas diffusion and high sensitivity while providing some mechanical stability. Amao et al. discussed the oxygen sensing performance of various indicators (mostly metalloporphyrins and a terbium-complex) that was deposited on alumina films [126–128]. Once again, the highest *k*_*q*_ value was achieved for the dye with the shortest lifetime, H_2_TCPP (35,300 Pa^−1^ s^−1^). Phosphorescent indicators, though giving higher *K*_*SV*_ values (1.5–4.5 kPa^−1^ vs. 0.4 kPa^−1^ for fluorescent H_2_TCPP) due to much longer decay times, yielded *k*_*q*_ values of several orders of magnitude smaller (3–20 Pa^−1^ s^−1^). The reason for such very different behavior of fluorescent and phosphorescent indicators on alumina plates and anodized aluminum is not quite clear. Different aggregation behavior of dyes upon immobilization might be one of the possibilities. This possibility was suggested by Fernández-Sánchez and co-workers who investigated different indicators and aluminum oxide samples of a variety of morphologies [129]. The sensitivity was found to increase with pore volume with optimal material having an average pore diameter of 192 Å and total pore volume of 20 mL m^−2^. Depending on the indicator (Ir(III) cyclometalated complexes), the *k*_*q*_ values ranged from 140–510 [129] to ~ 14,000 Pa^−1^ s^−1^ for one of the indicators [130], which is orders of magnitude higher than for the same dyes immobilized in polystyrene (2–6 Pa^−1^ s^−1^). The reason for such drastic difference between the indicators is likely to be in their aggregation behavior on the porous surface of aluminum oxide.

## Comparison of different porous and non-porous matrices

Table 1 summarizes the properties of the most important classes of porous materials used for oxygen sensing and Tables S2–S9 provide a more detailed summary of individual materials. Although the dynamic response time is an important parameter for comparison, its reliable determination is often challenging and the literature values should be taken with care. This is especially true for porous materials many of which are expected to show very fast response (response times < 1 s). It is often noted by the authors themselves that the measured dynamic response times are likely to represent the time needed for gas exchange in the measurement chamber.

**Table 1:** Properties of the most important classes of porous materials used in optical oxygen sensors and of selected representatives of sensors based on non-porous matrices

Material type	Typical porosity	Typical *k*_*q*_, Pa^−1^ s^−1^	Advantages	Limitations
Silica-gel beads	350–500 m^2^g^−1^, 60–85 Å	40–200	Commercially available; simple immobilization procedures	Cross-talk to humidity; the particles have to be dispersed in a matrix material that may affect sensing properties
Mesoporous silica	550–960 m^2^g^−1^, 30–90 Å	30–3500	Hierarchical porous structure; tuneable pore size; some commercially available	The particles have to be dispersed in a matrix material. Cross-talk to humidity is expected
Silica aerogels	~ 1000 m^2^g^−1^	1000–3000	High sensitivity	Very poor mechanical stability; only free-standing films possible; collapse upon pressure application and when in contact with water
Sol-gels (silica- and Ormosil-based)	n.d.	3–120 Up to 2300^(a)^	Sensitivity of Ormosils can be tuned by varying ratio and nature of organic substituent	Bulk layers are subject to cracking; long aging that affects the sensing characteristics
MOFs	400–3000 m^2^g^−1^, 3–25 Å	Up to 7000^(b)^ Up to 37,000^(c)^	Extreme sensitivity; defined composition and pore structure	Immobilization of crystals may strongly reduce the sensitivity; strong humidity cross-talk
Electrospun nanofibers	n.d	0.1–130	The same materials as for bulk optodes; sensitivity similar to bulk optodes but very fast response times	Significant time expenditure for preparation; lower mechanical stability; coating of extra layers (optical isolation) is challenging
Anodized alumina	150–1000 Å	14,000–35,000^(d)^	Mechanically robust	Many dyes may aggregate; sensor format limited to planar optodes; inflexible support
Polystyrene	Non-porous	3–5 [136]	Available in a variety of formats: planar optodes, fiber-optic sensors, and nanoparticles showing similar sensing properties; applicable for measurements in aqueous solutions; simple manufacturing; commercially available	Moderate sensitivity unless dyes with very long decay times (ms time domain) are used
Perfluorinated polymers (Teflon AF, Hyflon AD)	Non-porous	30–80 [58, 135]	High oxygen permeability of the matrices and thus the sensitivity; applicable for measurements in aqueous solutions; simple manufacturing; polymers are robust towards oxidation	Polymers are expensive; poor compatibility of the existing indicators with the matrices leading to aggregation; chemical modification of the dyes challenging
Silicone rubber	Non-porous	2–30 [28, 29, 119, 134]	High oxygen permeability and thus the sensitivity; low cost; applicable for measurements in aqueous solutions; simple manufacturing	Most indicators aggregate; have to be chemically modified to achieve better compatibility or covalently immobilized

^(a)^High sensitivities only measured for erythrosine B[31, 137]

^(b)^For phosphorescent MOFs

^(c)^For fluorescent MOFs

^(d)^For fluorescent dyes

In terms of oxygen sensitivity, the highest values are achieved with metal–organic frameworks and dyes supported on porous anodized alumina (Table 1, Tables S7–S8). The MOFs offer advantage of very defined structure and virtually unlimited combination of the building blocks. Additionally, the aggregation of the indicators can be efficiently prevented if they represent structural elements of the MOFs. On the other hand, the number of MOFs suitable for oxygen sensing is still quite limited. Some MOFs that rely on fluorescent dyes as building blocks feature extremely high bimolecular quenching constants up to 37,000 Pa^−1^ s^−1^ but nevertheless have moderate sensitivity due to short fluorescence decay time of several nanoseconds only. On the other hand, utilization of phosphorescent building blocks based on Pt(II) and Pd(II) tetracarboxyporphyrins provides an ultimate sensitivity (*K*_*SV*_ up to 2610 kPa^−1^, LODs as low as 0.015 Pa) but the structural variety of such MOFs is limited (PCN224, PCN222, PCN225, MOF525, and the like). Non-covalent entrapment of existing oxygen indicators into MOFs is of course possible, but bears the risk of dye aggregation and inhomogeneous distribution.

Dyes adsorbed on porous anodized alumina also have been reported to show extremely high bimolecular quenching constants (Table 1). Here, the risk of dye aggregation is particularly high, so only some of oxygen indicators are likely to be suitable. In many cases, immobilized fluorescent indicators showed several orders of magnitude higher *k*_*q*_ values compared to the phosphorescent analogues which cannot be explained only by the contribution of the spin-statistical factor in luminescence quenching.

Silica-based porous materials (silica-gels, mesoporous silica, sol-gels including Ormosils) are characterized by relatively high *k*_*q*_ values (Table 1) which however vary over a great range (1–2 orders of magnitude) within each group. This may be due to significant differences in the structure of individual materials, immobilization method (via electrostatic interactions or covalent bonding), the extent of dye aggregation, and other factors. For instance, the sensitivity of Ormosils sol-gels not only depends on the nature and length of the organic substituent (Fig. 4) [52] but also on preparation method [12, 51] which may be explained by different speed of hydrolysis of precursors and thus resulting porosity. In certain cases, even for matrices of the same composition and dye type (triplet emitters), the difference in *k*_*q*_ value can be very strong, up to 1 order of magnitude [63]. Compared to silica-based sol-gels, the respective aerogels are generally more sensitive, but they suffer from poor mechanical stability and irreversible collapse when in contact with water, and thus are rather of academic interest.

Electrospun nanofibers and polymers with nanopores in the polymer surface occupy intermediate position between porous matrices and optodes based on bulk layers. Whereas the sensitivity is generally similar to the bulk materials, the dynamic response times can improve by several orders of magnitude [70]. The effect is similar to that observed for fiber-optic microsensors, where a thin layer of the sensing material is coated onto the optical fiber tip of small diameter (typically 20–100 µm). Additionally, in some cases, the gas transport can be additionally favored by formation of hollow fibers with thin porous walls [76, 77]. On the other hand, preparation of electrospun nanofibers is significantly less straightforward compared to the bulk optodes and the mechanical stability of the mats may be poor.

Apart from sensitivity and response times, another important aspect to be considered is the handling of the respective material and the suitability for application as planar optodes and fiber-optic sensors. For instance, the anodized alumina support is not transparent to light and is rigid, which limits potential applications to imaging of oxygen distribution on planar surfaces. Free-standing MOF crystals cannot be applied easily and serve only as a model system, and MOF immobilization in polymers might be challenging since sensitivity may reduce significantly [97]. Fortunately, it is possible to grow crystals of such MOFs on different porous supports [97] whereas other MOFs with smaller pores appear not to show drastic sensitivity decrease upon immobilization [111]. Handling of sensors based on electrospun fibers also may be challenging for many applications like imaging of oxygen distribution on surfaces. However, they may be a nice tool for gas measurement if used in a form of an agglomerate fixed between the distal end of an optical fiber and a porous filter that would provide the necessary mechanical protection. Finally, silica-gel, mesoporous silica, or sol-gels can be conveniently obtained in form of micrometer-sized particles during synthesis or post-synthetic grinding of the monoliths. These particles can be then easily dispersed in a highly gas-permeable polymer layers, e.g., made of silicone rubber. Sol–gel layers can also be coated on transparent support (e.g., glass) and used similarly to polymer-based bulk optodes but their tendency to age and crack with time should be kept in mind.

Although this review is focused on porous materials for gas sensing applications, their potential suitability for measurement in aqueous solutions should also be considered. Whereas some of the porous materials are only suitable for sensing in gas phase, others can cover a broader application range. For instance, aerogels are reported to collapse in contact with water and oxygen sensitivity of many MOFs was demonstrated to greatly decrease in water and even humid atmosphere [97]. Although silica-gels do not show such drastic effects, their cross-talk to humidity is well documented but can be minimized significantly by modification of the surface with hydrophobic silanes [32]. Silica-based materials generally can be considered suitable for preparation of sensors for measurements in aqueous solutions [131]. Moreover, the inorganic character of the backbone and cross-links makes them insoluble in organic solvents that may be useful for designing sensors for measurements in non-aqueous media providing that the indicator cannot migrate. The same is likely to be true for MOFs that incorporate oxygen indicators as structural elements but also here the potential applicability for measurement in organic solvents has yet to be demonstrated.

It is interesting to compare the properties of oxygen sensors based on porous materials and those based on non-porous polymers into which the oxygen indicator is immobilized (via physical entrapment or covalent coupling). Most conventional materials are prepared in a straightforward manner by coating the solution of a dye and a polymer on a transparent support (planar glass or polymeric substrate, tip of an optical fiber). They also can be manufactured in form of micro- or nanoparticles and used for measurement of oxygen concentration on the microscale. Many of these materials make use of polystyrene or poly(methyl methacrylate) which are inexpensive and possess good mechanical and optical properties. When measuring at ambient conditions, the optimal dynamics (10–400 hPa O_2_) is achieved with indicators having decay times in the range of tens of microseconds, platinum(II) porphyrins being most popular. Higher sensitivity can be reached with dyes that possess decay times in order of several hundred microseconds (Pd(II) porphyrins) but trace sensors can only be manufactured by using indicators with significantly longer decay times (milliseconds) where the choice is very limited and no commercially available indicators are available [2, 7]. Moreover, utilization of indicators with ultra-long decay times is associated with several undesired phenomena making practical applications significantly more challenging [132].

In contrast to polystyrene, polymers possessing very high oxygen permeability can be used to design trace sensors based on readily available Pt(II) and Pd(II) porphyrins. However, their choice is limited to a few polymers including poly(trimethylsilyl propine), silicone rubber, and amorphous perfluorinated polymers such as Teflon AF and Hyflon AD. Main limitations include poor long-term stability for poly(trimethylsilyl propyne) [133] and poor compatibility of existing indicators with silicone rubber and perfluorinated polymers that makes it necessary to synthesize indicator derivatives equipped with respective functional groups for better compatibility [134, 135]. Even though the resulting materials show excellent performance and stability, the sensitivity is still limited and well below the one potentially achievable via similar oxygen indicators with porous materials (Table 1).

## Conclusion

Porous materials are characterized by great structural variety and number of attractive features and for this reason have been widely used to design optical oxygen sensors. High porosity ensures fast oxygen diffusion and thus excellent sensitivities. Whereas this feature is extremely valuable for design of trace and ultra-trace sensors for variety of industrial and environmental applications, it can be a drawback when the “normal range” sensors for most common applications (e.g., in biology, biotechnology, or medicine) are considered. In this case, combination of most common and easily accessible (either commercially or synthetically) luminescent indicators with porous materials results in sensors that are by far too sensitive to ensure acceptable signal to noise ratio at air saturated conditions. For these applications, non-porous polymers like polystyrene are definitely more convenient and versatile to use since they enable manufacturing of sensors in a variety of formats, ranging from planar foils and fiber-optic sensors to (cell-penetrating) water-dispersible nanoparticles. In this respect, electrospun nanofibers represent a very interesting group of materials since sensitivity is comparable or even identical to the bulk sensors based on the same matrices, but an ultra-fast response in order of milliseconds can be achieved.

Porous materials along with highly oxygen-permeable amorphous polymers are matrices of choice for preparation of trace oxygen sensors. Whereas the second group is limited to only a few accessible representatives, porous materials represent a much broader class offering virtually unlimited combination possibilities. Alone in the group of MOFs, an enormous progress has been achieved in the last decade. Since main applications of MOFs are guided by their excellent gas-transport properties, it is likely that many interesting candidates for oxygen sensing either have been already reported in a different context or will appear in future. Although porous materials often have their limitations (cross-talk to humidity, availability in particle form that requires utilization of a second matrix), their potential for gas sensing applications has not been fully discovered yet. For instance, many of them possess a robust 3D network with high ratio of an inorganic component (silica-based materials, MOFs) and thus may be nicely suitable for designing oxygen sensors for non-aqueous environments. Particularly the class of MOFs that is characterized by virtually unlimited structural design possibilities in future may give many interesting and promising materials for sensing oxygen and other gaseous species.

## Supplementary Information

Below is the link to the electronic supplementary material.

Supplementary file1 The ESI contains an overview of abbreviations, information on commercial availability of the discussed sensor matrices and detailed data on matrix materials used in optical oxygen gas sensors and their properties (porosity, response time, KSV, kq and linearity of SV plot). (PDF 639 KB)

## References

[1] DeGraff BA, Demas JN. Luminescence-based oxygen sensors. In: Geddes CD, Lakowicz JR, editors. Reviews in fluorescence 2005, vol. 2005, Boston, MA: Springer US; 2005, p. 125–51. 10.1007/0-387-23690-2_6.

[2] Wang X, Wolfbeis OS (2014). Optical methods for sensing and imaging oxygen: materials, spectroscopies and applications. Chem Soc Rev.

[3] Moßhammer M, Strobl M, Kühl M, Klimant I, Borisov SM, Koren K (2016). Design and application of an optical sensor for simultaneous imaging of pH and dissolved O2 with low cross-talk. ACS Sensors.

[4] Koop-Jakobsen K, Mueller P, Meier RJ, Liebsch G, Jensen K. Plant-sediment interactions in salt marshes—an optode imaging study of O2, pH, and CO2 gradients in the rhizosphere. Frontiers in Plant Science 2018;9. 10.3389/fpls.2018.00541. 10.3389/fpls.2018.00541 PMC5943611 29774037

[5] Nielsen SD, Paegle I, Borisov SM, Kjeldsen KU, Røy H, Skibsted J (2019). Optical sensing of pH and O2 in the evaluation of bioactive self-healing cement. ACS Omega.

[6] Wolfbeis OS (2015). Luminescent sensing and imaging of oxygen: fierce competition to the Clark electrode. BioEssays.

[7] Quaranta M, Borisov SM, Klimant I (2012). Indicators for optical oxygen sensors. Bioanal Rev.

[8] Schweitzer C, Schmidt R (2003). Physical mechanisms of generation and deactivation of singlet oxygen. Chem Rev.

[9] Borisov SM. CHAPTER 1. Fundamentals of quenched phosphorescence O2 sensing and rational design of sensor materials. In: Papkovsky DB, Dmitriev RI, editors. Detection Science, Cambridge: Royal Society of Chemistry; 2018, p. 1–18. 10.1039/9781788013451-00001.

[10] Valeur B, Wiley InterScience (Online service). Molecular fluorescence: principles and applications. New York: Wiley-VCH; 2001.

[11] Lakowicz JR (2006). Principles of fluorescence spectroscopy.

[12] McDonagh C, Bowe P, Mongey K, MacCraith BD (2002). Characterisation of porosity and sensor response times of sol–gel-derived thin films for oxygen sensor applications. J Non Cryst Solids.

[13] Han B-H, Manners I, Winnik MA (2005). Oxygen sensors based on mesoporous silica particles on layer-by-layer self-assembled films. Chem Mater.

[14] Djurovich PI, Murphy D, Thompson ME, Hernandez B, Gao R, Hunt PL, et al. Cyclometalated iridium and platinum complexes as singlet oxygen photosensitizers: quantum yields, quenching rates and correlation with electronic structures. Dalton Trans 2007:3763. 10.1039/b704595f. 10.1039/b704595f 17712442

[15] Lehrer S. Solute perturbation of protein fluorescence. Quenching of the tryptophyl fluorescence of model compounds and of lysozyme by iodide ion. Biochemistry 1971;10:3254–63. 10.1021/bi00793a015. 10.1021/bi00793a015 5119250

[16] Carraway ER, Demas JN, DeGraff BA (1991). Photophysics and oxygen quenching of transition-metal complexes on fumed silica. Langmuir.

[17] Okada T, Yoshido S, Miura H, Yamakami T, Sakai T, Mishima S (2012). Swellable microsphere of a layered silicate produced by using monodispersed silica particles. J Phys Chem C.

[18] Kruk M, Jaroniec M, Sakamoto Y, Terasaki O, Ryoo R, Ko CH (2000). Determination of pore size and pore wall structure of MCM-41 by using nitrogen adsorption, transmission electron microscopy, and X-ray diffraction. J Phys Chem B.

[19] Koren K, Borisov SM, Klimant I (2012). Stable optical oxygen sensing materials based on click-coupling of fluorinated platinum(II) and palladium(II) porphyrins—a convenient way to eliminate dye migration and leaching. Sens Actuators B Chem.

[20] Yun S, Luo H, Gao Y (2014). Superhydrophobic silica aerogel microspheres from methyltrimethoxysilane: rapid synthesis via ambient pressure drying and excellent absorption properties. RSC Adv.

[21] Kautsky H (1939). Quenching of luminescence by oxygen. Trans Faraday Soc.

[22] Twarowski AJ, Good Lisa. Phosphorescence quenching by molecular oxygen: zinc tetraphenylporphin on solid supports. J Phys Chem 1987;91:5252–7. 10.1021/j100304a024.

[23] Wolfbeis OS, Leiner MJP, Posch HE (1986). A new sensing material for optical oxygen measurement, with the indicator embedded in an aqueous phase. Mikrochim Acta.

[24] Krasnansky R, Koike K, Thomas JK (1990). Gaussian approximation to the unique heterogeneous Langmuir-Hinshelwood type fluorescence quenching at the silica gel gas/solid interface: pyrene and 9,10-diphenylanthracene singlet quenching by oxygen. J Phys Chem.

[25] Hartmann P, Leiner MJP, Lippitsch ME (1995). Response characteristics of luminescent oxygen sensors. Sens Actuators B Chem.

[26] Posch HE, Wolfbeis OS (1988). Optical sensors, 13: fibre-optic humidity sensor based on fluorescence quenching. Sens Actuators.

[27] He H, Fraatz RJ, Leiner MJP, Rehn MM, Tusa JK (1995). Selection of silicone polymer matrix for optical gas sensing. Sens Actuators B Chem.

[28] Klimant I, Belser P, Wolfbeis OS (1994). Novel metal—organic ruthenium(II) diimin complexes for use as longwave excitable luminescent oxygen probes. Talanta.

[29] Mingoarranz et al. FJ, Moreno-Bondi MC, García-Fresnadillo D, de Dios C, Orellana G. Oxygen-sensitive layers for optical fibre devices. Mikrochim Acta 1995;121:107–18. 10.1007/BF01248245.

[30] Koren K, Borisov SM, Saf R, Klimant I (2011). Strongly phosphorescent iridium(III)-porphyrins—new oxygen indicators with tuneable photophysical properties and functionalities. Eur J Inorg Chem.

[31] Badia R, Marta E. Diaz-Garcia, Garcia-Fresnadillo A. A sensitive probe for oxygen sensing in gas mixtures, based on room-temperature phosphorescence quenching. Mikrochim Acta 1995;121:51–61. 10.1007/BF01248240.

[32] Borisov SM, Lehner P, Klimant I (2011). Novel optical trace oxygen sensors based on platinum(II) and palladium(II) complexes with 5,10,15,20-meso-tetrakis-(2,3,4,5,6-pentafluorphenyl)-porphyrin covalently immobilized on silica-gel particles. Anal Chim Acta.

[33] Melnikov PV, Naumova AO, Alexandrovskaya AYu, Zaitsev NK. Optimizing production conditions for a composite optical oxygen sensor using mesoporous SiO2. Nanotechnol Russia 2018;13:602–8. 10.1134/S1995078018060083.

[34] Xu H, Aylott JW, Kopelman R, Miller TJ, Philbert MA (2001). A real-time ratiometric method for the determination of molecular oxygen inside living cells using sol−gel-based spherical optical nanosensors with applications to rat C6 glioma. Anal Chem.

[35] Lei B, Li B, Zhang H, Lu S, Zheng Z, Li W (2006). Mesostructured silica chemically doped with RuII as a superior optical oxygen sensor. Adv Funct Mater.

[36] Lei B, Li B, Zhang H, Zhang L, Li W (2007). Synthesis, characterization, and oxygen sensing properties of functionalized mesoporous SBA-15 and MCM-41 with a covalently linked ruthenium(II) complex. J Phys Chem C.

[37] Wu X, Song L, Li B, Liu Y (2010). Synthesis, characterization, and oxygen sensing properties of Ru(II) complex covalently grafted to mesoporous MCM-41. J Lumin.

[38] Zhang H, Sun Y, Ye K, Zhang P, Wang Y (2005). Oxygen sensing materials based on mesoporous silica MCM-41 and Pt(ii)–porphyrin complexes. J Mater Chem.

[39] Wang B, Liu Y, Li B, Yue S, Li W (2008). Optical oxygen sensing materials based on trinuclear starburst ruthenium(II) complexes assembled in mesoporous silica. J Lumin.

[40] Shi L, Li B (2009). A series of Cu(I) complexes containing 1,10-phenanthroline derivative ligands: synthesis, characterization, photophysical, and oxygen-sensing properties. Eur J Inorg Chem.

[41] Liu Y, Li B, Cong Y, Zhang L, Fan D, Shi L (2011). Optical oxygen sensing materials based on a novel dirhenium(I) complex assembled in mesoporous silica. J Lumin.

[42] Haitao J, Huilin Y, Fan L, Yang L (2012). Fabrication and performances of an optical sensor system constructed by a novel Cu(I) complex embedded on silica matrix. J Lumin.

[43] Wang B, Zhang L, Li B, Li Y, Shi Y, Shi T (2014). Synthesis, characterization, and oxygen sensing properties of functionalized mesoporous silica SBA-15 and MCM-41 with a Pt(II)–porphyrin complex. Sens Actuators B Chem.

[44] Lobnik A, Korent Urek Š, Turel M, Frančič N. Sol-gel based optical chemical sensors. In: Proc. SPIE 8073, Optical Sensors 2011; and Photonic Crystal Fibers V, 80730V. 10.1117/12.886819.

[45] MacCraith BD, McDonagh CM, O’Keeffe G, Keyes ET, Vos JG, O’Kelly B (1993). Fibre optic oxygen sensor based on fluorescence quenching of evanescent-wave excited ruthenium complexes in sol–gel derived porous coatings. Analyst.

[46] McEvoy AK, McDonagh CM, MacCraith BD (1996). Dissolved oxygen sensor based on fluorescence quenching of oxygen-sensitive ruthenium complexes immobilized in sol–gel-derived porous silica coatings. Analyst.

[47] Mcevoy AK, Mcdonagh C, Maccraith BD (1997). Optimisation of sol-gel-derived silica films for optical oxygen sensing. J Sol-Gel Sci Technol.

[48] Lee S-K, Okura I (1997). Porphyrin-doped sol-gel glass as a probe for oxygen sensing. Anal Chim Acta.

[49] Lee S-K, Okura I (1997). Optical sensor for oxygen using a porphyrin-doped sol–gel glass. Analyst.

[50] McDonagh C, MacCraith BD, McEvoy AK (1998). Tailoring of sol−gel films for optical sensing of oxygen in gas and aqueous phase. Anal Chem.

[51] Murtagh MT, Shahriari MR, Krihak M (1998). A study of the effects of organic modification and processing technique on the luminescence quenching behavior of sol−gel oxygen sensors based on a Ru(II) complex. Chem Mater.

[52] Tao Z, Tehan EC, Tang Y, Bright FV (2006). Stable sensors with tunable sensitivities based on class II xerogels. Anal Chem.

[53] Tang Y, Tehan EC, Tao Z, Bright FV (2003). Sol−gel-derived sensor materials that yield linear calibration plots, high sensitivity, and long-term stability. Anal Chem.

[54] Yeh T-S, Chu C-S, Lo Y-L (2006). Highly sensitive optical fiber oxygen sensor using Pt(II) complex embedded in sol–gel matrices. Sens Actuators B Chem.

[55] Amao Y, Asai K, Miyashita T, Okura I (2000). Novel optical oxygen sensing material: platinum porphyrin-fluoropolymer film. Polym Adv Technol.

[56] Amao Y, Miyashita T, Okura I (2000). Optical oxygen sensing based on the luminescence change of metalloporphyrins immobilized in styrene–pentafluorostyrene copolymer film. Analyst.

[57] Amao Y, Ishikawa Y, Okura I (2001). Green luminescent iridium(III) complex immobilized in fluoropolymer film as optical oxygen-sensing material. Anal Chim Acta.

[58] Lehner P, Larndorfer C, Garcia-Robledo E, Larsen M, Borisov SM, Revsbech N-P (2015). LUMOS—a sensitive and reliable optode system for measuring dissolved oxygen in the nanomolar range. PLoS ONE.

[59] Higgins C, Wencel D, Burke CS, MacCraith BD, McDonagh C (2008). Novel hybrid optical sensor materials for in-breath O2 analysis. Analyst.

[60] Estella J, Wencel D, Moore JP, Sourdaine M, McDonagh C (2010). Fabrication and performance evaluation of highly sensitive hybrid sol–gel-derived oxygen sensor films based on a fluorinated precursor. Anal Chim Acta.

[61] Bukowski RM, Ciriminna R, Pagliaro M, Bright FV (2005). High-performance quenchometric oxygen sensors based on fluorinated xerogels doped with [Ru(dpp) _3_ ] ^2+^. Anal Chem.

[62] Bukowski RM, Davenport MD, Titus AH, Bright FV (2006). O2-responsive chemical sensors based on hybrid xerogels that contain fluorinated precursors. Appl Spectrosc.

[63] Chu C-S, Lo Y-L (2007). High-performance fiber-optic oxygen sensors based on fluorinated xerogels doped with Pt(II) complexes. Sens Actuators B Chem.

[64] Chu C-S, Lo Y-L (2011). Highly sensitive and linear calibration optical fiber oxygen sensor based on Pt(II) complex embedded in sol–gel matrix. Sens Actuators B Chem.

[65] Ciriminna R, Pagliaro M (2009). Organofluoro-silica xerogels as high-performance optical oxygen sensors. Analyst.

[66] Leventis N, Elder IA, Rolison DR, Anderson ML, Merzbacher CI. Durable modification of silica aerogel monoliths with fluorescent 2,7-diazapyrenium moieties. Sensing oxygen near the speed of open-air diffusion. Chem Mater 1999;11:2837–45. 10.1021/cm9901966.

[67] Leventis N, Rawashdeh A-MM, Elder IA, Yang J, Dass A, Sotiriou-Leventis C. Synthesis and characterization of Ru(II) Tris(1,10-phenanthroline)-electron acceptor dyads incorporating the 4-benzoyl-*N*-methylpyridinium cation or *N*-benzyl-*N*-methyl viologen. Improving the dynamic range, sensitivity, and response time of sol−gel-based optical oxygen sensors. Chem Mater 2004;16:1493–506. 10.1021/cm034999b.

[68] Plata DL, Briones YJ, Wolfe RL, Carroll MK, Bakrania SD, Mandel SG (2004). Aerogel-platform optical sensors for oxygen gas. J Non Cryst Solids.

[69] Imran M, Motta N, Shafiei M (2018). Electrospun one-dimensional nanostructures: a new horizon for gas sensing materials. Beilstein J Nanotechnol.

[70] Rivero P, Goicoechea J, Arregui F (2018). Optical fiber sensors based on polymeric sensitive coatings. Polymers.

[71] George G, Luo Z (2020). A review on electrospun luminescent nanofibers: photoluminescence characteristics and potential applications. CNANO.

[72] Wang Y, Li B, Liu Y, Zhang L, Zuo Q, Shi L, et al. Highly sensitive oxygen sensors based on Cu(i) complex–polystyrene composite nanofibrous membranes prepared by electrospinning. Chem Commun 2009:5868. 10.1039/b910305h. 10.1039/b910305h 19787124

[73] Wang L-Y, Xu Y, Lin Z, Zhao N, Xu Y (2011). Electrospinning fabrication and oxygen sensing properties of Cu(I) complex–polystyrene composite microfibrous membranes. JLumin.

[74] Wang Y, Li B, Zhang L, Zuo Q, Li P, Zhang J (2011). High-performance oxygen sensors based on EuIII complex/polystyrene composite nanofibrous membranes prepared by electrospinning. ChemPhysChem.

[75] Yingkui L (2011). High performance oxygen sensing nanofibrous membranes of Eu(III) complex/polystyrene prepared by electrospinning. Spectrochim Acta Part A Mol Biomol Spectrosc.

[76] Wolf C, Tscherner M, Köstler S, Ribitsch V (2014). Optochemical sensors based on polymer nanofibers with ultra-fast response characteristics. IEEE SENSORS.

[77] Wolf C, Tscherner M, Köstler S (2015). Ultra-fast opto-chemical sensors by using electrospun nanofibers as sensing layers. Sens Actuators B Chem.

[78] Kai R, Jun W, Huali J (2017). Electrospinning fibrous films doped with iridium complexes for high performance oxygen sensing: synthesis and characterization. Sens Actuators B Chem.

[79] Lee S-K, Okura I (1998). Photoluminescent determination of oxygen using metalloporphyrin-polymer sensing systems. Spectrochim Acta Part A Mol Biomol Spectrosc.

[80] Borisov SM, Zenkl G, Klimant I (2010). Phosphorescent platinum(II) and palladium(II) complexes with azatetrabenzoporphyrins—new red laser diode-compatible indicators for optical oxygen sensing. ACS Appl Mater Interfaces.

[81] Payne SJ, Fiore GL, Fraser CL, Demas JN (2010). Luminescence oxygen sensor based on a ruthenium(II) star polymer complex. Anal Chem.

[82] Tian Y, Shumway BR, Gao W, Youngbull C, Holl MR, Johnson RH, et al. Influence of matrices on oxygen sensing of three sensing films with chemically conjugated platinum porphyrin probes and preliminary application for monitoring of oxygen consumption of Escherichia coli (E. coli). Sens Actuators B Chem 2010;150:579–87. 10.1016/j.snb.2010.08.036. 10.1016/j.snb.2010.08.036 PMC2976577 21076638

[83] Koren K, Hutter L, Enko B, Pein A, Borisov SM, Klimant I (2013). Tuning the dynamic range and sensitivity of optical oxygen-sensors by employing differently substituted polystyrene-derivatives. Sens Actuators B Chem.

[84] Lee S, Park J-W (2017). Luminescent oxygen sensors with highly improved sensitivity based on a porous sensing film with increased oxygen accessibility and photoluminescence. Sens Actuators B Chem.

[85] Yang X, Li L, Yuan L, Li S, Luo S, Liu Y (2011). Submicrometer organic silica gel fiber for oxygen sensing. Opt Lett.

[86] Xue R, Behera P, Viapiano MS, Lannutti JJ (2013). Rapid response oxygen-sensing nanofibers. Mat Sci and Eng C.

[87] Xue R, Behera P, Xu J, Viapiano MS, Lannutti JJ (2014). Polydimethylsiloxane core–polycaprolactone shell nanofibers as biocompatible, real-time oxygen sensors. Sens Actuators B Chem.

[88] Presley K, Hwang J, Cheong S, Tilley R, Collins J, Viapiano M (2017). Nanoscale upconversion for oxygen sensing. Mat Sci and Eng C.

[89] Akram M, Mei Z, Shi J, Wen J, Khalid H, Jiang J (2018). Electrospun nanofibers and spin coated films prepared from side-chain copolymers with chemically bounded platinum (II) porphyrin moieties for oxygen sensing and pressure sensitive paints. Talanta.

[90] Rowsell JLC, Yaghi OM (2004). Metal–organic frameworks: a new class of porous materials. Microporous Mesoporous Mater.

[91] Lu W, Wei Z, Gu Z-Y, Liu T-F, Park J, Park J (2014). Tuning the structure and function of metal–organic frameworks via linker design. Chem Soc Rev.

[92] Zhang Y, Yuan S, Day G, Wang X, Yang X, Zhou H-C (2018). Luminescent sensors based on metal-organic frameworks. Coord Chem Rev.

[93] Ni J, Wei K-J, Min Y, Chen Y, Zhan S, Li D (2012). Copper(i) coordination polymers of 2,2′-dipyridylamine derivatives: syntheses, structures, and luminescence. Dalton Trans.

[94] Tang Y-Y, Ding C-X, Ng S-W, Xie Y-S (2013). Syntheses, structures and photoluminescence of Zn(ii), Ag(i), Cu(i) and Co(ii) coordination polymers of a tetrapyridyl ligand. RSC Adv.

[95] Cui Y, Chen B, Qian G (2014). Lanthanide metal-organic frameworks for luminescent sensing and light-emitting applications. Coord Chem Rev.

[96] Dou Z, Yu J, Cui Y, Yang Y, Wang Z, Yang D (2014). Luminescent metal–organic framework films as highly sensitive and fast-response oxygen sensors. J Am Chem Soc.

[97] Burger T, Winkler C, Dalfen I, Slugovc C, Borisov SM. Porphyrin based metal–organic frameworks: highly sensitive materials for optical sensing of oxygen in gas phase. J Mater Chem C 2021:10.1039.D1TC03735H. 10.1039/D1TC03735H.

[98] Xie Z, Ma L, deKrafft KE, Jin A, Lin W (2010). Porous phosphorescent coordination polymers for oxygen sensing. J Am Chem Soc.

[99] Barrett SM, Wang C, Lin W (2012). Oxygen sensing via phosphorescence quenching of doped metal–organic frameworks. J Mater Chem.

[100] Ho M-L, Chen Y-A, Chen T-C, Chang P-J, Yu Y-P, Cheng K-Y (2012). Synthesis, structure and oxygen-sensing properties of Iridium(iii)-containing coordination polymers with different cations. Dalton Trans.

[101] Qi X-L, Liu S-Y, Lin R-B, Liao P-Q, Ye J-W, Lai Z (2013). Phosphorescence doping in a flexible ultramicroporous framework for high and tunable oxygen sensing efficiency. ChemComm.

[102] Chen Y-T, Lin C-Y, Lee G-H, Ho M-L (2015). Four new lead(II)–iridium(II) heterobimetallic coordination frameworks: synthesis, structures, luminescence and oxygen-sensing properties. CrystEngComm.

[103] Ye J-W, Lin J-M, Mo Z-W, He C-T, Zhou H-L, Zhang J-P (2017). Mixed-lanthanide porous coordination polymers showing range-tunable ratiometric luminescence for O2 sensing. Inorg Chem.

[104] Lin R-B, Li F, Liu S-Y, Qi X-L, Zhang J-P, Chen X-M (2013). A noble-metal-free porous coordination framework with exceptional sensing efficiency for oxygen. Angew Chem.

[105] Ye J-W, Zhou H-L, Liu S-Y, Cheng X-N, Lin R-B, Qi X-L (2015). Encapsulating pyrene in a metal–organic zeolite for optical sensing of molecular oxygen. Chem Mater.

[106] Lin R-B, Zhou H-L, He C-T, Zhang J-P, Chen X-M (2015). Tuning oxygen-sensing behaviour of a porous coordination framework by a guest fluorophore. Inorg Chem Front.

[107] Zhao Z, Ru J, Zhou P, Wang Y, Shan C, Yang X (2019). A smart nanoprobe based on a gadolinium complex encapsulated by ZIF-8 with enhanced room temperature phosphorescence for synchronous oxygen sensing and photodynamic therapy. Dalton Trans.

[108] Knedel T-O, Buss S, Maisuls I, Daniliuc CG, Schlüsener C, Brandt P (2020). Encapsulation of phosphorescent Pt(II) complexes in Zn-based metal–organic frameworks toward oxygen-sensing porous materials. Inorg Chem.

[109] Xie J, Chen X, Li H, Chen Z (2021). On bio-MOF materials doped with phosphorescent iridium complexes for molecular oxygen determination: synthesis, characterization and performance. Spectrochim Acta A Mol Biomol Spectrosc.

[110] Dong X-Y, Si Y, Yang J-S, Zhang C, Han Z, Luo P (2020). Ligand engineering to achieve enhanced ratiometric oxygen sensing in a silver cluster-based metal-organic framework. Nat Commun.

[111] Xia T, Jiang L, Zhang J, Wan Y, Yang Y, Gan J (2020). A fluorometric metal-organic framework oxygen sensor: from sensitive powder to portable optical fiber device. Microporous Mesoporous Mater.

[112] Xu X-Y, Yan B (2016). Nanoscale LnMOF-functionalized nonwoven fibers protected by a polydimethylsiloxane coating layer as a highly sensitive ratiometric oxygen sensor. J Mater Chem C.

[113] Liu S-Y, Qi X-L, Lin R-B, Cheng X-N, Liao P-Q, Zhang J-P (2014). Porous Cu(I) triazolate framework and derived hybrid membrane with exceptionally high sensing efficiency for gaseous oxygen. Adv Funct Mater.

[114] Feng D, Chung W-C, Wei Z, Gu Z-Y, Jiang H-L, Chen Y-P (2013). Construction of ultrastable porphyrin Zr metal–organic frameworks through linker elimination. J Am Chem Soc.

[115] Yang J, Wang Z, Li Y, Zhuang Q, Gu J (2016). Real-time monitoring of dissolved oxygen with inherent oxygen-sensitive centers in metal–organic frameworks. Chem Mater.

[116] Lan G, Ni K, You E, Wang M, Culbert A, Jiang X (2019). Multifunctional nanoscale metal–organic layers for ratiometric pH and oxygen sensing. J Am Chem Soc.

[117] Vander Donckt E, Camerman B, Hendrick F, Heme R, Vandeloise R (1994). Polystyrene immobilized Ir(III) complex as a new material for optical oxygen sensing. Bull Soc Chim Belges.

[118] Mao Y, Mei Z, Wen J, Li G, Tian Y, Zhou B (2018). Honeycomb structured porous films from a platinum porphyrin-grafted poly(styrene-co-4-vinylpyridine) copolymer as an optical oxygen sensor. Sens Actuators B Chem.

[119] Mao Y, Zhao Q, Pan T, Shi J, Jiang S, Chen M (2017). Platinum porphyrin/3-(trimethoxysily)propylmethacrylate functionalized flexible PDMS micropillar arrays as optical oxygen sensors. New J Chem.

[120] Banerjee S, Arzhakova OV, Dolgova AA, Papkovsky DB (2016). Phosphorescent oxygen sensors produced from polyolefin fibres by solvent-crazing method. Sens Actuators B Chem.

[121] McKeown NB, Budd PM (2006). Polymers of intrinsic microporosity (PIMs): organic materials for membrane separations, heterogeneous catalysis and hydrogen storage. Chem Soc Rev.

[122] Low Z-X, Budd PM, McKeown NB, Patterson DA (2018). Gas permeation properties, physical aging, and its mitigation in high free volume glassy polymers. Chem Rev.

[123] Kameda M, Tezuka N, Hangai T, Asai K, Nakakita K, Amao Y (2004). Adsorptive pressure-sensitive coatings on porous anodized aluminium. Meas Sci Technol.

[124] Araki N, Amao Y, Funabiki T, Kamitakahara M, Ohtsuki C, Mitsuo K (2007). Optical oxygen-sensing properties of porphyrin derivatives anchored on ordered porous aluminium oxide plates. Photochem Photobiol Sci.

[125] Baron AE, Danielson JDS, Gouterman M, Wan JR, Callis JB, McLachlan B (1993). Submillisecond response times of oxygen-quenched luminescent coatings. Rev Sci Instrum.

[126] Amao Y, Miyakawa K, Okura I (2000). Novel optical oxygen sensing device: a thin film of a palladium porphyrin with a long alkyl chain on an alumina plate. J Mater Chem.

[127] Amao Y, Okura I (2000). An oxygen sensing system based on the phosphorescence quenching of metalloporphyrin thin film on alumina plates. Analyst.

[128] Amao Y, Ishikawa Y, Okura I, Miyashita T (2001). Optical oxygen sensing material: terbium(III) complex adsorbed thin film. Bull Chem Soc Jpn.

[129] Fernández-Sánchez JF, Cannas R, Spichiger S, Steiger R, Spichiger-Keller UE (2006). Novel nanostructured materials to develop oxygen-sensitive films for optical sensors. Anal Chim Acta.

[130] Marin-Suarezdel Toro M, Fernandez-Sanchez JF, Baranoff E, Nazeeruddin MdK, Graetzel M, Fernandez-Gutierrez A. Novel luminescent Ir(III) dyes for developing highly sensitive oxygen sensing films. Talanta 2010;82:620–6. 10.1016/j.talanta.2010.05.018. 10.1016/j.talanta.2010.05.018 20602945

[131] McDonagh C, Kolle C, McEvoy AK, Dowling DL, Cafolla AA, Cullen SJ (2001). Phase fluorometric dissolved oxygen sensor. Sens Actuators B Chem.

[132] Lehner P, Staudinger C, Borisov SM, Regensburger J, Klimant I (2015). Intrinsic artefacts in optical oxygen sensors-how reliable are our measurements?. Chem Eur J.

[133] Langsam M, Robeson LM. Substituted propyne polymers? part II. Effects of aging on the gas permeability properties of poly[1-(trimethylsilyl)propyne] for gas separation membranes. Polym Eng Sci 1989;29:44–54. 10.1002/pen.760290109.

[134] Müller BJ, Burger T, Borisov SM, Klimant I (2015). High performance optical trace oxygen sensors based on NIR-emitting benzoporphyrins covalently coupled to silicone matrixes. Sens Actuators B Chem.

[135] Larsen M, Lehner P, Borisov SM, Klimant I, Fischer JP, Stewart FJ (2016). In situ quantification of ultra-low O2 concentrations in oxygen minimum zones: application of novel optodes: In situ trace sensing of O2 using novel optodes. Limnol Oceanogr Methods.

[136] Borisov SM, Klimant I (2007). Ultrabright oxygen optodes based on cyclometalated iridium(III) coumarin complexes. Anal Chem.

[137] Velasco-García N, Pereiro-García R, Diaz-García ME (1995). Analytical and mechanistic aspects of the room temperature phosphorescence of Erythrosine B adsorbed on solid supports as oxygen sensing phases. Spectrochim Acta Part A Mol Biomol Spectrosc.

