# Supplementary material for: Porous matrix materials in optical sensing of gaseous oxygen
Source: Anal Bioanal Chem. 2022 Mar 29;414(15):4311–30. doi: 10.1007/s00216-022-04014-6 (PMC9142480; doi:10.1007/s00216-022-04014-6)
Supplement: Supplementary file 1 — Supplementary file1 The ESI contains an overview of abbreviations, information on commercial availability of the discussed sensor matrices and detailed data on matrix materials used in optical oxygen gas sensors and their properties (porosity, response time, KSV, kq and linearity of SV plot). (PDF 639 KB) [file 216_2022_4014_MOESM1_ESM.pdf]

Analytical and Bioanalytical Chemistry

Electronic Supplementary Material

Porous matrix materials in optical sensing of gaseous oxygen

I. Dalfen, S.M. Borisov*

Institute of Analytical Chemistry and Food Chemistry, Graz University of Technology, Stremayrgasse 9, 8010 Graz, Austria

*Corresponding Author: Assoc.Prof. Sergey M. Borisov, sergey.borisov@tugraz.at, ORCID: 0000-0001-9318-8273

1. Abbreviations

1.1. Dyes/Ligands

TMS-DAP	*N*-(3-trimethoxysilylpropyl)-2,7-diazapyrenium bromide
PBA	pyrene butylic acid
C480	Coumarin 480
PtCN(L)	Cyanido-(κ3CNN-*N*-(6-phenylpyridin-2-yl)-*N*-propyl-thiazol-2-amino)-platinum(II)
AlQ_3	tris(8-hydroxyquinolinato)aluminium

Polypyridyl ligands

dpp	4,7-diphenyl-1,10-phenanthroline
dC_8pp	4,7-(bis-4-octylphenyl)-1,10-phenanthroline
phen	1,10-phenanthroline
bpy/phen -Si	Bpy or phen ligand linked to trialkoxysilanes via an alkyl chain linker
$diC_{19}phen$	4,7-dinonadecyl-1,10-phenanthroline
ppy	2-phenylpyridine
4-cppy	2-(4-carboxyphenyl)pyridine
F_2ppy	2-(2,4-difluorophenyl)pyridine
bpy	2,2'-bipyridine
Brbpy	4-bromo-2,2'bipyridine
$Bpy-NH_2$	3-amino-2,2'-bipyridine
4-dcbpy	4,4'-dicarboxy-2,2'-bipyridine
5- dcbpy	5,5'-dicarboxy-2,2'-bipyridine
Me_4dabpy	$N^4,N^4,N^{4'},N^{4'}$-tetramethyl-(4,4'-diamine-2,2'-bipyridine)
DMCH	6,7-dihydro-5,8-dimethyl-dibenzo-[*i,j*][1,10]-phenanthroline
OMCH	5,8-dimethyl-dibenzo-[*i,j*][1,10]-phenanthroline
iphen	1H-imidazo[4,5-f]-[1,10]phenanthroline
pip	2-phenyl-1H-imidazo[4,5-f]-[1,10]phenanthroline
epip	1-ethyl-2-phenyl-1H-imidazo[4,5-f][1,10]phenanthroline
nip	2-(naphthalen-1-yl)-1H-imidazo[4,5-f][1,10]phenanthroline
Phencarz	2-(*N*-ethyl-carbazole-yl-4)imidazo[4,5-f]1,10-phenanthroline
eCl-phen	1-ethyl-2-(*N*-ethyl-carbazole-yl-4)imidazo[4,5-f]1,10-phenanthroline

Porphyrins

H_2OEP	2,3,7,8,12,13,17,18-octaethyl--21*H*,23*H*-porphyrin
H_2BP	tetrabenzo-21*H*,23*H*-porphyrine
H_2TPP	5,10,15,20-tetraphenyl-21*H*,23*H*-porphyrin
H_2TCPP	5,10,15,20-tetrakis-(4-carboxyphenyl) -21*H*,23*H*-porphyrin
H_2CTPP	5-(4-carboxyphenyl)-10,15,20-triphenyl-21*H*,23*H*-porphyrin
$H_2TCBPyP$	5,10,15,20-tetra{4-[(Ncarbazyl)butyloxyphenyl]}porphyrin
$H_2TMCUPP$	5,10,15,20-tetrakis-[4-(11-methoxycarbonylundecane-1-oxy)phenyl]- 21*H*,23*H*-porphyrin
$H_2MCUTPP$	5-[4-(11-methoxycarbonylundecane-1-oxy)phenyl]-10,15,20-triphenyl-21*H*,23*H*-porphyrin
H_2TFPP	tetrakis(pentafluorophenyl) -21*H*,23*H*-porphyrin
H_2TPPMA	10,15,20-triphenyl-5-(2-(methacryloyloxy)ethyl benzoate) -21*H*,23*H*-porphyrin
H_2PyP	tetrapyridinie-21*H*,23*H*-porphyrin
H_2MPyP	tetra(*N*-methyl-pyridinie)-21*H*,23*H*-porphyrin
$H_2PC_{10}COOH$	[5-(1-carboxydecyl-4-pyridyl(10,15,20-tritoloyl-21*H*,23TMMB 1,3,5-tris[2-(2′-pyridyl)benzimidazoyl] methylbenzene
dpephos	bis[2-(diphenylphosphanyl)phenyl] ether
POP	bis[2-(diphenylphosphino)phenyl]ether
TTA	2-theonyltrifluoroacetate
Pyr	1-pyrenol
1,4-H_2NDC	1,4-naphthalenedicarboxylic acid
HS^tBu	*tert*-buthylthiol
Ferron	8-hydroxy-7-iodo-5-quinolinesulfonic acid
Tfpb	tetrakis (bis-3, 5-trifluoromethylphenylborate)

$BisPh_2PPh$	bis(2-(diphenylphosphanyl)phenyl)ether
PPh_3	triphenylphosphane

1.2. polymers

PMMA	poly(methyl methacrylate)
pPFPA	poly(pentafluorophenyl acrylate)
pOCFPM	poly(octafluoropentyl methacrylate)
PS	polystyrene
pPFS	poly(pentafluorostyrene)
EC	ethylcellulose
O-PP	oxidized polypropylene
PEG	polyethyleneglycole
PVA	polyvinyl alcohol
pTMSP	poly[1-(trimethylsilyl)-1-propyne]

1.3. Sol-gel precursors

TMOS	tetramethoxysilane
TEOS	tetraethoxysilane
MTMOS	methyltrimethoxysilane
MTEOS	methyltriethoxysilane
alkyl-triMOS	alkyltrimethoxysilane
alkyl-triEOS	alkyltriethoxysilane
TFP-triMOS	3,3,3-trifluoropropyltrimethoxysilane
PFPh-triMOS	3-pentafluorophenylpropyl trimethoxysilane
PhE-triMOS	2-phenylethyltrimethoxysilane

1.4. Miscellaneous

NW	nanowire
CD	carbon dot
NP	nanoparticle
UCNP	up-conversion nanoparticle
Apd	average pore diameter

2. Overview of commercial availability of described matrix materials

Table S1. *Overview of porous and nonporous oxygen sensing materials is respect to their commercial availability and manufacturing effort*

**Material type**	**Commerical availability**	**Common name(s)**	**Preparation effort**
Silica gel beads	Yes	Partisil™, Silica gel	n.a.
Mesoporous silica	Yes	MCM-41, SBA-15	Medium<br>(Template synthesis from TEOS and surfactant, followed by washing and calcination)
Silica aerogels	No		High<br>(sol-gel process with drying in supercritical CO_2)
Sol-gels (silica- and Ormosil-based)	No		Medium<br>(sol-gel process; tuning of conditions has influence on properties)
MOFs	Some	Respective MOF name (ZIF-8, HKUST-1, PCN-224,…)	Medium to high<br>(Usually solvothermal synthesis, conditions can affect morphology)
Electrospun nanofibers	No		Medium<br>(extrude polymer solution under high voltage)
Porous anodized alumina	Yes	Porous anodized alumina, AAO	Medium<br>(electrochemical oxidation under acidic conditions)
Non-porous polymers (like polystyrene)	Yes	Poly(styrene)	Low (coating of solution of the dye and polymer solution in organic solvent)
Perfluorinated amorphous polymers (Teflon AF, Hyflon AD)	Yes	Polymer name or trademark names (Hyflon AD,…)	Low to high (simple coating as in case of non-porous polymers, but synthesis of compatible dyes may be challenging)

Silicone rubber	Yes	E4, RTV-silicone, Silicone rubber	Low to high (polymerization/crosslinking from commercially available precursors). Synthesis of dyes may be challenging.

3. Summary on the porosity and sensing properties for sensors based on different classes of porous matrix materials[1]

Table S2. *Overview of oxygen sensing materials based on silica aerogel matrices*

Indicator	Matrix	Porosity	K_{SV} / kPa^{-1}	k_q / $Pa^{-1}s^{-1}$	Response / Recovery time / s	Linearity of SV plot	Ref
TMS-DAP$^+$	TMOS	N_2 BET: 870 m^2g^{-1}	0.005	330	<8	?	[66]
$Ru(phen)_3$ donor-acceptor dyads	TMOS	N_2 BET: 1000 m^2g^{-1}	2.5	1020	<8	No (0-<10%)	[67]
$Ru(dpp)_3$ a)	TMOS aerogel		14	2800	<10	No (0-<5%)	[68]
$Ru(dpp)_3$ a)	TMOS xerogel		10	2000	<50		
PtOEP a)	TMOS aerogel		0.5	5	<10	No (0-5.5%)	

a) τ_0 not given for the indicator in this material but estimated based on other literature

b) first calibration point at 20% O_2

Table S3. *Overview of oxygen sensing materials based on silica-gel with surface-adsorbed indicators*

Indicator	Matrix	Porosity	K_{SV} / kPa^{-1}	k_q / $Pa^{-1}s^{-1}$	Response / Recovery time / s	Linearity of SV plot	Ref
Perylene tetracarboxylic acid bisimidesa)	Silica (TLC plate)		0.08	16000	-	?	[26]
$Ru(bpy)_2(DMCH)$	Silica (Partisil 5) in E4 silicone	350 m^2g^{-1}, 85 Å apd	0.021	60	35	No (0-<20%)	[28]
$Ru(bpy)(DMCH)_2$			0.021	40			
$Ru(bpy)_2(OMCH)$			0.045	120			
$Ru(dpp)(DMCH)_2$	PVC		0.018	30	1		
	E4 silicone		0.02	30	1		

[1] For simplified navigation the reference numbers are identical to those in the Review.

Ru(bpy)$_3$ [a)]	Silica gel in silanol-silanol condensed PDMS<br>Silica gel in vinyl-hydride condensed PDMS		0.02<br>0.03	20<br>30		No	[27]
Ru(bpy)$_3$ [a)]<br>Ru(phen)$_3$ [a)]<br>Ru(dpp)$_3$ [a)]	Cab-O-Sil fumed silica discs	196 m^{2}g^{-1}	0.29<br>0.83<br>1.4	290<br>330<br>280		No (0-<5%)	[16]
Ru(odap)$_3$ [c)]	silicone<br>Partisil in silicone<br>C-18 silica in silicone<br>CPG in silicone<br>Amberlite XAD-4 in silicone	 350 m^{2}g^{-1}, 85 Å apd<br>500 m^{2}g^{-1}, 60 Å apd<br>200-400 mesh, 75 Å apd<br>750 m^{2}g^{-1}, 100 Å	0.01<br>0.10<br>0.02<br>0.14<br>0.03	10<br>210<br>40<br>290<br>60		Yes<br>No	[29]
Ru(dpp)$_3$ [a)]	Silicone<br>Fumed silica LM-130 in silicone<br>Fumed silica HS-5 in silicone<br>Fumed silica EH-5 in silicone	 130 m^{2}g^{-1}<br>325 m^{2}g^{-1}<br>380 m^{2}g^{-1}	0.05<br>0.13<br>0.13<br>0.13	10<br>30<br>30<br>30	25 / 48<br>27 / 64<br>30 / 74<br>24 / 72	No (K_q calculated from I_0/I_{100})	[138]
Ru(bpy)$_3$ [a)]	Mesoporous silica	550 m^{2}g^{-1}	0.01	11	120-240	No	[139]
PdOEP<br>PtOEP<br>PtTFPP	SBA-15	960 m^{2}g^{-1}<br>89 Å apd	48<br>4.7<br>1.5	50<br>60<br>20		No	[13]

PtTPP<br>Ru(dpp)$_3$<br>Ru(phen)$_2$(phen-CH$_3$)<br>Ru(phen)$_3$<br>Ru(bpy)$_3$			2.2<br>0.4<br>0.2<br>0.3<br>0.1	30<br>50<br>50<br>90<br>90			
PtMPyP^{4+} [a)]<br>PtPyP [a)]	MCM-41		5<br>0.33	100<br>7	0.75 / 204<br>0.33 / 26.62	No	[38]
Ru$_3$(bpy)$_6$TMMB<br><br>Ru$_3$(phen)$_6$TMMB	SBA-15<br>MCM-41<br>SBA-15<br>MCM-41		0.10<br>0.06<br>0.21<br>0.20	100<br>60<br>80<br>80	3 / 30	No<br>(0-<10%)	[39]
[Cu(dpephos)(pip)]BF$_4$<br><br>[Cu(dpephos)(epip)]BF$_4$<br><br>[Cu(dpephos)(nip)]BF$_4$<br><br>[Cu(dpephos)(enip)]BF$_4$	MCM-41<br>SBA-15<br>MCM-41<br>SBA-15<br>MCM-41<br>SBA-15<br>MCM-41<br>SBA-15		0.4<br>2.0<br>0.8<br>2.0<br>0.5<br>3.5<br>0.8<br>1.6	50<br>250<br>400<br>1030<br>65<br>500<br>340<br>680	8 / 180	No<br>(0-<1%)	[40]
[{Re(CO)$_3$(diC$_{19}$phen)}$_2$(bpy)](OTf)$_2$	MCM-41<br>SBA-15		0.3<br>1.3	70<br>160	19 / 41<br>7 / 43	No<br>(0-<10%)	[41]
Ru(dpp)$_3$ [a)]	Silica in Octyl-triEOS/TEOS<br><br>Octyl-triEOS/TEOS	250 m^{2}g^{-1}, 19.5 Å apd	0.25<br><br>0.03	50<br><br>5		Yes	[140]
PtTFPP [a)]	Ag-coated silica in Octyl-triEOS/TEOS<br><br>Octyl-triEOS/TEOS	250 m^{2}g^{-1}, 19.5 Å apd	1.6<br><br>0.2	20<br><br>2	2.6 / 36<br><br>1.1 / 5.5	No (~0-20%, k_q calculated from I_0/I_{100})<br><br>Yes	[141]

[Cu(PTO)(PPh_3)$_2$]BF_4	MCM-41		0.9	13	9 / 37	No (0-<10%)	[42]
PtTCBPP [a)]	MCM-41<br>SBA-15		354<br>130	3500<br>1300	3 / 17<br>1.5 / 14.5	No (0-<10%)	[43]
PtTFPP	Silica gel	480 m^2g^{-1}, 60 Å apd	0.3	7	4 / 7	Yes (0-20%)	[33]

a) τ_0 not given for the indicator in this material but estimated based on other literature

b) first calibration point at 20% O_2

c) τ_0 only given for methanolic dye solution

Table S4. *Overview of oxygen sensing materials based on indicator covalently coupled to silica-gel*

Indicator	Matrix	Porosity	K_{SV} / kPa^{-1}	k_q / $Pa^{-1}s^{-1}$	Response / Recovery time / s	Linearity of SV plot	Ref
Erythrosine B isocyanate (covalently grafted)	Amino-functionalized silica (Bondesil)	500 m^2g^{-1}, 60 Å apd	66	230	<2s	No	[31]
Erythrosine B (adsorbed)	Amberlite XAD-2<br>Amberlite XAD-4	300 m^2g^{-1}, 90 Å<br>750 m^2g^{-1}, 100 Å	1.6<br>1.7	2<br>2	<2s	No	[137]
$Ru(bpy)_2$(phen-Si-) (covalently grafted)	MCM-41<br>Amorphous silica/		0.13<br>0.08	74<br>53	4 / 10	Yes	[35]
$Ru(bpy)_2$(phen) (adsorbed)	MCM-41<br>Amorphous silica		0.47<br>0.35	340<br>210	4 / 10	No (0-<10%)	[35]
$Ru(dpp)_2$(phen-Si-) (covalently grafted)<br><br>$Ru(dpp)_2$(phen) (adsorbed)	MCM-41<br>SBA-15<br>MCM-41<br>SBA-15	706 m^2g^{-1}, 71.3 Å apd<br>615 m^2g^{-1} 69.6 Å apd<br>1209 m^2g^{-1} 32.7 Å apd<br>765 m^2g^{-1} 31.2 Å apd	2.0<br>0.62<br>0.74<br>0.28	280<br>190<br>120<br>90	7.5 / 22.5<br>11.5 / 47.5<br>3.2 / 20.6<br>4 / 27.5	No	[36]
$Ru(dpp)_2$(bpy-Si)	MCM-41		3.1	920	1.2 / 29	No (0-1.4%)	[37]
PtTFPP<br><br>PdTFPP	Silica gel in E4 silicone (adsorbed)<br>Amine-modified silica gel in E4 silicone (covalent)<br>Silica gel in E4 silicone (adsorbed)	500 m^2g^{-1}, 60 Å apd (unmodified silica gel)	3.9<br>3.5<br>63	55<br>50<br>65	0.15	Yes	[32]

	Amine-modified silica gel in E4 silicone (covalent)		62	65			
Ir-OEP-(*n*-ButIm)$_2$<br>Ir-OEP-(CarbIm)$_2$<br>(covalently grafted)	PS<br>Amino modified silica	550 m^{2}g^{-1}, 60 Å apd (unmodified silica gel)	0.14<br>1.8	4<br>70		Yes<br>No (0-0.4%)	[30]
Ru(bpy)$_2$(bpy-Si) (covalently grafted)	MSU-3	84 Å apd in undoped MSU-3	0.55	190	3 / 21	No (0-<10%)	[142]
Ru(bpy)$_2$(bpy-Si) (adsorbed)			0.32	190	7.5 / 33.5		

a) τ_0 not given for the indicator in this material but estimated based on other literature

b) first calibration point at 20% O_2

Table S5. *Overview of oxygen sensing materials based on silica and ORMOSIL sol-gel matrices*

**Indicator**	**Matrix**	**Porosity**	**K_{SV} / kPa^{-1}**	**k_q / $Pa^{-1}s^{-1}$**	**Response / Recovery time / s**	**Linearity of SV plot**	**Ref**
$Ru(bpy)_3$ a)	TEOS sol-gel film		0.01	10	<5	No (0-<10%)	[45]
$Ru(dpp)_3$ a)	TEOS sol-gel film		0.07	15	<5		[46]
$Ru(dpp)_3$	TEOS sol-gel film		0.06	10	<5		[47]
PtOEP a)	TEOS sol-gel film		0.07	1	5 / 10	No	[49]
	TEOS sol-gel monolith		0.38	4	1-9 min	Yes	[48]
Al-ferron	TMOS/MTMOS 1:1		0.17	0.4	5s	No (0-<10%)	[143]
$Ru(dpp)_3$ a)	TEOS sol-gel		0.07	10	<1		[50]
	MTEOS/TEOS 1:1		0.13	30			
	MTEOS/TEOS 2:1		0.11	20			
	MTEOS/TEOS 3:1		0.08	20			
	MTEOS		0.06	10			
	ETEOS/TEOS 1:1		0.11	20			
	ETEOS		0.07	10			
$Ru(dpp)_3$ in bulk xerogel (0.02-0.1 mm, prepared by casting)	TEOS		0.02	5		Yes	[51]
	TEOS/MTMS 9/1		0.04	8		No	
	TEOS/MTMS 8/2		0.04	8			
	TEOS/MTMS 7/3		0.07	14			
	TEOS/MTMS 6/4		0.09	17			
	TEOS/MTMS 5/5		0.11	19			
	TEOS/MTMS 4/6		0.13	21			
	TEOS/MTMS 3/7		0.20	32			
	TEOS/MTMS 2/8		0.19	29			
	TEOS/MTMS 1/9		0.22	32			
	MTMS		0.27	38			
$Ru(dpp)_3$ in spin-coated thin films (<200 nm thick)	TEOS	19.1%	0.20	46			
	TEOS/MTMS 9/1		0.23	50			
	TEOS/MTMS 8/2		0.24	48			
	TEOS/MTMS 7/3		0.11	22			
	TEOS/MTMS 6/4		0.09	17			
	TEOS/MTMS 5/5	9.2%	0.11	19			

	TEOS/MTMS 4/6		0.17	27			
	TEOS/MTMS 3/7		0.13	21			
	TEOS/MTMS 2/8		0.08	12			
	TEOS/MTMS 1/9		0.05	7			
	MTMS	3.4%	0.03	5			
PtOEP	PS		0.18	2	3	No (0-10%)	[144]
$Ru(dpp)_3$			0.01	2	3		
PtOEP	Phenyl-triMOS/ trimethylmethoxysilane (18:1)		0.45	5	0.25		
$Ru(dpp)_3$			0.02	5	0.25		
$Ru(bpy)_2$(bpy-Si) [a)]	MTEOS		0.005	5	<30	Yes[b)]	[145]
Erythrosine B	Aminopropyl-triEOS/MTMOS/TMOS (32/40/28)		116	420	5-7	No (0-<5%)	[146]
	MTMOS/TMOS (60/40)		70	250			
Erythrosine B	TMOS sol gel (6·6·12 mm)		3	11	<1	No (0-<10%)	[147]
Erythrosine B	TMOS sol gel (6·6·12 mm)		650	2300	<1	No (0-<5%)	[148]
$Ru(dpp)_3$	TEOS PEBBLEs (100-600nm)		0.11	20		Yes	[34]
$Ru(dpp)_3$	TEOS (H_2O:precursor ratio R=2)	4.2%	0.01	3	0.106		[12]
		5.5%	0.04	8	0.078		
		9.5%	0.06	13	0.04		
	TEOS R=4	4.5%	0.04	7	0.539		
		5.7%	0.05	10	0.462		
	MTEOS (R=2)	6.4%	0.03	7	0.0049		
	MTEOS (R=4)	4.0%	0.03	5	0.0049		
$Ru(dpp)_3$	TEOS sol-gel		1.0	370	<5	No (0-<5%)	[53]
	Octyl-triEOS/TEOS (2/8)		0.08	10		Yes	
	Octyl-triEOS/TEOS (4/6)		0.11	20			
	Octyl-triEOS/TEOS (5/5)		0.12	20			
	Octyl-triEOS/TEOS (6/4)		0.15	30			
$Ru(dpp)_3$	Propyl-triMOS/TFP-triMOS (1:2)		0.34	50		Yes[b)]	[61]
$Ru(dpp)_3$	TFP-triMOS/TMOS (10:1)		0.15	30		Yes[b)]	[62]

	TFP-triMOS/Propyl-triMOS (1:1)		0.34	60			
	PFPh-triMOS/PhE-triMOS (1:2)		0.04	6			
PtTFPP [a)]	Octyl-triEOS/TEOS (3.5:96.5)		0.21	3	0.6 / 5	Yes	
PtOEP [a)]			0.46	5	0.7 / 14		
PtTFPP [a)]	TFP-triMOS/Propyl-triTMOS (2:1)		0.66	8	3.7 / 5.3	Yes[b)]	[63]
PtOEP [a)]			0.84	8	3.7 / 5.9		
$Ru(dpp)_3$	Methyl-triMOS/TMOS (1:1)	$D \sim 2.5 \cdot 10^{-7}$ cm^2s^{-1}	0.02	4		Yes	[52]
	Ethyl-triMOS/TMOS (1:1)	$D \sim 5 \cdot 10^{-7}$ cm^2s^{-1}	0.03	7			
	Propyl-triMOS/TMOS (1:1)	$D \sim 9 \cdot 10^{-7}$ cm^2s^{-1}	0.08	15			
	Butyl-triMOS/TMOS (1:1)	$D \sim 13 \cdot 10^{-7}$ cm^2s^{-1}	0.10	20			
	Hexyl-triMOS/TMOS (1:1)	$D \sim 16 \cdot 10^{-7}$ cm^2s^{-1}	0.11	23			
	Octyl-triMOS/TMOS (1:1)	$D \sim 20 \cdot 10^{-7}$ cm^2s^{-1}	0.11	22			
	Decyl-triEOS/TEOS (1:1)	$D \sim 16 \cdot 10^{-7}$ cm^2s^{-1}	0.09	19			
	Dodecyl-triEOS/TEOS (1:1)	$D \sim 8 \cdot 10^{-7}$ cm^2s^{-1}	0.05	10			
$Ru(dpp)_3$	MTEOS	$D = 0.99 \cdot 10^{-7}$ cm^2s^{-1}	0.06	10	0.432	No (0-<30%)	[59]
	ETEOS	$D = 6.2 \cdot 10^{-7}$ cm^2s^{-1}	0.07	15	0.232		
	Propyl-triEOS	$D = 6.7 \cdot 10^{-7}$ cm^2s^{-1}	0.11	20	0.223		

	Phenyl-triEOS	D= 0.003 $\cdot 10^{-7}$ cm^2s^{-1}	0.02	4	2.687		
PtTFPP<br>PtOEP	MTEOS		0.27<br>0.48	3<br>4	15 / 60	Yes	[149]
$Ru(dpp)_3$	Phenyl-triEOS<br>Methyl-triEOS<br>Ethyl-triEOS<br>Propyl-triEOS<br>Phenyl-triEOS/TFP-triMOS (1:1)<br>Methyl-triEOS/TFP-triMOS (1:1)<br>Ethyl-triEOS/TFP-triMOS (1:1)<br>Propyl-triTEOS/TFP-triMOS (1:1)		0.02<br>0.04<br>0.05<br>0.09<br>0.08<br>0.15<br>0.20<br>0.21	5<br>10<br>10<br>20<br>20<br>35<br>50<br>50		No (linear <5%; above very small deviation from linearity)	[60]
PtTFPP	Dye in TEOS core/shell NPs and co-immobilized with dyed NPs in Octyl-triEOS/TEOS<br>Dye and undyed TEOS NPs co-immobilized in Octyl-triEOS/TEOS<br>Dye in TEOS NP core, Dyed NPs immobilized in Octyl-triEOS/TEOS	Silica NPs: 0.69 m^2g^{-1}<br>154 Å apd	5.1<br>1.7<br>0.37	60<br>20<br>5	1.3 / 18.6<br>6.8 / 41.4	No (0-<20%)	[150]
PtTFPP	Propyl-triMOS/TEOS/octyl-triEOS (1:3.6:1.4)<br>TFP-triMOS/TEOS/octyl-triEOS (0.9:3.6:1.4)		1.4<br>0.92	20<br>10	3 / 23<br>2 / 68	Yes	[64]
$Ru(dpp)_3$	TEOS/cellulose acetate/ethylene glycol (4:1:1)		0.03	6		Yes	[151]
PdTCPP [a)]	TEOS/Octyl-triEOS (18:7)		1.5	2	2 / 43	Yes[b)]	[152]
PtTFPP	PS-co-PFS<br>Phenyl-triMOS/TEOS in silicone E4		0.10<br>6.5	2<br>120		No (0-<5%)	[19]

PdTFPP	PS-co-PFS		1.1	1			
	Phenyl-triMOS/TEOS in silicone E4		96	120			

a) τ_0 not given for the indicator in this material but estimated based on other literature

b) first calibration point at 20% O_2

Table S6. *Overview of oxygen sensing materials based on electrospun nanofibers*

Indicator	Matrix	Dimensions	K_{SV} / kPa^{-1}	k_q / $Pa^{-1}s^{-1}$	Response / Recovery time / s	Linearity of SV plot	Ref
[Cu(POP)phencarz]BF_4	PS fibers<br>PS film dip coated	Ø = 500-700 nm	0.15<br>0.07	1<br>7	7 / 14	Yes	[72]
[Cu(POP)(ECl-phen)]BF_4	PS fibers	Ø = 600 nm	0.09	1	10 / 20	Yes	[73]
[Eu(TTA)$_3$(phencarz)]	PS fibers	Ø = 400-500 nm	0.1	0.2	5 / 8	No	[74]
Eu(TTA)$_3$phen	PS fibers	Ø = 350 nm	0.02	0.1	9 / 20	Yes	[75]
PtTFPP	PS film<br>PS fibers	30 µm thick<br>620 nm broad, 100 nm thick	0.09	1	2.2 / 4<br>32 ms	No (0-<10%)	[76,77]
Ir(2-phenylbenzo [d]thiazole)$_2$(acac)<br>Ir(2-phenylbenzo [d]thiazole)$_2$(acac-F_3)<br>Ir(2-phenylbenzo [d]thiazole)$_2$(acac-F_6)	PS fibers	Ø = 1.5 µm	0.05<br>0.08<br>0.12	40<br>15<br>15	14 / 27<br>8 / 17<br>9 / 23	Yes	[78]
Ru(dpp)$_3$ $^{a)}$	TEOS/octyl-triEOS fiber	Ø = 900 nm	0.02	5	0.1	Yes$^{b)}$	[85]
Ru(dpp)$_3$ $^{a)}$	PCL fibers<br><br>PES fibers<br>PES-PCL fibers<br>PCL film	Ø = 530 nm<br>Ø = 7.01 µm<br>Ø = 720 nm<br>Ø = 960 nm	0.008<br>0.007<br>0.007<br>0.003	2<br>1<br>1<br>1<br>-	0.9 / 1.9<br>1.8 / 2.3<br>2.2 / 2.4<br>2.9 / 3.3<br>150 / 400	Yes	[86]
[Ru(dpp)$_3$]Cl_2<br>[Ru(dpp)$_3$]Ph_4B	PDMS core – PCL shell fibers	Ø = 512 nm<br>Ø = 401 nm	0.02<br>0.03	3<br>5	0.36 / 0 72<br>0.28 / 0.51	Yes	[87]

PtOEP		Ø = 570 nm	0.23	3	0.49 / 0.70		
Ru(dpp)$_3$ $^{a)}$ + LiY$_4$:Yb,Tm UCNPs	PSU core – PCL shell fibers		0.00<br>3	1	0.2 / 0.4	Yes	[88]
PtTPA<br><br>PdTPP<br>PtTPP<br>PdTPA	pTMSP fibers<br><br><br>AgNPs@pTMSP fibers	Ø = 283-727 nm<br><br>Ø = 141-467 nm	0.7<br>6<br>0.4<br>3	10<br>10<br>5<br>7	4.5	No (0-5%)<br>No (0-40%)	[153]
PtTPPMA $^{a)}$	pMMA	Nanofibers	0.01	0.1	9.15 / 15	Yes	[89]
		Spin coated films	0.01	0.2	14.40 / 41.71	No	
	p(PFPA-co-OCFPM)	Nanofibers	0.04	0.5	6.70 / 146.9	Yes	
		Spin coated films	0.01	0.1	10.05 / 61.87	No	
	p(MMA-co-PFPA-co-OCFPM)	Nanofibers	0.14	1.7	4.42 / 141.27	Yes	
		Spin coated films	0.01	0.1	10.30 / 74.47	No	
	p(S-co-PFPA-co-OCFPM)	Nanofibers	0.07	0.8	10.40 / 112.96	Yes	
		Spin coated films	0.01	0.1	16.21 / 161.95	No	
PtOEP $^{a)}$	1% AgNWs in PMMA film	AgNWs Ø=50 nm, length=100 µm ; PMMA fibers Ø~3 µm	0.09	0.1	-	No (0-10%)	[154]
	1% AgNWs in PMMA fibers		0.32	0.3	3 / 12.6		
	3% AgNWs in PMMA fibers		0.61	0.6	1.8 / 14.4	No	
Ru(bpy)$_3$	EC fibers containing Fe_2O_3	Film thickness ~ 5.12 µm, both Fe_2O_3 and Fe_2O_3@Ag NPs Ø~60 nm	0.02<br>4	13<br>0	3-20	Yes	[155]
	EC fibers containing Fe_2O_3@Ag		0.02<br>4	10<br>0			

	EC film containing Fe_2O_3<br>EC film containing Fe_2O_3@Ag		0.017<br>0.019	90<br>80			
Ru(dpp)$_3$ [a)]	MTEOS fibers containing SiCDs	BET surface = 15.22 m^2g^{-1}<br>52.4 Å “apd“ of fiber mat<br>Fiber Ø = 0.5-1 µm	0.02	5		Yes	[156]

a) τ_0 not given for the indicator in this material but estimated based on other literature

b) first calibration point at 20% O_2

Table S7. *Overview of oxygen sensitive MOFs*

MOF	Matrix / Support	Porosity	K_{SV} / kPa^{-1}	kq / $Pa^{-1}s^{-1}$	Response / Recovery time / s	Linearity of SV plot	Ref
$[Zn_4(\mu_4\text{-}O)(Ir(4\text{-}cppy)_3)_2]\cdot 6DMF\cdot H_2O$		N_2 BET: 764 m^2g^{-1}, CO_2 BET 958 m^2g^{-1}, apd 4.5 & 5.8 Å	0.01	7		Yes	[98]
$Ir(ppy)_2(5\text{-}dcppy)$@UiO-67<br>$Ir(ppy)_2(5\text{-}dcbpy)$@UiO-67<br>$Ru(bpy)_2(5\text{-}dcbpy)$@UiO-67		N_2 BET: 2568 m^2g^{-1}<br>N_2 BET: 2292 m^2g^{-1}<br>N_2 BET: 1277 m^2g^{-1}	0.78<br>0.88<br>2.25	390<br>440<br>2250		No (0-<5%)	[99]
$[Zn(Ir(ppy)_2(4\text{-}dcbpy)PF_6)_2]\cdot 3DMF\ 5H_2O$ a)<br>$[Cd(Ir(ppy)_2(4\text{-}dcbpy)PF_6)_2(H_2O)_2]\cdot 3DMF\ 6H_2O$ a)<br>$[Co(Ir(ppy)_2(4\text{-}dcbpy)PF_6)_2(H_2O)_2]\cdot 2DMF\cdot 8H_2O$ a)<br>$[Ni(Ir(ppy)_2(4\text{-}dcbpy)PF_6)_2(H_2O)_2]\cdot 3DMF\cdot 6H_2O$ a)		Channels: 8.9 Å · 8.8 Å<br>Channels: 9.3 Å · 9.3 Å<br>Channels: 9.1 Å · 9.1 Å<br>Channels: 9.1 Å · 9.1 Å	0.01<br>0.03<br>0.01<br>0.01	4<br>15<br>10<br>5	120 / 110<br>70 / 30<br>200 / 90<br>240 / 40	Yes	[100]
MAF-X11		N_2 BET: 1495 m^2g^{-1}, O_2 BET: 2091 m^2g^{-1}	0.27	19200		No (0-<5%)	[104]
$Ru(iphen)_3$ linker doped into MAF-34		O_2 uptake: 3.2 cm^3g^{-1}	0.25	230		No (0-<5%)	[101]
MIL-100(In)⊃Tb^{3+}<br>CPM-5⊃TB^{3+}	on ITO glass	Pore free inner diameter: 20 & 26 Å<br>pore size: 4.89 Å	0.07<br>0.01	0.08<br>0.01	6 / 53<br>90 / 60	Yes b)	[96]
MAF-2	-	N_2 BET: 539 m^2g^{-1}	3.56	30	<1	Yes	[113]

	silicone rubber		2.64	20		No<br>(0-10%)	
Pyrene @ MAF-4	on silica silicone rubber	Cavities:<br>Ø~11.4 Å,<br>apertures ~ 3.3 Å	0.18<br>0.07	3230<br>1320		Yes	[105]
$Pb_4([Ir(ppy)_2(4\text{-}dcbpy)]PF_6)_4I_4(DMF)_2]\cdot 10H_2O$			0.05	350	50 / 24	Yes	[102]
C480 @ $Pb_4([Ir(ppy)_2(4\text{-}dcbpy)]PF_6)_4I_4(DMF)_2]\cdot 10H_2O$	PDMS		0.04	260			
AlQ_3 @ MAF-X10		N_2 BET:<br>>2000 m^2g^{-1},<br>aperture size:<br>5.8·6.6 Å,<br>cavity size:<br>9.4·9.9 Å	0.08	2030		Yes	[106]
MAF-X10			0.05	15100			
MCF-53 ($Tb_{0.966}/Eu_{0.034}$) (Eu^{3+} emission)		Void ratio 15.5%,<br>Channel size<br>3.8 Å	0.10	0.3		No<br>(0-10%)	[103]
MCF-53 ($Tb_{0.786}/Eu_{0.214}$) (Eu^{3+} emission)			0.09	0.4			
MCF-53 ($Tb_{0.663}/Eu_{0.337}$) (Eu^{3+} emission)			0.06	0.3			
MCF-53 (Eu) (Eu^{3+} emission)			0.01	0.2			
MCF-53 (Tb) (ligand emission)			0.03	21700			
MCF-53 ($Tb_{0.966}/Eu_{0.034}$) (ligand emission)			0.009	8020			
MCF-53 ($Tb_{0.786}/Eu_{0.214}$) (ligand emission)			0.01	10500			

MCF-53 ($Tb_{0.663}/Eu_{0.337}$) (ligand emission)			0.01	18300			
MCF-53 (Eu) (ligand emission)			0.002	3300			
Gd[$(Pyr)_4$cyclen] @ ZIF-8		N_2 BET: 806.12 m^2g^{-1}	4.64	130		No (0-2%)	[107]
[$Ag_{12}(StBu)_8(CF3COO)_4(bpy\text{-}NH_2)_4]_n$		Void space 17.5%	2250	720	0.3	No (0-<1%)	[110]
PtCN(L) @ ZIF-8 [a)]		BET 1378 m^2g^{-1}, total pore vol. 0.74 cm^3g^{-1}, µpore vol. 0.4 cm^3g^{-1}	0.21	10			[108]
EuNDC		Pore sizes: 8 Å & 4 Å, N_2 BET: 417 m^2g^{-1}	0.13	0.1	10 / 70		[111]
EuNDC	PDMS	N_2 BET: 6.05 m^2g^{-1}	0.04	0.03			
SUMOF-6-EU@O-PP	PDMS	N_2 BET: 524 m^2g^{-1}	0.07	0.06	10 / 60	Yes[b)]	[112]
PCN-224			0.25	37300	6 / 7	No (0-20%)	[97]
	Glass fibers		0.15	22400		No (0-<10%)	
	PAN fibers		0.19	28400	<1	No (0-20%)	
	Silica gel		0.29	43300		No (0-<10%)	
H_2TMCPP	pTMSP		0.07	7200			
	Silica gl		0.19	19000			
Pt-PCN-224			73	3900		No (0-<0.05%)	
Pd-PCN-224			2610	6700		No (0-<0.01%)	
Ir$(ppy)_2(BisPh_2PPh)$@ bio-MOF-1		N_2 BET: 561 m^2g^{-1},	0.22	50	9 / 22	Yes	[109]

$Ir(ppy)_2(PPh_3)$@bio-MOF-1		pore size 17.1 Å<br>N_2 BET: 672m^{2}g^{-1},<br>pore size 19.5 Å	0.20	80			

a) τ_0 not given for the indicator in this material but estimated based on other literature

b) first calibration point at 20% O_2

Table S8. *Overview of alumina-based oxygen sensing materials*

Indicator	Matrix	Porosity	K_{SV} / kPa^{-1}	k_q / $Pa^{-1}s^{-1}$	Response / Recovery time / s	Linearity of SV plot	Ref
H_2TMCUPP [a)]<br>H_2MCUTPP [a)]<br>H_2CTPP [a)]<br>PtTMCUPP [a)]<br>PtMCUTPP [a)]<br>PtCTPP [a)]	Anodized alumina plate	apd = 150 – 200 Å	0.23<br>0.14<br>0.14<br>17<br>10<br>16	23000<br>14900<br>14100<br>210<br>130<br>200	9.4 / 15.5<br>12.5 / 17.0<br>9.6 / 20.8<br>5.0 / 667<br>8.9 / 590<br>4.6 / 580	No (0-<20%)<br>No (0-<5%)	[124]
Ru(dpp)$_3$<br>H_2TCPP<br>PtTCPP<br>PBA	Anodized alumina	apd = 200-1000 Å Thickness 6.1 µm	0.29<br>0.08<br>7.17<br>0.22	220<br>12400<br>160<br>4300	$2\cdot10^{-5}$<br>$1\cdot10^{-5}$<br><br>$<1\cdot10^{-3}$	No (0-<10%)	[123]
PdPC$_{10}$COOH [a)]<br>H_2TCPP [a)]<br>PtTCPP [a)]<br>PdTCPP [a)]<br>Tb(acac)$_3$phen	Alumina TLC plate		3.0<br>0.35<br>1.6<br>4.5<br>0.14	3<br>35300<br>20<br>5<br>0.6	36 / 148<br>3.4 / 15<br>8.6 / 50<br>36 / 148<br>7.3 / 70	No (0-<10%)	[126 – 128]
Ru(dC$_8$pp)$_3$ [a)]<br>[Ir(ppy)$_2$(Me$_4$dabpy)]ClO$_3$ [a)]<br>[Ir(ppy)$_2$(SCN)$_2$]N(C$_4$H$_9$)$_4$ [a)]<br>[Ir(ppy)$_2$(OCN)$_2$]N(C$_4$H$_9$)$_4$ [a)]	AlOOH in PVA	Total pore vol. 20 mL m^{-2}, apd 190 Å	0.71<br>1.0<br>0.35<br>0.87	140<br>510<br>180<br>440	<1 / <2	No (0-<20%)	[129]
Ru(dC$_8$pp)$_3$ [a)]<br>[Ir(ppy)$_2$(Me$_4$dabpy)]ClO$_3$ [a)]<br>[Ir(ppy)$_2$(SCN)$_2$]N(C$_4$H$_9$)$_4$ [a)]<br>[Ir(ppy)$_2$(OCN)$_2$]N(C$_4$H$_9$)$_4$ [a)]	PS		0.016<br>0.012<br>0.0030<br>0.0070	3<br>6<br>2<br>4			
[Ir(F$_2$ppy)$_2$(Me$_4$dabpy)]PF$_6$<br>[Ir(ppy)$_2$(dpp)]PF$_6$<br>[Ir(F$_2$ppy)$_2$(dpp)]PF$_6$<br>[Ir(ppy)$_2$(Brbpy)]PF$_6$<br>[Ir(F$_2$ppy)$_2$(Me$_4$dabpy)]PF$_6$	PS		3.6<br>1.6<br>0.72<br>3.1<br>0.87	1790<br>820<br>360<br>1600<br>440	<4	No (0-<10%)	[130]

[Ir(ppy)$_2$(dpp)]PF$_6$	PS		2.0	980			
[Ir(F$_2$ppy)$_2$(dpp)]PF$_6$	(8.5 wt.%		0.34	170			
[Ir(ppy)$_2$(Brbpy)]PF$_6$	plasticizer)		1.0	520			
[Ir(F$_2$ppy)$_2$(Me$_4$dabpy)]PF$_6$	PS		0.53	270			
[Ir(ppy)$_2$(dpp)]PF$_6$	(17.5 wt.%		0.96	480			
[Ir(F$_2$ppy)$_2$(dpp)]PF$_6$	plasticizer)		0.28	140			
[Ir(ppy)$_2$(Brbpy)]PF$_6$			1.1	570			
[Ir(F$_2$ppy)$_2$(Me$_4$dabpy)]PF$_6$	AlOOH		12	6200			
[Ir(ppy)$_2$(dpp)]PF$_6$			28	14200			
[Ir(F$_2$ppy)$_2$(dpp)]PF$_6$			2.2	1100			
[Ir(ppy)$_2$(Brbpy)]PF$_6$			5.6	2800			

a) τ_0 not given for the indicator in this material but estimated based on other literature

b) first calibration point at 20% O_2

Table S9. *Overview of miscellaneous (porous) oxygen sensitive materials*

**Indicator**	**Matrix**	**Porosity**	**K_{SV} / kPa^{-1}**	**k_q / $Pa^{-1}s^{-1}$**	**Response / Recovery time / s**	**Linearity of SV plot**	**Ref**
$Ir(ppy)_3$ [a)]	Amberlite XAD-4	750 m^2g^{-1}, apd 100 Å	0.34	170		No (0-<10%)	[117]
PtTFPP-MA [a)]<br>Covalently grafted to matrix	PS-PVP	apd 4000 Å, “high” pore density<br>apd 4000 Å, “low” pore density<br>Non-porous	0.6<br>0.4<br>0.2	0.8<br>0.5<br>0.3	2.5 / 3<br>3.5 / 5.5<br>4 / 6.5	Yes	[118]
PtBP	PP fibers hollow, 40 µm diameter<br>PE fibers solid, 40 µm diameter		0.39<br>0.53	10<br>13	60<br>30	No (0-<10%)	[120]
PtOEP [a)]<br><br><br>PdOEP [a)]	PS<br>PDMS<br>pTMSP<br>PS<br>PDMS<br>pTMSP		0.13<br>0.20<br>6.5<br>0.84<br>0.89<br>17	1<br>2<br>70<br>0.8<br>0.9<br>20	35 / 100<br>10 / 82<br>3.6 / 73.2<br>10.6 / 158.8<br>9 / 200.2<br>3.2 / 250	Yes<br>No (0-<20%<br>No (0-<6%)	[157,158]
PtTFPP	pTMSP				<0.03	No	[159]
$Ru(bpy)_3$	TiO_2 sol-gl	Average porosity 60%, thickness 9 µm	1.9	4600	4 / 7	No (0-<10%)	[160]
PtTPP [a)]<br>linked to 3-(trimethoxysilyl)propyl methacrylate and	PDMS micropillar arrays	Micropillar height: 60 µm, diameter:	1.0	10	25 / 97	Yes	[119]

covalently grafted to matrix	PDMS film	50 µm, distance: 100 µm	0.16	2			
$[Ru(phen)_3]$tfpb<br>$[Ru(5,6\text{-}Me_2phen)]$tfpb<br>$[Ru(phen)_3](PF_6)_2$	Pure dye crystal	Void fraction 0.055<br><br>Void fraction 0.01	0.024<br>0.0079<br>0.033	40<br>10<br>70		No	[161–163]
$Ru(bpy)_3$ [a)]	Zeolite Y in E4 silicone (Encapsulated in zeolite cavities)<br>Zeolite Y in E4 silicone (adsorbed on zeolite surface)<br>Silica (Partisil 5) in E4 silicone	Zeolite Y: Al/Si ratio = 11, dealuminated with EDTA Main channel diameter: 7.4 Å, supercage diameter: 13 Å<br>350 m^2g^{-1}, 85 Å apd	0.024<br>0.0069<br>0.032	20<br>7<br>30	6-10	No (0-<20%)	[164]

a) τ_0 not given for the indicator in this material but estimated based on other literature

b) first calibration point at 20% O_2

a) Estimates for τ_0 when not given in the paper

$Ru(bpy)_3$ and derivatives thereof	1 µs
$Ru(phen)_3$ and derivatives thereof	2.5 µs
$Ru(dpp)_3$ and derivatives thereof	5 µs
PtOEP and derivatives thereof	100 µs
PtTPP and derivatives thereof	80 µs
Pd porphyrins	1 ms

$Ir(ppy)_3$ and derivatives thereof	2 µs

4. References

[1] DeGraff BA, Demas JN. Luminescence-Based Oxygen Sensors. In: Geddes CD, Lakowicz JR, editors. Reviews in Fluorescence 2005, vol. 2005, Boston, MA: Springer US; 2005, p. 125–51. https://doi.org/10.1007/0-387-23690-2_6.

[2] Wang X, Wolfbeis OS. Optical methods for sensing and imaging oxygen: materials, spectroscopies and applications. Chem Soc Rev 2014;43:3666–761. https://doi.org/10.1039/C4CS00039K.

[3] Moßhammer M, Strobl M, Kühl M, Klimant I, Borisov SM, Koren K. Design and Application of an Optical Sensor for Simultaneous Imaging of pH and Dissolved O2 with Low Cross-Talk. ACS Sensors 2016;1:681–7. https://doi.org/10.1021/acssensors.6b00071.

[4] Koop-Jakobsen K, Mueller P, Meier RJ, Liebsch G, Jensen K. Plant-Sediment Interactions in Salt Marshes – An Optode Imaging Study of O2, pH, and CO2 Gradients in the Rhizosphere. Frontiers in Plant Science 2018;9. https://doi.org/10.3389/fpls.2018.00541.

[5] Nielsen SD, Paegle I, Borisov SM, Kjeldsen KU, Røy H, Skibsted J, et al. Optical Sensing of pH and O2 in the Evaluation of Bioactive Self-Healing Cement. ACS Omega 2019;4:20237–43. https://doi.org/10.1021/acsomega.9b02541.

[6] Wolfbeis OS. Luminescent sensing and imaging of oxygen: Fierce competition to the Clark electrode. BioEssays 2015;37:921–8. https://doi.org/10.1002/bies.201500002.

[7] Quaranta M, Borisov SM, Klimant I. Indicators for optical oxygen sensors. Bioanal Rev 2012;4:115–57. https://doi.org/10.1007/s12566-012-0032-y.

[8] Schweitzer C, Schmidt R. Physical Mechanisms of Generation and Deactivation of Singlet Oxygen. Chem Rev 2003;103:1685–758. https://doi.org/10.1021/cr010371d.

[9] Borisov SM. CHAPTER 1. Fundamentals of Quenched Phosphorescence O2 Sensing and Rational Design of Sensor Materials. In: Papkovsky DB, Dmitriev RI, editors. Detection Science, Cambridge: Royal Society of Chemistry; 2018, p. 1–18. https://doi.org/10.1039/9781788013451-00001.

[10] Valeur B, Wiley InterScience (Online service). Molecular fluorescence: principles and applications. New York: Wiley-VCH; 2001.

[11] Lakowicz JR. Principles of fluorescence spectroscopy. 3rd ed. New York: Springer; 2006.

[12] McDonagh C, Bowe P, Mongey K, MacCraith BD. Characterisation of porosity and sensor response times of sol–gel-derived thin films for oxygen sensor applications. J Non Cryst Solids 2002;306:138–48. https://doi.org/10.1016/S0022-3093(02)01154-7.

[13] Han B-H, Manners I, Winnik MA. Oxygen Sensors Based on Mesoporous Silica Particles on Layer-by-Layer Self-assembled Films. Chem Mater 2005;17:3160–71. https://doi.org/10.1021/cm047770k.

[14] Djurovich PI, Murphy D, Thompson ME, Hernandez B, Gao R, Hunt PL, et al. Cyclometalated iridium and platinum complexes as singlet oxygen photosensitizers: quantum yields, quenching rates and correlation with electronic structures. Dalton Trans 2007:3763. https://doi.org/10.1039/b704595f.

[15] Lehrer S. Solute perturbation of protein fluorescence. Quenching of the tryptophyl fluorescence of model compounds and of lysozyme by iodide ion. Biochemistry 1971;10:3254–63. https://doi.org/10.1021/bi00793a015.

[16] Carraway ER, Demas JN, DeGraff BA. Photophysics and oxygen quenching of transition-metal complexes on fumed silica. Langmuir 1991;7:2991–8. https://doi.org/10.1021/la00060a015.

[17] Okada T, Yoshido S, Miura H, Yamakami T, Sakai T, Mishima S. Swellable Microsphere of a Layered Silicate Produced by Using Monodispersed Silica Particles. J Phys Chem C 2012;116:21864–9. https://doi.org/10.1021/jp307108t.

[18] Kruk M, Jaroniec M, Sakamoto Y, Terasaki O, Ryoo R, Ko CH. Determination of Pore Size and Pore Wall Structure of MCM-41 by Using Nitrogen Adsorption, Transmission Electron Microscopy, and X-ray Diffraction. J Phys Chem B 2000;104:292–301. https://doi.org/10.1021/jp992718a.

[19] Koren K, Borisov SM, Klimant I. Stable optical oxygen sensing materials based on click-coupling of fluorinated platinum(II) and palladium(II) porphyrins—A convenient way to eliminate dye migration and leaching. Sens Actuators B Chem 2012;169:173–81. https://doi.org/10.1016/j.snb.2012.04.062.

[20] Yun S, Luo H, Gao Y. Superhydrophobic silica aerogel microspheres from methyltrimethoxysilane: rapid synthesis via ambient pressure drying and excellent absorption properties. RSC Adv 2014;4:4535–42. https://doi.org/10.1039/C3RA46911E.

[21] Kautsky H. Quenching of luminescence by oxygen. Trans Faraday Soc 1939;35:216. https://doi.org/10.1039/tf9393500216.

[22] Twarowski AJ, Good Lisa. Phosphorescence quenching by molecular oxygen: zinc tetraphenylporphin on solid supports. J Phys Chem 1987;91:5252–7. https://doi.org/10.1021/j100304a024.

[23] Wolfbeis OS, Leiner MJP, Posch HE. A new sensing material for optical oxygen measurement, with the indicator embedded in an aqueous phase. Mikrochim Acta 1986;90:359–66. https://doi.org/10.1007/BF01199278.

[24] Krasnansky R, Koike K, Thomas JK. Gaussian approximation to the unique heterogeneous Langmuir-Hinshelwood type fluorescence quenching at the silica gel gas/solid interface: pyrene and 9,10-diphenylanthracene singlet quenching by oxygen. J Phys Chem 1990;94:4521–8. https://doi.org/10.1021/j100374a033.

[25] Hartmann P, Leiner MJP, Lippitsch ME. Response characteristics of luminescent oxygen sensors. Sens Actuators B Chem 1995;29:251–7. https://doi.org/10.1016/0925-4005(95)01691-0.

[26] Posch HE, Wolfbeis OS. Optical sensors, 13: fibre-optic humidity sensor based on fluorescence quenching. Sens Actuators 1988;15:77–83. https://doi.org/10.1016/0250-6874(88)85019-4.

[27] He H, Fraatz RJ, Leiner MJP, Rehn MM, Tusa JK. Selection of silicone polymer matrix for optical gas sensing. Sens Actuators B Chem 1995;29:246–50. https://doi.org/10.1016/0925-4005(95)01690-2.

[28] Klimant I, Belser P, Wolfbeis OS. Novel metal—organic ruthenium(II) diimin complexes for use as longwave excitable luminescent oxygen probes. Talanta 1994;41:985–91. https://doi.org/10.1016/0039-9140(94)E0051-R.

[29] Mingoarranz et al. FJ, Moreno-Bondi MC, García-Fresnadillo D, de Dios C, Orellana G. Oxygen-sensitive layers for optical fibre devices. Mikrochim Acta 1995;121:107–18. https://doi.org/10.1007/BF01248245.

[30] Koren K, Borisov SM, Saf R, Klimant I. Strongly Phosphorescent Iridium(III)-Porphyrins - New Oxygen Indicators with Tuneable Photophysical Properties and Functionalities. Eur J Inorg Chem 2011;2011:1531–4. https://doi.org/10.1002/ejic.201100089.

[31] Badia R, Marta E. Diaz-Garcia, Garcia-Fresnadillo A. A sensitive probe for oxygen sensing in gas mixtures, based on room-temperature phosphorescence quenching. Mikrochim Acta 1995;121:51–61. https://doi.org/10.1007/BF01248240.

[32] Borisov SM, Lehner P, Klimant I. Novel optical trace oxygen sensors based on platinum(II) and palladium(II) complexes with 5,10,15,20-meso-tetrakis-(2,3,4,5,6-pentafluorphenyl)-porphyrin covalently immobilized on silica-gel particles. Anal Chim Acta 2011;690:108–15. https://doi.org/10.1016/j.aca.2011.01.057.

[33] Melnikov PV, Naumova AO, Alexandrovskaya AYu, Zaitsev NK. Optimizing Production Conditions for a Composite Optical Oxygen Sensor Using Mesoporous SiO2. Nanotechnol Russia 2018;13:602–8. https://doi.org/10.1134/S1995078018060083.

[34] Xu H, Aylott JW, Kopelman R, Miller TJ, Philbert MA. A Real-Time Ratiometric Method for the Determination of Molecular Oxygen Inside Living Cells Using Sol−Gel-Based Spherical Optical Nanosensors with Applications to Rat C6 Glioma. Anal Chem 2001;73:4124–33. https://doi.org/10.1021/ac0102718.

[35] Lei B, Li B, Zhang H, Lu S, Zheng Z, Li W, et al. Mesostructured Silica Chemically Doped with RuII as a Superior Optical Oxygen Sensor. Adv Funct Mater 2006;16:1883–91. https://doi.org/10.1002/adfm.200500737.

[36] Lei B, Li B, Zhang H, Zhang L, Li W. Synthesis, Characterization, and Oxygen Sensing Properties of Functionalized Mesoporous SBA-15 and MCM-41 with a Covalently Linked Ruthenium(II) Complex. J Phys Chem C 2007;111:11291–301. https://doi.org/10.1021/jp070008w.

[37] Wu X, Song L, Li B, Liu Y. Synthesis, characterization, and oxygen sensing properties of Ru(II) complex covalently grafted to mesoporous MCM-41. J Lumin 2010;130:374–9. https://doi.org/10.1016/j.jlumin.2009.09.023.

[38] Zhang H, Sun Y, Ye K, Zhang P, Wang Y. Oxygen sensing materials based on mesoporous silica MCM-41 and Pt(ii)–porphyrin complexes. J Mater Chem 2005;15:3181. https://doi.org/10.1039/b503336e.

[39] Wang B, Liu Y, Li B, Yue S, Li W. Optical oxygen sensing materials based on trinuclear starburst ruthenium(II) complexes assembled in mesoporous silica. J Lumin 2008;128:341–7. https://doi.org/10.1016/j.jlumin.2007.08.011.

[40] Shi L, Li B. A Series of Cu(I) Complexes Containing 1,10-Phenanthroline Derivative Ligands: Synthesis, Characterization, Photophysical, and Oxygen-Sensing Properties. Eur J Inorg Chem 2009;2009:2294–302. https://doi.org/10.1002/ejic.200900123.

[41] Liu Y, Li B, Cong Y, Zhang L, Fan D, Shi L. Optical oxygen sensing materials based on a novel dirhenium(I) complex assembled in mesoporous silica. J Lumin 2011;131:781–5. https://doi.org/10.1016/j.jlumin.2010.12.003.

[42] Haitao J, Huilin Y, Fan L, Yang L. Fabrication and performances of an optical sensor system constructed by a novel Cu(I) complex embedded on silica matrix. J Lumin 2012;132:198–204. https://doi.org/10.1016/j.jlumin.2011.08.023.

[43] Wang B, Zhang L, Li B, Li Y, Shi Y, Shi T. Synthesis, characterization, and oxygen sensing properties of functionalized mesoporous silica SBA-15 and MCM-41 with a Pt(II)–porphyrin complex. Sens Actuators B Chem 2014;190:93–100. https://doi.org/10.1016/j.snb.2013.08.036.

[44] Lobnik A, Korent Urek Š, Turel M, Frančič N. Sol-gel based optical chemical sensors. In: Baldini F, Homola J, Lieberman RA, Kalli K, editors., Prague, Czech Republic: 2011, p. 80730V. https://doi.org/10.1117/12.886819.

[45] MacCraith BD, McDonagh CM, O'Keeffe G, Keyes ET, Vos JG, O'Kelly B, et al. Fibre optic oxygen sensor based on fluorescence quenching of evanescent-wave excited ruthenium complexes in sol–gel derived porous coatings. Analyst 1993;118:385–8. https://doi.org/10.1039/AN9931800385.

[46] McEvoy AK, McDonagh CM, MacCraith BD. Dissolved oxygen sensor based on fluorescence quenching of oxygen-sensitive ruthenium complexes immobilized in sol–gel-derived porous silica coatings. Analyst 1996;121:785–8. https://doi.org/10.1039/AN9962100785.

[47] Mcevoy AK, Mcdonagh C, Maccraith BD. Optimisation of sol-gel-derived silica films for optical oxygen sensing. J Sol-Gel Sci Technol 1997;8:1121–5. https://doi.org/10.1007/BF02436994.

[48] Lee S-K, Okura I. Porphyrin-doped sol-gel glass as a probe for oxygen sensing. Anal Chim Acta 1997;342:181–8. https://doi.org/10.1016/S0003-2670(96)00562-4.

[49] Lee S-K, Okura I. Optical Sensor for Oxygen Using a Porphyrin-doped Sol–Gel Glass. Analyst 1997;122:81–4. https://doi.org/10.1039/a604885d.

[50] McDonagh C, MacCraith BD, McEvoy AK. Tailoring of Sol−Gel Films for Optical Sensing of Oxygen in Gas and Aqueous Phase. Anal Chem 1998;70:45–50. https://doi.org/10.1021/ac970461b.

[51] Murtagh MT, Shahriari MR, Krihak M. A Study of the Effects of Organic Modification and Processing Technique on the Luminescence Quenching Behavior of Sol−Gel Oxygen Sensors Based on a Ru(II) Complex. Chem Mater 1998;10:3862–9. https://doi.org/10.1021/cm9802806.

[52] Tao Z, Tehan EC, Tang Y, Bright FV. Stable Sensors with Tunable Sensitivities Based on Class II Xerogels. Anal Chem 2006;78:1939–45. https://doi.org/10.1021/ac051657b.

[53] Tang Y, Tehan EC, Tao Z, Bright FV. Sol−Gel-Derived Sensor Materials That Yield Linear Calibration Plots, High Sensitivity, and Long-Term Stability. Anal Chem 2003;75:2407–13. https://doi.org/10.1021/ac030087h.

[54] Yeh T-S, Chu C-S, Lo Y-L. Highly sensitive optical fiber oxygen sensor using Pt(II) complex embedded in sol–gel matrices. Sens Actuators B Chem 2006;119:701–7. https://doi.org/10.1016/j.snb.2006.01.051.

[55] Amao Y, Asai K, Miyashita T, Okura I. Novel optical oxygen sensing material: platinum porphyrin-fluoropolymer film. Polym Adv Technol 2000;11:705–9. https://doi.org/10.1002/1099-1581(200008/12)11:8/12<705::AID-PAT23>3.0.CO;2-L.

[56] Amao Y, Miyashita T, Okura I. Optical oxygen sensing based on the luminescence change of metalloporphyrins immobilized in styrene–pentafluorostyrene copolymer film. Analyst 2000;125:871–5. https://doi.org/10.1039/b000702l.

[57] Amao Y, Ishikawa Y, Okura I. Green luminescent iridium(III) complex immobilized in fluoropolymer film as optical oxygen-sensing material. Anal Chim Acta 2001;445:177–82. https://doi.org/10.1016/S0003-2670(01)01254-5.

[58] Lehner P, Larndorfer C, Garcia-Robledo E, Larsen M, Borisov SM, Revsbech N-P, et al. LUMOS - A Sensitive and Reliable Optode System for Measuring Dissolved Oxygen in the Nanomolar Range. PLoS ONE 2015;10:e0128125. https://doi.org/10.1371/journal.pone.0128125.

[59] Higgins C, Wencel D, Burke CS, MacCraith BD, McDonagh C. Novel hybrid optical sensor materials for in-breath O2 analysis. Analyst 2008;133:241–7. https://doi.org/10.1039/B716197B.

[60] Estella J, Wencel D, Moore JP, Sourdaine M, McDonagh C. Fabrication and performance evaluation of highly sensitive hybrid sol–gel-derived oxygen sensor films based on a fluorinated precursor. Anal Chim Acta 2010;666:83–90. https://doi.org/10.1016/j.aca.2010.03.053.

[61] Bukowski RM, Ciriminna R, Pagliaro M, Bright FV. High-Performance Quenchometric Oxygen Sensors Based on Fluorinated Xerogels Doped with [Ru(dpp) 3] 2+. Anal Chem 2005;77:2670–2. https://doi.org/10.1021/ac048199b.

[62] Bukowski RM, Davenport MD, Titus AH, Bright FV. O2 -Responsive Chemical Sensors Based on Hybrid Xerogels That Contain Fluorinated Precursors. Appl Spectrosc 2006;60:951–7. https://doi.org/10.1366/000370206778397489.

[63] Chu C-S, Lo Y-L. High-performance fiber-optic oxygen sensors based on fluorinated xerogels doped with Pt(II) complexes. Sens Actuators B Chem 2007;124:376–82. https://doi.org/10.1016/j.snb.2006.12.049.

[64] Chu C-S, Lo Y-L. Highly sensitive and linear calibration optical fiber oxygen sensor based on Pt(II) complex embedded in sol–gel matrix. Sens Actuators B Chem 2011;155:53–7. https://doi.org/10.1016/j.snb.2010.11.023.

[65] Ciriminna R, Pagliaro M. Organofluoro-silica xerogels as high-performance optical oxygen sensors. Analyst 2009;134:1531. https://doi.org/10.1039/b819417c.

[66] Leventis N, Elder IA, Rolison DR, Anderson ML, Merzbacher CI. Durable Modification of Silica Aerogel Monoliths with Fluorescent 2,7-Diazapyrenium Moieties. Sensing Oxygen near the Speed of Open-Air Diffusion. Chem Mater 1999;11:2837–45. https://doi.org/10.1021/cm9901966.

[67] Leventis N, Rawashdeh A-MM, Elder IA, Yang J, Dass A, Sotiriou-Leventis C. Synthesis and Characterization of Ru(II) Tris(1,10-phenanthroline)-Electron Acceptor Dyads Incorporating the 4-Benzoyl- N -methylpyridinium Cation or N -Benzyl- N ‘-methyl Viologen. Improving the Dynamic Range, Sensitivity, and Response Time of Sol−Gel-Based Optical Oxygen Sensors. Chem Mater 2004;16:1493–506. https://doi.org/10.1021/cm034999b.

[68] Plata DL, Briones YJ, Wolfe RL, Carroll MK, Bakrania SD, Mandel SG, et al. Aerogel-platform optical sensors for oxygen gas. J Non Cryst Solids 2004;350:326–35. https://doi.org/10.1016/j.jnoncrysol.2004.06.046.

[69] Imran M, Motta N, Shafiei M. Electrospun one-dimensional nanostructures: a new horizon for gas sensing materials. Beilstein J Nanotechnol 2018;9:2128–70. https://doi.org/10.3762/bjnano.9.202.

[70] Rivero P, Goicoechea J, Arregui F. Optical Fiber Sensors Based on Polymeric Sensitive Coatings. Polymers 2018;10:280. https://doi.org/10.3390/polym10030280.

[71] George G, Luo Z. A Review on Electrospun Luminescent Nanofibers: Photoluminescence Characteristics and Potential Applications. CNANO 2020;16:321–62. https://doi.org/10.2174/1573413715666190112121113.

[72] Wang Y, Li B, Liu Y, Zhang L, Zuo Q, Shi L, et al. Highly sensitive oxygen sensors based on Cu(i) complex–polystyrene composite nanofibrous membranes prepared by electrospinning. Chem Commun 2009:5868. https://doi.org/10.1039/b910305h.

[73] Wang L-Y, Xu Y, Lin Z, Zhao N, Xu Y. Electrospinning fabrication and oxygen sensing properties of Cu(I) complex–polystyrene composite microfibrous membranes. JLumin 2011;131:1277–82. https://doi.org/10.1016/j.jlumin.2011.03.017.

[74] Wang Y, Li B, Zhang L, Zuo Q, Li P, Zhang J, et al. High-Performance Oxygen Sensors Based on EuIII Complex/Polystyrene Composite Nanofibrous Membranes Prepared by Electrospinning. ChemPhysChem 2011;12:349–55. https://doi.org/10.1002/cphc.201000884.

[75] Yingkui L. High performance oxygen sensing nanofibrous membranes of Eu(III) complex/polystyrene prepared by electrospinning. Spectrochimica Acta Part A: Molecular and Biomolecular Spectroscopy 2011;79:356–60. https://doi.org/10.1016/j.saa.2011.03.012.

[76] Wolf C, Tscherner M, Köstler S, Ribitsch V. Optochemical sensors based on polymer nanofibers with ultra-fast response characteristics. 2014 IEEE SENSORS, 2014, p. 950–3. https://doi.org/10.1109/ICSENS.2014.6985159.

[77] Wolf C, Tscherner M, Köstler S. Ultra-fast opto-chemical sensors by using electrospun nanofibers as sensing layers. Sens Actuators B Chem 2015;209:1064–9. https://doi.org/10.1016/j.snb.2014.11.070.

[78] Kai R, Jun W, Huali J. Electrospinning fibrous films doped with iridium complexes for high performance oxygen sensing: Synthesis and characterization. Sens Actuators B Chem 2017;240:697–708. https://doi.org/10.1016/j.snb.2016.09.033.

[79] Lee S-K, Okura I. Photoluminescent determination of oxygen using metalloporphyrin-polymer sensing systems. Spectrochimica Acta Part A: Molecular and Biomolecular Spectroscopy 1998;54:91–100. https://doi.org/10.1016/S1386-1425(97)00206-0.

[80] Borisov SM, Zenkl G, Klimant I. Phosphorescent Platinum(II) and Palladium(II) Complexes with Azatetrabenzoporphyrins—New Red Laser Diode-Compatible Indicators for Optical Oxygen Sensing. ACS Appl Mater Interfaces 2010;2:366–74. https://doi.org/10.1021/am900932z.

[81] Payne SJ, Fiore GL, Fraser CL, Demas JN. Luminescence Oxygen Sensor Based on a Ruthenium(II) Star Polymer Complex. Anal Chem 2010;82:917–21. https://doi.org/10.1021/ac9020837.

[82] Tian Y, Shumway BR, Gao W, Youngbull C, Holl MR, Johnson RH, et al. Influence of matrices on oxygen sensing of three sensing films with chemically conjugated platinum porphyrin probes and preliminary application for monitoring of oxygen consumption of Escherichia coli (E. coli). Sens Actuators B Chem 2010;150:579–87. https://doi.org/10.1016/j.snb.2010.08.036.

[83] Koren K, Hutter L, Enko B, Pein A, Borisov SM, Klimant I. Tuning the dynamic range and sensitivity of optical oxygen-sensors by employing differently substituted polystyrene-derivatives. Sens Actuators B Chem 2013;176:344–50. https://doi.org/10.1016/j.snb.2012.09.057.

[84] Lee S, Park J-W. Luminescent oxygen sensors with highly improved sensitivity based on a porous sensing film with increased oxygen accessibility and photoluminescence. Sens Actuators B Chem 2017;249:364–77. https://doi.org/10.1016/j.snb.2017.04.112.

[85] Yang X, Li L, Yuan L, Li S, Luo S, Liu Y, et al. Submicrometer organic silica gel fiber for oxygen sensing. Opt Lett 2011;36:4656. https://doi.org/10.1364/OL.36.004656.

[86] Xue R, Behera P, Viapiano MS, Lannutti JJ. Rapid response oxygen-sensing nanofibers. Mat Sci and Eng C 2013;33:3450–7. https://doi.org/10.1016/j.msec.2013.04.030.

[87] Xue R, Behera P, Xu J, Viapiano MS, Lannutti JJ. Polydimethylsiloxane core–polycaprolactone shell nanofibers as biocompatible, real-time oxygen sensors. Sens Actuators B Chem 2014;192:697–707. https://doi.org/10.1016/j.snb.2013.10.084.

[88] Presley K, Hwang J, Cheong S, Tilley R, Collins J, Viapiano M, et al. Nanoscale upconversion for oxygen sensing. Mat Sci and Eng C 2017;70:76–84. https://doi.org/10.1016/j.msec.2016.08.056.

[89] Akram M, Mei Z, Shi J, Wen J, Khalid H, Jiang J, et al. Electrospun nanofibers and spin coated films prepared from side-chain copolymers with chemically bounded platinum (II) porphyrin moieties for oxygen sensing and pressure sensitive paints. Talanta 2018;188:124–34. https://doi.org/10.1016/j.talanta.2018.05.067.

[90] Rowsell JLC, Yaghi OM. Metal–organic frameworks: a new class of porous materials. Microporous and Mesoporous Materials 2004;73:3–14. https://doi.org/10.1016/j.micromeso.2004.03.034.

[91] Lu W, Wei Z, Gu Z-Y, Liu T-F, Park J, Park J, et al. Tuning the structure and function of metal–organic frameworks via linker design. Chem Soc Rev 2014;43:5561–93. https://doi.org/10.1039/C4CS00003J.

[92] Zhang Y, Yuan S, Day G, Wang X, Yang X, Zhou H-C. Luminescent sensors based on metal-organic frameworks. Coordination Chemistry Reviews 2018;354:28–45. https://doi.org/10.1016/j.ccr.2017.06.007.

[93] Ni J, Wei K-J, Min Y, Chen Y, Zhan S, Li D, et al. Copper(i) coordination polymers of 2,2′-dipyridylamine derivatives: syntheses, structures, and luminescence. Dalton Trans 2012;41:5280. https://doi.org/10.1039/c2dt12032a.

[94] Tang Y-Y, Ding C-X, Ng S-W, Xie Y-S. Syntheses, structures and photoluminescence of Zn(ii), Ag(i), Cu(i) and Co(ii) coordination polymers of a tetrapyridyl ligand. RSC Adv 2013;3:18134. https://doi.org/10.1039/c3ra43405b.

[95] Cui Y, Chen B, Qian G. Lanthanide metal-organic frameworks for luminescent sensing and light-emitting applications. Coordination Chemistry Reviews 2014;273–274:76–86. https://doi.org/10.1016/j.ccr.2013.10.023.

[96] Dou Z, Yu J, Cui Y, Yang Y, Wang Z, Yang D, et al. Luminescent Metal–Organic Framework Films As Highly Sensitive and Fast-Response Oxygen Sensors. J Am Chem Soc 2014;136:5527–30. https://doi.org/10.1021/ja411224j.

[97] Burger T, Winkler C, Dalfen I, Slugovc C, Borisov SM. Porphyrin based metal–organic frameworks: highly sensitive materials for optical sensing of oxygen in gas phase. J Mater Chem C 2021:10.1039.D1TC03735H. https://doi.org/10.1039/D1TC03735H.

[98] Xie Z, Ma L, deKrafft KE, Jin A, Lin W. Porous Phosphorescent Coordination Polymers for Oxygen Sensing. J Am Chem Soc 2010;132:922–3. https://doi.org/10.1021/ja909629f.

[99] Barrett SM, Wang C, Lin W. Oxygen sensing via phosphorescence quenching of doped metal–organic frameworks. J Mater Chem 2012;22:10329. https://doi.org/10.1039/c2jm15549d.

[100] Ho M-L, Chen Y-A, Chen T-C, Chang P-J, Yu Y-P, Cheng K-Y, et al. Synthesis, structure and oxygen-sensing properties of Iridium(iii)-containing coordination polymers with different cations. Dalton Trans 2012;41:2592. https://doi.org/10.1039/c2dt11473a.

[101] Qi X-L, Liu S-Y, Lin R-B, Liao P-Q, Ye J-W, Lai Z, et al. Phosphorescence doping in a flexible ultramicroporous framework for high and tunable oxygen sensing efficiency. ChemComm 2013;49:6864. https://doi.org/10.1039/c3cc43461c.

[102] Chen Y-T, Lin C-Y, Lee G-H, Ho M-L. Four new lead(II)–iridium(II) heterobimetallic coordination frameworks: synthesis, structures, luminescence and oxygen-sensing properties. CrystEngComm 2015;17:2129–40. https://doi.org/10.1039/C4CE02457E.

[103] Ye J-W, Lin J-M, Mo Z-W, He C-T, Zhou H-L, Zhang J-P, et al. Mixed-Lanthanide Porous Coordination Polymers Showing Range-Tunable Ratiometric Luminescence for O2 Sensing. Inorg Chem 2017;56:4238–43. https://doi.org/10.1021/acs.inorgchem.7b00252.

[104] Lin R-B, Li F, Liu S-Y, Qi X-L, Zhang J-P, Chen X-M. A Noble-Metal-Free Porous Coordination Framework with Exceptional Sensing Efficiency for Oxygen. Angew Chem 2013;125:13671–5. https://doi.org/10.1002/ange.201307217.

[105] Ye J-W, Zhou H-L, Liu S-Y, Cheng X-N, Lin R-B, Qi X-L, et al. Encapsulating Pyrene in a Metal–Organic Zeolite for Optical Sensing of Molecular Oxygen. Chem Mater 2015;27:8255–60. https://doi.org/10.1021/acs.chemmater.5b03955.

[106] Lin R-B, Zhou H-L, He C-T, Zhang J-P, Chen X-M. Tuning oxygen-sensing behaviour of a porous coordination framework by a guest fluorophore. Inorg Chem Front 2015;2:1085–90. https://doi.org/10.1039/C5QI00157A.

[107] Zhao Z, Ru J, Zhou P, Wang Y, Shan C, Yang X, et al. A smart nanoprobe based on a gadolinium complex encapsulated by ZIF-8 with enhanced room temperature phosphorescence for synchronous oxygen sensing and photodynamic therapy. Dalton Trans 2019;48:16952–60. https://doi.org/10.1039/C9DT03955D.

[108] Knedel T-O, Buss S, Maisuls I, Daniliuc CG, Schlüsener C, Brandt P, et al. Encapsulation of Phosphorescent Pt(II) Complexes in Zn-Based Metal–Organic Frameworks toward Oxygen-Sensing Porous Materials. Inorg Chem 2020;59:7252–64. https://doi.org/10.1021/acs.inorgchem.0c00678.

[109] Xie J, Chen X, Li H, Chen Z. On bio-MOF materials doped with phosphorescent iridium complexes for molecular oxygen determination: Synthesis, characterization and performance. Spectrochim Acta A Mol Biomol Spectrosc 2021;261:120041. https://doi.org/10.1016/j.saa.2021.120041.

[110] Dong X-Y, Si Y, Yang J-S, Zhang C, Han Z, Luo P, et al. Ligand engineering to achieve enhanced ratiometric oxygen sensing in a silver cluster-based metal-organic framework. Nat Commun 2020;11:3678. https://doi.org/10.1038/s41467-020-17200-w.

[111] Xia T, Jiang L, Zhang J, Wan Y, Yang Y, Gan J, et al. A fluorometric metal-organic framework oxygen sensor: from sensitive powder to portable optical fiber device. Microporous Mesoporous Mater 2020;305:110396. https://doi.org/10.1016/j.micromeso.2020.110396.

[112] Xu X-Y, Yan B. Nanoscale LnMOF-functionalized nonwoven fibers protected by a polydimethylsiloxane coating layer as a highly sensitive ratiometric oxygen sensor. J Mater Chem C 2016;4:8514–21. https://doi.org/10.1039/C6TC02569B.

[113] Liu S-Y, Qi X-L, Lin R-B, Cheng X-N, Liao P-Q, Zhang J-P, et al. Porous Cu(I) Triazolate Framework and Derived Hybrid Membrane with Exceptionally High Sensing Efficiency for Gaseous Oxygen. Adv Funct Mater 2014;24:5866–72. https://doi.org/10.1002/adfm.201401125.

[114] Feng D, Chung W-C, Wei Z, Gu Z-Y, Jiang H-L, Chen Y-P, et al. Construction of Ultrastable Porphyrin Zr Metal–Organic Frameworks through Linker Elimination. J Am Chem Soc 2013;135:17105–10. https://doi.org/10.1021/ja408084j.

[115] Yang J, Wang Z, Li Y, Zhuang Q, Gu J. Real-Time Monitoring of Dissolved Oxygen with Inherent Oxygen-Sensitive Centers in Metal–Organic Frameworks. Chem Mater 2016;28:2652–8. https://doi.org/10.1021/acs.chemmater.6b00016.

[116] Lan G, Ni K, You E, Wang M, Culbert A, Jiang X, et al. Multifunctional Nanoscale Metal–Organic Layers for Ratiometric pH and Oxygen Sensing. J Am Chem Soc 2019;141:18964–9. https://doi.org/10.1021/jacs.9b11024.

[117] Vander Donckt E, Camerman B, Hendrick F, Heme R, Vandeloise R. Polystyrene Immobilized Ir(III) Complex as a new Material for Optical Oxygen Sensing. Bull Soc Chim Belges 1994;103:207–11. https://doi.org/10.1002/bscb.19941030507.

[118] Mao Y, Mei Z, Wen J, Li G, Tian Y, Zhou B, et al. Honeycomb structured porous films from a platinum porphyrin-grafted poly(styrene-co-4-vinylpyridine) copolymer as an optical oxygen sensor. Sens Actuators B Chem 2018;257:944–53. https://doi.org/10.1016/j.snb.2017.11.042.

[119] Mao Y, Zhao Q, Pan T, Shi J, Jiang S, Chen M, et al. Platinum porphyrin/3-(trimethoxysily)propylmethacrylate functionalized flexible PDMS micropillar arrays as optical oxygen sensors. New J Chem 2017;41:5429–35. https://doi.org/10.1039/C7NJ01187C.

[120] Banerjee S, Arzhakova OV, Dolgova AA, Papkovsky DB. Phosphorescent oxygen sensors produced from polyolefin fibres by solvent-crazing method. Sens Actuators B Chem 2016;230:434–41. https://doi.org/10.1016/j.snb.2016.02.062.

[121] McKeown NB, Budd PM. Polymers of intrinsic microporosity (PIMs): organic materials for membrane separations, heterogeneous catalysis and hydrogen storage. Chem Soc Rev 2006;35:675. https://doi.org/10.1039/b600349d.

[122] Low Z-X, Budd PM, McKeown NB, Patterson DA. Gas Permeation Properties, Physical Aging, and Its Mitigation in High Free Volume Glassy Polymers. Chem Rev 2018;118:5871–911. https://doi.org/10.1021/acs.chemrev.7b00629.

[123] Kameda M, Tezuka N, Hangai T, Asai K, Nakakita K, Amao Y. Adsorptive pressure-sensitive coatings on porous anodized aluminium. Meas Sci Technol 2004;15:489–500. https://doi.org/10.1088/0957-0233/15/3/001.

[124] Araki N, Amao Y, Funabiki T, Kamitakahara M, Ohtsuki C, Mitsuo K, et al. Optical oxygen-sensing properties of porphyrin derivatives anchored on ordered porous aluminium oxide plates. Photochem Photobiol Sci 2007;6:794. https://doi.org/10.1039/b618030b.

[125] Baron AE, Danielson JDS, Gouterman M, Wan JR, Callis JB, McLachlan B. Submillisecond response times of oxygen-quenched luminescent coatings. Rev Sci Instrum 1993;64:3394–402. https://doi.org/10.1063/1.1144310.

[126] Amao Y, Miyakawa K, Okura I. Novel optical oxygen sensing device: a thin film of a palladium porphyrin with a long alkyl chain on an alumina plate. J Mater Chem 2000;10:305–8. https://doi.org/10.1039/a906666g.

[127] Amao Y, Okura I. An oxygen sensing system based on the phosphorescence quenching of metalloporphyrin thin film on alumina plates. Analyst 2000;125:1601–4. https://doi.org/10.1039/b004065g.

[128] Amao Y, Ishikawa Y, Okura I, Miyashita T. Optical Oxygen Sensing Material: Terbium(III) Complex Adsorbed Thin Film. Bull Chem Soc Jpn 2001;74:2445–9. https://doi.org/10.1246/bcsj.74.2445.

[129] Fernández-Sánchez JF, Cannas R, Spichiger S, Steiger R, Spichiger-Keller UE. Novel nanostructured materials to develop oxygen-sensitive films for optical sensors. Anal Chim Acta 2006;566:271–82. https://doi.org/10.1016/j.aca.2006.03.021.

[130] Marin-Suarezdel Toro M, Fernandez-Sanchez JF, Baranoff E, Nazeeruddin MdK, Graetzel M, Fernandez-Gutierrez A. Novel luminescent Ir(III) dyes for developing highly sensitive oxygen sensing films. Talanta 2010;82:620–6. https://doi.org/10.1016/j.talanta.2010.05.018.

[131] McDonagh C, Kolle C, McEvoy AK, Dowling DL, Cafolla AA, Cullen SJ, et al. Phase fluorometric dissolved oxygen sensor. Sens Actuators B Chem 2001;74:124–30. https://doi.org/10.1016/S0925-4005(00)00721-8.

[132] Lehner P, Staudinger C, Borisov SM, Regensburger J, Klimant I. Intrinsic Artefacts in Optical Oxygen Sensors-How Reliable are our Measurements? Chem Eur J 2015;21:3978–86. https://doi.org/10.1002/chem.201406037.

[133] Langsam M, Robeson LM. Substituted propyne polymers?part II. Effects of aging on the gas permeability properties of poly[1-(trimethylsilyl)propyne] for gas separation membranes. Polym Eng Sci 1989;29:44–54. https://doi.org/10.1002/pen.760290109.

[134] Müller BJ, Burger T, Borisov SM, Klimant I. High performance optical trace oxygen sensors based on NIR-emitting benzoporphyrins covalently coupled to silicone matrixes. Sens Actuators B Chem 2015;216:527–34. https://doi.org/10.1016/j.snb.2015.04.067.

[135] Larsen M, Lehner P, Borisov SM, Klimant I, Fischer JP, Stewart FJ, et al. In situ quantification of ultra-low O2 concentrations in oxygen minimum zones: Application of novel optodes: In situ trace sensing of O2 using novel optodes. Limnol Oceanogr Methods 2016;14:784–800. https://doi.org/10.1002/lom3.10126.

[136] Borisov SM, Klimant I. Ultrabright Oxygen Optodes Based on Cyclometalated Iridium(III) Coumarin Complexes. Anal Chem 2007;79:7501–9. https://doi.org/10.1021/ac0710836.

[137] Velasco-García N, Pereiro-García R, Diaz-García ME. Analytical and mechanistic aspects of the room temperature phosphorescence of Erythrosine B adsorbed on solid supports as oxygen sensing phases. Spectrochimica Acta Part A: Molecular and Biomolecular Spectroscopy 1995;51:895–904. https://doi.org/10.1016/0584-8539(94)00129-Y.

[138] Chan C-M, Chan M-Y, Zhang M, Lo W, Wong K-Y. The performance of oxygen sensing films with ruthenium-adsorbed fumed silica dispersed in silicone rubber. Analyst 1999;124:691–4. https://doi.org/10.1039/a900367c.

[139] Zhang P, Guo J, Wang Y, Pang W. Incorporation of luminescent tris(bipyridine)ruthenium(II) complex in mesoporous silica spheres and their spectroscopic and oxygen-sensing properties. Mater Lett 2002;53:400–5. https://doi.org/10.1016/S0167-577X(01)00514-6.

[140] Chu CS. Optical Fiber Oxygen Sensor Based on Ru (II) Complex and Porous Silica Nanoparticles Embedded in Sol-Gel Matrix. KEM 2012;516:612–7. https://doi.org/10.4028/www.scientific.net/KEM.516.612.

[141] Chu C-S, Sung T-W, Lo Y-L. Enhanced optical oxygen sensing property based on Pt(II) complex and metal-coated silica nanoparticles embedded in sol–gel matrix. Sens Actuators B Chem 2013;185:287–92. https://doi.org/10.1016/j.snb.2013.05.011.

[142] Zhang H, Li B, Lei B, Li W. Oxygen-sensing materials based on [Ru(bpy)3]2+ covalently grafted MSU-3 mesoporous molecular sieves. J Lumin 2008;128:1331–8. https://doi.org/10.1016/j.jlumin.2007.12.043.

[143] Costa-Fernández JM, Diaz-García ME, Sanz-Medel A. Sol–gel immobilized room-temperature phosphorescent metal-chelate as luminescent oxygen sensing material. Anal Chim Acta 1998;360:17–26. https://doi.org/10.1016/S0003-2670(98)00022-1.

[144] Klimant I, Ruckruh F, Liebsch G, Stangelmayer A, Wolfbeis OS. Fast Response Oxygen Micro-Optodes Based on Novel Soluble Ormosil Glasses. Microchim Acta 1999;131:35–46. https://doi.org/10.1007/s006040050007.

[145] Malins C, Glever HG, MacCraith BD, Fanni S, Vos JG. The preparation of a sol–gel glass oxygen sensor incorporating a covalently bound fluorescent dye. Anal Commun 1999;36:3–4. https://doi.org/10.1039/a808731h.

[146] Badia R, Garcia MED. Tuning the Performance of Room Temperature Phosphorescence Sensing Materials for Oxygen Using Factorial Designs. Anal Lett 2000;33:307–22. https://doi.org/10.1080/00032710008543054.

[147] Chan MA, Lawless JL, Lam SK, Lo D. Fiber optic oxygen sensor based on phosphorescence quenching of erythrosin B trapped in silica-gel glasses. Anal Chim Acta 2000;408:33–7. https://doi.org/10.1016/S0003-2670(99)00849-1.

[148] Lam SK, Chan MA, Lo D. Characterization of phosphorescence oxygen sensor based on erythrosin B in sol–gel silica in wide pressure and temperature ranges. Sens Actuators B Chem 2001;73:135–41. https://doi.org/10.1016/S0925-4005(00)00695-X.

[149] Basu BJ. Optical oxygen sensing based on luminescence quenching of platinum porphyrin dyes doped in ormosil coatings. Sens Actuators B Chem 2007;123:568–77. https://doi.org/10.1016/j.snb.2006.09.062.

[150] Chu C-S, Lo Y-L, Sung T-W. Enhanced oxygen sensing properties of Pt(II) complex and dye entrapped core–shell silica nanoparticles embedded in sol–gel matrix. Talanta 2010;82:1044–51. https://doi.org/10.1016/j.talanta.2010.06.020.

[151] Zaharieva J, Milanova M, Todorovsky D. SiO2/polyester hybrid for immobilization of Ru(ii) complex as optical gas-phase oxygen sensor. J Mater Chem 2011;21:4893. https://doi.org/10.1039/c0jm03169k.

[152] Chu C-S, Chuang C-Y. Highly sensitive fiber-optic oxygen sensor based on palladium tetrakis (4-carboxyphenyl)porphyrin doped in ormosil. J Lumin 2014;154:475–8. https://doi.org/10.1016/j.jlumin.2014.05.025.

[153] Önal E, Saß S, Hurpin J, Ertekin K, Topal SZ, Kumke MU, et al. Lifetime-Based Oxygen Sensing Properties of palladium(II) and platinum(II) meso-tetrakis(4-

phenylethynyl)phenylporphyrin. J Fluoresc 2017;27:861–8. https://doi.org/10.1007/s10895-016-2022-x.

[154] Mao Y, Liu Z, Liang L, Zhou Y, Qiao Y, Mei Z, et al. Silver Nanowire-Induced Sensitivity Enhancement of Optical Oxygen Sensors Based on AgNWs–Palladium Octaethylporphine–Poly(methyl methacrylate) Microfiber Mats Prepared by Electrospinning. ACS Omega 2018;3:5669–77. https://doi.org/10.1021/acsomega.8b00115.

[155] Oguzlar S. Development of highly sensitive [Ru(bpy)3]2+ - Based optical oxygen sensing thin films in the presence with Fe3O4 and Fe3O4@Ag NPs. Opt Mat 2020;101:109772. https://doi.org/10.1016/j.optmat.2020.109772.

[156] Xu Y, Yang D, Huo S, Ren J, Gao N, Chen Z, et al. Carbon dots and ruthenium doped oxygen sensitive nanofibrous membranes for monitoring the respiration of agricultural products. Polymer Testing 2021;93:106957. https://doi.org/10.1016/j.polymertesting.2020.106957.

[157] Amao Y, Asai K, Okura I, Shinohara H, Nishide H. Platinum porphyrin embedded in poly(1-trimethylsilyl-1-propyne) film as an optical sensor for trace analysis of oxygen. Analyst 2000;125:1911–4. https://doi.org/10.1039/b005838f.

[158] Amao Y, Okura I, Shinohara H, Nishide H. An Optical Sensing Material for Trace Analysis of Oxygen. Metalloporphyrin Dispersed in Poly(1-trimethylsilyl-1-propyne) Film. Polym J 2002;34:411–7. https://doi.org/10.1295/polymj.34.411.

[159] Hyakutake T, Ishigami Y, Kato J, Inukai J, Miyatake K, Nishide H, et al. Luminescent Sensory Polymer Coating Composed of Platinumporphyrin and Poly(trimethylsilylpropyne) for Real-Time Oxygen Visualization in Operating PEFCs. Macromol Chem Phys 2011;212:42–7. https://doi.org/10.1002/macp.201000449.

[160] Mills A, Graham A, O’Rourke C. A novel, titania sol–gel derived film for luminescence-based oxygen sensing. Sens Actuators B Chem 2014;190:907–12. https://doi.org/10.1016/j.snb.2013.08.097.

[161] McGee KA, Veltkamp DJ, Marquardt BJ, Mann KR. Porous Crystalline Ruthenium Complexes Are Oxygen Sensors. J Am Chem Soc 2007;129:15092–3. https://doi.org/10.1021/ja0681772.

[162] McGee KA, Marquardt BJ, Mann KR. Concurrent Sensing of Benzene and Oxygen by a Crystalline Salt of Tris(5,6-dimethyl-1,10-phenanthroline)ruthenium(II). Inorg Chem 2008;47:9143–5. https://doi.org/10.1021/ic801287p.

[163] McGee KA, Mann KR. Inefficient Crystal Packing in Chiral [Ru(phen)3](PF6)2 Enables Oxygen Molecule Quenching of the Solid-State MLCT Emission. J Am Chem Soc 2009;131:1896–902. https://doi.org/10.1021/ja8075605.

[164] Meier B, Werner T, Klimant I, Wolfbeis OS. Novel oxygen sensor material based on a ruthenium bipyridyl complex encapsulated in zeolite Y: dramatic differences in the efficiency of luminescence quenching by oxygen on going from surface-adsorbed to zeolite-encapsulated fluorophores. Sens Actuators B Chem 1995;29:240–5. https://doi.org/10.1016/0925-4005(95)01689-9.
